# Dye Induced Luminescence Properties of Gold(I) Complexes with near Unity Quantum Efficiency

**DOI:** 10.1002/anie.202414517

**Published:** 2024-10-22

**Authors:** Vanitha R. Naina, Sebastian Gillhuber, Christian Ritschel, Da Jin, Shubham, Sergei Lebedkin, Claus Feldmann, Florian Weigend, Manfred M. Kappes, Peter W. Roesky

**Affiliations:** ^1^ Institute of Inorganic Chemistry Karlsruhe Institute of Technology Engesserstraße 15 76131 Karlsruhe Germany; ^2^ Institute of Nanotechnology Karlsruhe Institute of Technology Hermann-von-Helmholtz-Platz 1, Eggenstein-Leopoldshafen 76344 Karlsruhe Germany; ^3^ Fachbereich Chemie Philipps-Universität Marburg Hans-Meerwein-Straße 4 35032 Marburg Germany; ^4^ Institute of Physical Chemistry Karlsruhe Institute of Technology Fritz-Haber-Weg 2 76131 Karlsruhe Germany

**Keywords:** Coumarin, Gold complexes, N-Heterocyclic carbenes, Phosphines, Quantum yield

## Abstract

To study the effect of a dye on the photoluminescence (PL) properties of metal complexes, a series of gold(I) complexes were synthesized, containing a 7‐amino‐4‐methylcoumarin luminophore. The complexes are comprised of a coumarin moiety featuring different ancillary ligands, specifically N‐heterocyclic carbenes, triphenylphosphine, and diphenyl‐2‐pyridylphosphine. The synthesized gold(I) complexes are luminescent both in solution and the solid state at room temperature and 77 K. Complexes of different nuclearity, i.e., mono‐, di‐ and trinuclear compounds were synthesized. A clear trend between the nuclearity and the quantum yields can be seen. The coumarin dye not only improves the PL properties, but also enhances the luminescence of trinuclear clusters, which are otherwise known to be weak emitters in solution. The optical absorption properties were investigated in detail by quantum chemical calculations.

## Graphical abstract

**Improving the glow of gold complexes**. Herein, we report a facile synthetic strategy to introduce a dye, namely, 7‐amino‐4‐methylcoumarin, as a ligand to access luminescent gold complexes of different nuclearity. Introduction of the dye enables the isolation of gold complexes with rich photophysical properties such as high emission quantum yields.

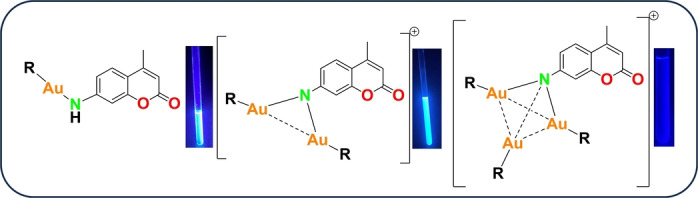

## Introduction

Luminescent gold(I) complexes gained significant attention since the first report of emission from [PPh_3_AuCl] by Dori and co‐workers in 1970.^[1]^ In recent years, gold complexes with rich photophysical properties such as high quantum yields and long lifetimes have been explored for potential applications across various fields such as bioimaging, optoelectronics and photocatalysis.[^2^, ^3^, ^4^, ^5^, ^6^, ^7^, ^8^] The ligands featured in these luminophores are mainly based on phosphines, carbenes, acetylides, amidinates and amides.[^9^, ^10^, ^11^, ^12^, ^13^, ^14^, ^15^, ^16^]

**Figure 1:**
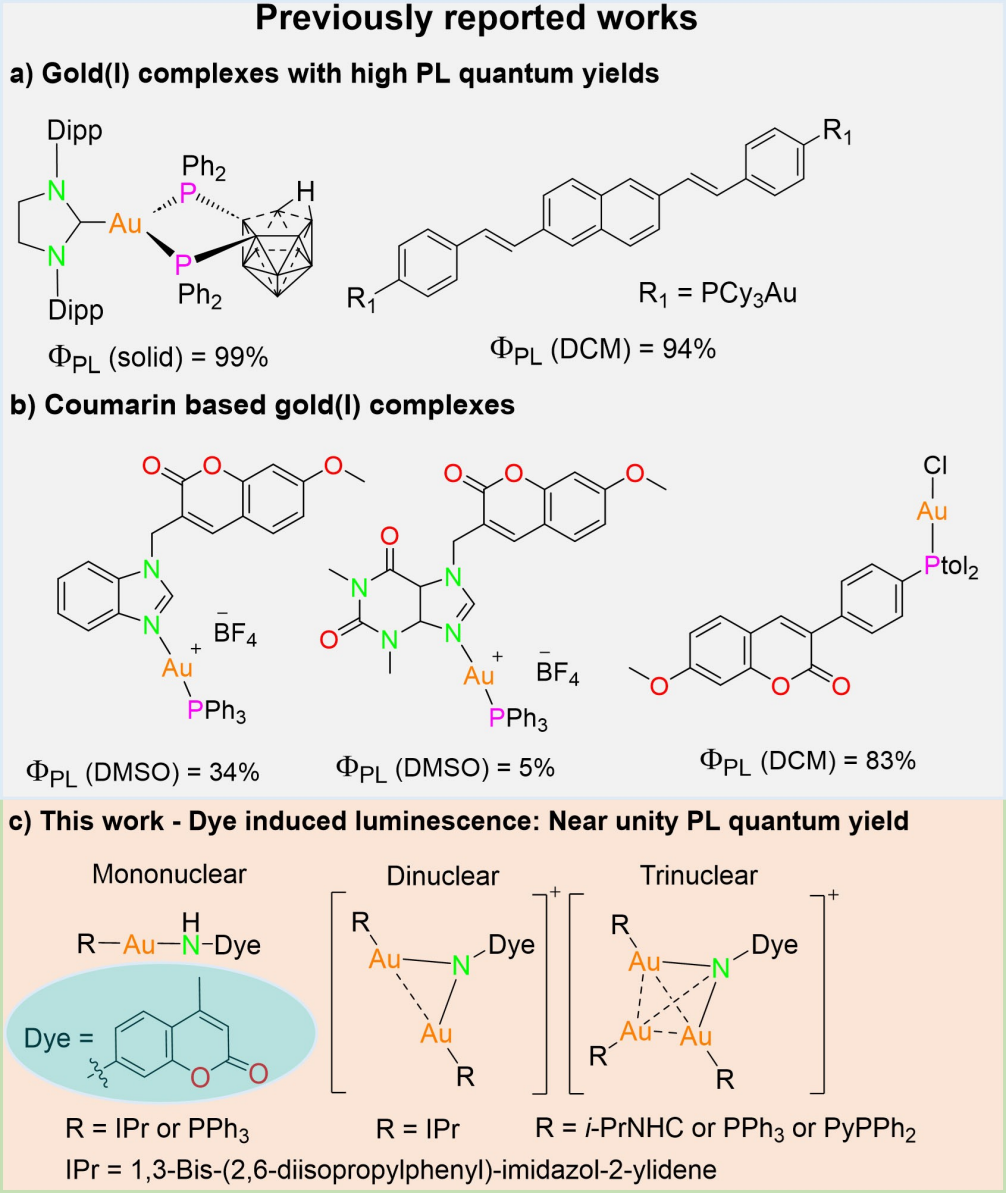
a) Previously reported gold(I) complexes with high quantum yields[^18^, ^19^] b) Examples of coumarin based gold(I) complexes[^22^, ^24^] c) Overview of this work.

It is now well‐known that the ligand backbone significantly alters the properties of the resulting metal complexes. Understanding the intricacies of ligand‐metal interactions enabled researchers to fine‐tune the behavior of metal complexes. For instance, Laguna, Eisenberg and co‐workers used diphenyl‐2‐pyridylphosphine (PyPPh_2_) coordinated gold chalcogenide complexes to access luminescent bimetallic clusters with color‐tunable emission.^[17]^ Gimeno and co‐workers reported a three‐coordinate NHC gold(I) complex with a quantum yield (Φ_PL_) of 99 % in the solid state (Figure 1a).^[18]^ Gray and co‐workers showed that gold(I) substituted distyrylnaphthalene can emit with Φ_PL_ up to 94 % (Figure 1a).^[19]^ These works clearly emphasize the paramount importance of ligands in designing metal complexes with tailored properties. Recently, we have shown that introducing a dye into the ligand backbone can result in rich optical properties.[^20^, ^21^] Specifically, we functionalized the amino group of 7‐amino‐4‐methylcoumarin with a phosphine group to obtain an aminodiphosphine ligand. The ligand was used to synthesize copper and silver complexes of different nuclearity. The resulting complexes exhibit second‐long afterglow properties at low temperatures.^[20]^ However, these complexes are very poor emitters at room temperature.

Even though coumarin based gold(I) complexes emerged as potential fluorescent probes for bioimaging, only a handful works are reported till date.[^22^, ^23^, ^24^, ^25^, ^26^] Among these compounds, only a coumarin phosphine substituted gold(I) complex showed Φ_PL_ of 83 % (Figure 1b).^[22]^ Probes with high Φ_PL_ are particularly of interest as they offer better imaging signals with lower dye loading, avoiding cytotoxicity issues and biological pathway interference.^[27]^ Therefore, we herein extend our previous work by replacing the coumarin amino protons by LAu^+^ units (following Hoffmann's isolobal analogy, L=ligand).^[28]^ This modification results in luminescent gold complexes with high emission quantum yields at room temperature. Furthermore, the influence of metal loading on the emission properties is systematically investigated in this work. Chao and co‐workers also studied the effect of nuclearity on photoluminescence (PL) properties using dansylamide and PPh_3_ as ligands. However, the quantum yields in solution were less than 30 %,^[29]^ which gives room for improvement.

Among the trinuclear gold complexes (based on pyrazolate and carbeniate ligands), only planar complexes, which form intermolecular stacking arrangements, were known to be luminescent.[^30^, ^31^, ^32^] Recently, Laguna, Eisenberg and co‐workers showed that incorporating a second metal in the trinuclear compounds can result in emission properties, intrinsic to the metal complexes (without any intermolecular stacking interactions).^[17]^ However, the emission of the clusters was only reported in the solid state. In this work, we introduce a series of mononuclear to trinuclear Au^I^ complexes featuring a coumarin moiety and ancillary NHC or phosphine ligands (Figure 1c). In the present study, we show that embedding coumarin to polynuclear gold complexes results in compounds emissive both in the solution and solid state. These new findings are expected to lead to many novel coumarin‐based metal complexes with intriguing properties.

## Results and Discussion

### Synthesis and Characterization

**Scheme 1:**
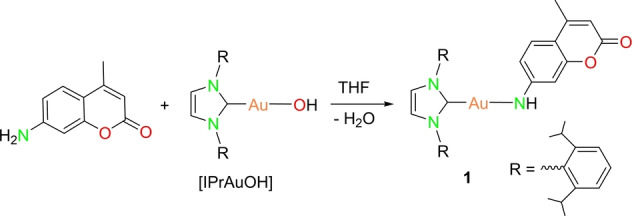
Synthesis of IPr (IPr=1,3‐Bis‐(2,6‐diisopropylphenyl)‐imidazol‐2‐ylidene) coordinated mononuclear gold complex **1**.

**Figure 2:**
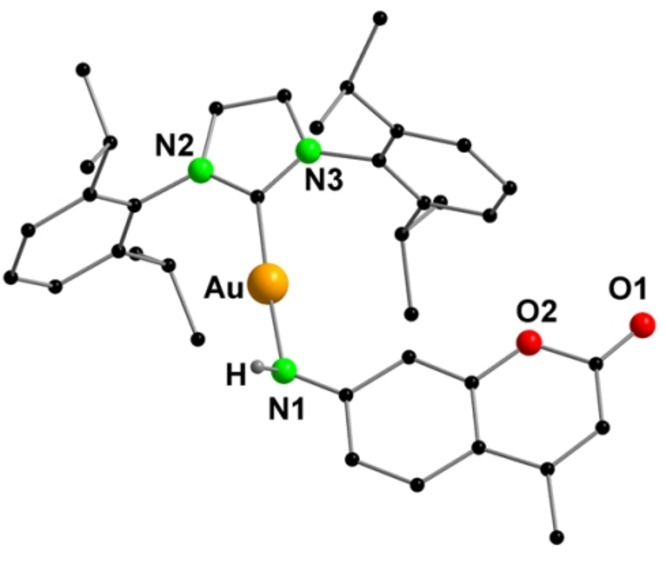
Molecular structure of IPr coordinated gold imido complex **1** in the solid state. Hydrogen atoms (except N−H proton) and non‐coordinating solvents are removed for clarity. Structural parameters are given in the ESI (Figure S30).^[33]^

A series of coumarin‐embedded gold complexes of different nuclearity, specifically mono‐, di‐ and trinuclear compounds were synthesized first, followed by the investigation of their photophysical properties. The reaction of [IPrAuOH] (IPr=1,3‐Bis‐(2,6‐diisopropylphenyl)‐imidazol‐2‐ylidene) with 7‐amino‐4‐methylcoumarin in an equimolar ratio yielded the mononuclear gold complex [(Coum)AuIPr] (**1**) (Scheme 1). In the solid state, the gold(I) cation is linearly coordinated (∠C−Au−N: 176.8(3)°) by IPr and the coumarin moiety (Figure 2). The Au−N distance to the coumarin and Au−C distance to the carbene are 1.981(6) Å and 1.997(6) Å, respectively. The N*C*N carbon signal of **1** is downfield‐shifted from *δ*=171.9 ppm ([IPrAuOH]) to *δ*=177.4 ppm in the ^13^C{^1^H} NMR spectrum.

**Scheme 2:**
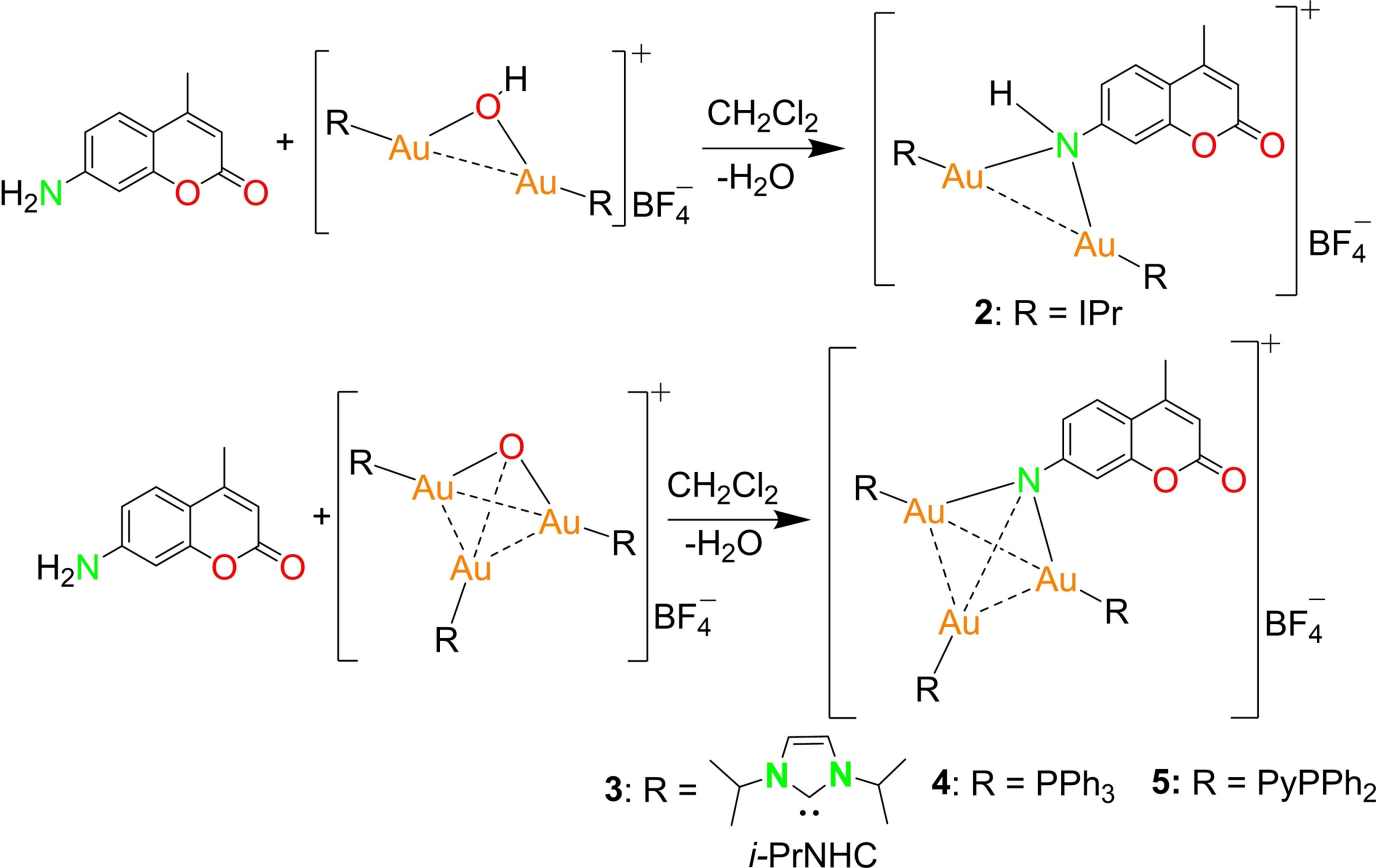
Synthesis of dinuclear complex **2** (IPr=1,3‐Bis‐(2,6‐diisopropylphenyl)‐imidazol‐2‐ylidene) and trinuclear complexes **3**, **4** and **5**.

The dinuclear gold hydroxo complex [(IPrAu)_2_OH][BF_4_] was reacted with the coumarin in an equimolar ratio (Scheme 2). As expected, the reaction yielded the dinuclear gold imido complex [Coum(AuIPr)_2_][BF_4_] (**2**) as confirmed by the shift of the carbon signal (N*C*N) from *δ*=170.2 ppm [(IPrAu)_2_OH][BF_4_] to *δ*=171.0 ppm in the ^13^C{^1^H} NMR spectrum and the appearance of proton signals of the coumarin moiety in the ^1^H NMR spectrum. Additionally, the molecular ion peak at *m/z*=1344.5544 (calculated: 1344.5638) confirms the successful synthesis of the desired product and its stability in solution (Figure S26). However, multiple attempts to isolate single crystals of complex **2** were not successful.

The nuclearity of the complexes could not be increased further due to the steric bulkiness of the IPr ligand. Hence, *i*‐PrNHC (1,3‐bis‐(isopropyl)‐imidazol‐2‐ylidene) was employed as a ligand to synthesize the gold oxonium cluster [(*i‐*PrNHC)_3_Au_3_O], which was used as a precursor. Further reaction of this gold oxonium cluster with the coumarin resulted in an aurated imido complex [Coum{Au(*i‐*PrNHC)}_3_][BF_4_] (**3**) (Scheme 2).

**Figure 3:**
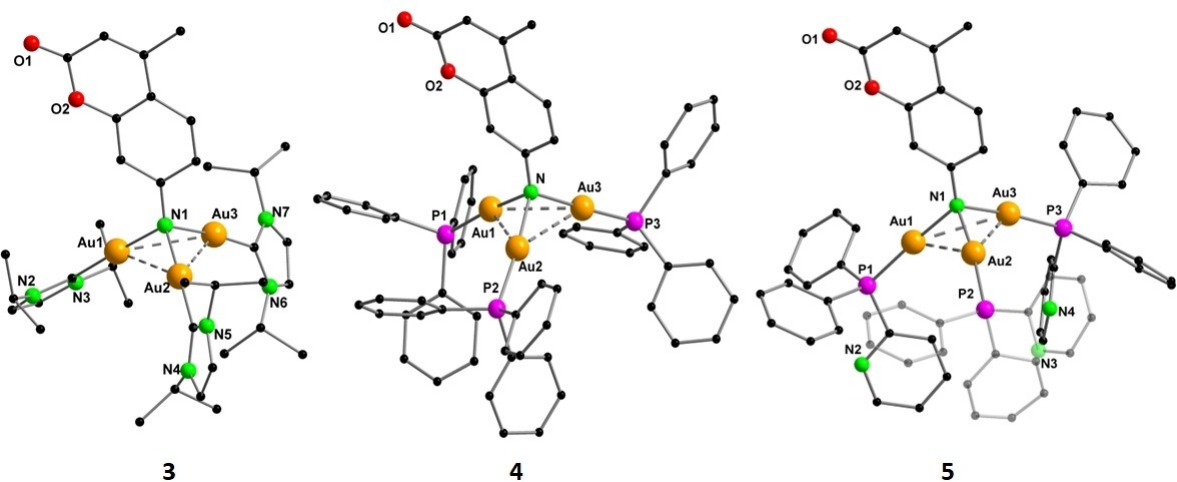
Molecular structure of complexes **3**, **4** and **5** in the solid state. Hydrogen atoms, counter anion BF_4_^−^ and the non‐coordinating solvent molecules are omitted for clarity. Structural parameters are given in the ESI (Figure S31–S33).^[33]^

The molecular structure features an Au_3_ imido core with each *i*‐PrNHC coordinated to an Au(I) cation and a BF_4_^−^ counteranion (Figure 3). The Au−Au separation [Au−Au: 3.0042(6)–3.1721(5) Å] in the Au_3_ triangle indicates the existence of aurophilic interactions. The Au(I) cations adopt almost a linear geometry with the angles on the individual Au(I) cations slightly deviating from 180°. Following a similar synthetic route with PPh_3_ and PyPPh_2_, two other trinuclear complexes [Coum(AuPPh_3_)_3_][BF_4_] (**4**) and [Coum(AuPyPPh_2_)_3_][BF_4_] (**5**) were synthesized (Scheme 2).

The molecular structure in the solid state of **4** reveals that the amino group of coumarin is triply aurated with Au−N distances in the range of 2.034(6)–2.057(6) Å (Figure 3). The Au−Au separation in the Au_3_ core varies between 2.9339(4)–3.2081(4) Å, indicating the existence of aurophilic interactions in accordance with the literature reports.^[29]^ The global charge of the cluster is balanced by a non‐coordinating BF_4_^−^ anion.

Complex **5** was isolated following a synthetic procedure similar to that of complex **4**, except for a PyPPh_2_ protected gold‐oxo cluster being used instead of PPh_3_ (Scheme 2) and crystallization being done by layering diethylether on a DCM solution of **5**. Single crystal X‐ray diffraction studies reveal that the complex has a tetrahedral gold imido core containing three gold atoms (Figure 3). The Au–Au and Au–N bond lengths are similar to those of complex **4** [Au–Au: 2.9752(4)–3.1998(5) Å and Au–N: 2.059(5)–2.068(5) Å].

The molecular structure of the gold clusters **3**–**5** in solution remains intact, as evidenced by NMR and HRESI‐MS. The existence of complex **3** in solution was evidenced by a shift in the carbon signal (N*C*N) from *δ*=159.9 ppm ([{(*i*‐PrNHC)Au}_3_O][BF_4_]) to *δ*=170.2 ppm in the ^13^C{^1^H} NMR spectrum. Complexes **4** and **5** exhibit a singlet at *δ*=28.3 ppm and *δ*=27.4 ppm in their respective ^31^P{^1^H} NMR spectrum, which are downfield‐shifted compared to their corresponding gold‐oxo precursors. The phosphorus signals appear at a similar position to reported analogous clusters.^[29]^ Further, the appearance of the molecular ion peak of complexes **3**, **4** and **5** in the respective HRESI‐MS spectra supports the intact presence of these gold clusters in solution (Figures S27–S29).

We have also attempted to synthesize mononuclear compounds with PPh_3_ and *i‐*PrNHC substituents. However, the desired products tend to rapidly decompose due to less steric shielding of the ligand, hence could not be isolated (Figure S1 and S2).

### Photophysical properties

**Figure 4:**
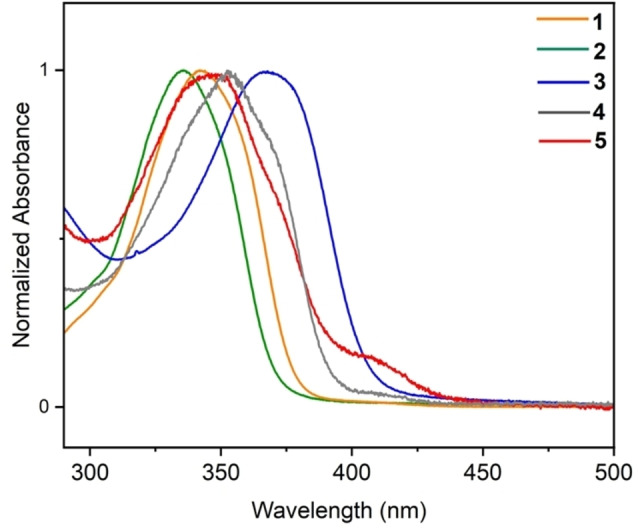
Normalized absorption spectra of complexes **1**–**5** recorded in DCM at room temperature.

The absorption spectra of the complexes **1**–**5** recorded in DCM are displayed in Figure 4. The mono‐ and dinuclear gold complexes **1** and **2** with IPr ligands feature an absorption band centered at ∼340 nm. The band is red‐shifted by *ca*. 35 nm for the trinuclear complex **3**. The phosphine‐coordinated trinuclear compounds also show a strong absorption shifted to *ca*. 350 nm.

Additionally, a low energy shoulder is also observed for these complexes, with an onset at 440 nm.

To gather a more detailed understanding of the absorption processes occurring in complexes **1**–**5**, time‐dependent density functional theory (TDDFT) calculations at the PBE0/def2‐TZVP level were performed with TURBOMOLE[^34^, ^35^] (refer to Supporting Information Chapter VII for computational details). Optimized structures of all compounds considered in the calculations can be found in the separate ASCII file optimized‐structures.txt as part of the SI. Selected calculated bond lengths and angles are summarized in Supporting Information Table S2 and are in good agreement with the experimental values.

Excitation energies and oscillator strengths were calculated for the ten lowest energy singlet vertical excitations of compounds **1**–**5**. The absorption spectra simulated based on these calculations are overlaid with the experimental spectra in Supporting Information Figure S44. Taking into account the known limitations of the TDDFT approach^[36]^ and the fact that the measurements were conducted in DCM solution at 298 K, while the calculations refer to individual molecules in the gas phase at 0 K, the calculated absorption maxima are in reasonable agreement with the experimental data, with deviations amounting to less than 40 nm in all cases.

For all compounds, the excitation occurs almost exclusively from the HOMO of the respective complex (Tables S4 and S6–S9) which corresponds to a π‐type orbital on the coumarin moiety (Figures S39 and S41–42) with small contributions of the Au ions (Tables S13–14 and S18–S22 for Mulliken population analyses^[37]^). For **1** and **2**, the lowest energy excitation corresponds to a HOMO–LUMO transition and dominates the absorption spectrum (Tables S4 and S6 and Figure S44). However, the shapes of the LUMOs differ significantly (refer to Supporting Information Figures S39 and S41). For **1**, the LUMO shows contributions of both, the coumarin and NHC ligand, while for **2** it is centered exclusively on the coumarin moiety. This is also reflected in the non‐relaxed difference densities (Figures S39 and S41), visualizing the metal‐mediated interligand charge‐transfer (ICT) character of the excitation for **1** and the coumarin‐centered transition for **2**, with the latter resembling the absorption of 7‐amino‐4‐methylcoumarin itself (Figure S38).

This can be rationalized when considering one major structural discrepancy between compounds **1** and **2**. For **1**, the planes defined by the ring atoms of the coumarin and the NHC ligand, respectively, are aligned in a parallel fashion, enabling the efficient metal‐mediated ICT between the coumarin and NHC π‐systems by maximizing the overlap between the metal‐centered and ligand‐centered orbitals. Contrary, for **2** steric constraints prevent the parallel orientation of the coumarin and NHC ligand planes, rendering the ICT mechanism less efficient, thus confining the excitation to the coumarin moiety.

In the extreme case of a perpendicular orientation of the coumarin and NHC ligand planes, there is no symmetry‐allowed pathway for the metal‐mediated ICT between the coumarin and NHC π‐systems. In line with this, rotation of the coumarin moiety in complex **1** by 90°, resulting in a perpendicular orientation of the coumarin and NHC ligand planes, also confines the excitation to the coumarin moiety, as is evident from the non‐relaxed difference density plot of the relevant transitions (Table S5 and Figure S40).

For the trinuclear complexes **3**–**5**, the situation is more intricate. While the experimental absorption spectra appear qualitatively similar to the ones of **1** and **2**, featuring mainly one broad absorption band, the calculations reveal that several energetically close lying excitations with similar oscillator strengths contribute to the absorption spectrum (Figure S44 and Tables S7–S9). As stated above, excitation mainly originates from the HOMO of the respective complex. However, apart from the LUMO, various further energetically low‐lying unoccupied orbitals are involved in these excitations (Tables S7–S9). The non‐relaxed difference density plots of the ten lowest energy excitations (Figure S42) indicate the ICT nature of the transitions, with electron density being transferred from the coumarin moiety to the ancillary ligands via the Au ions.

**Figure 5:**
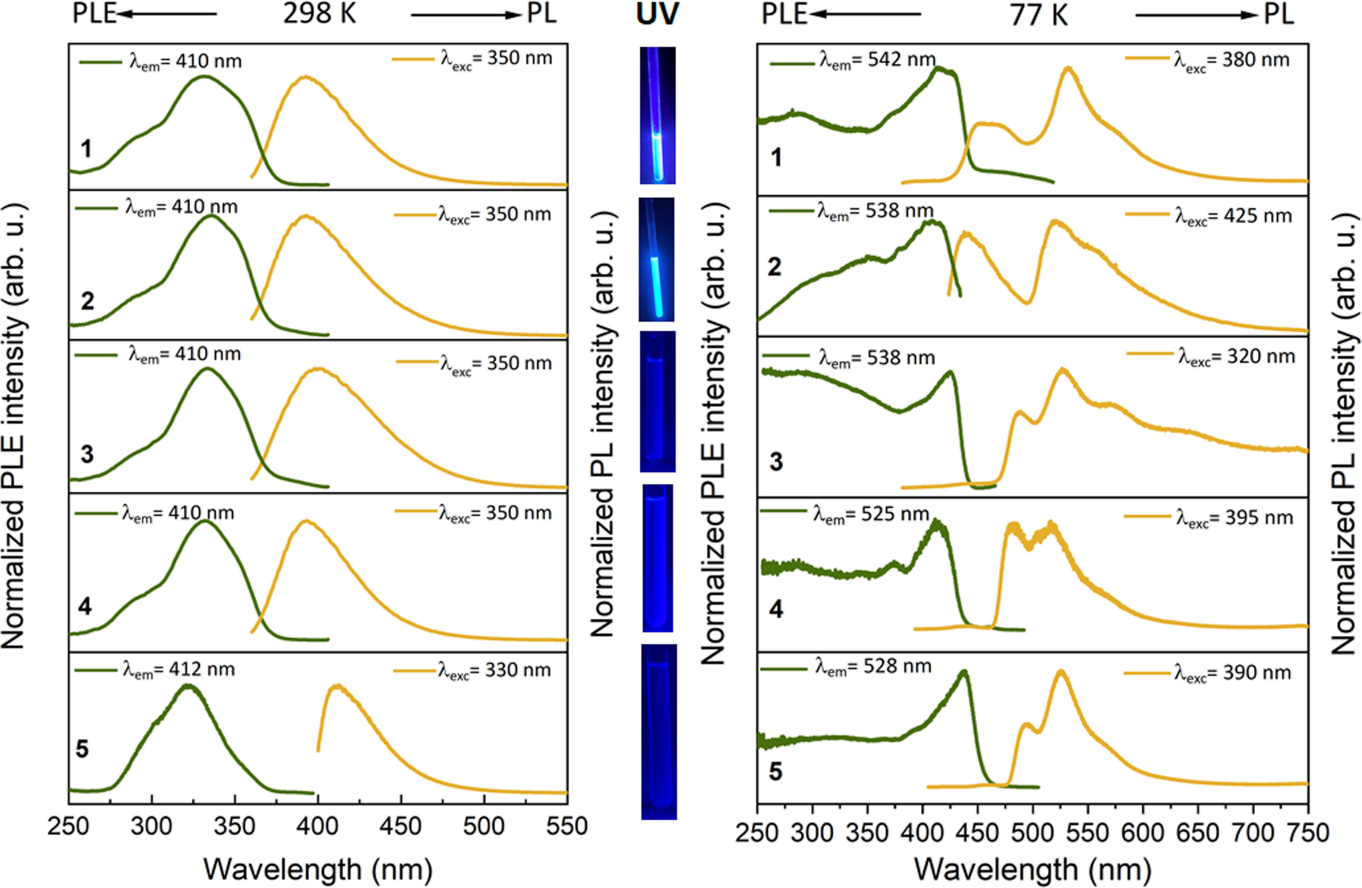
Normalized photoluminescence excitation (PLE) and emission (PL) spectra of DCM solutions of complexes **1**–**5** at room temperature and 77 K (left and right panels, respectively); photographs of DCM solutions of complexes under UV illumination at room temperature (centre). PLE and PL spectra were recorded at the depicted wavelengths (*λ*_em_ and *λ*_ex_).

Emission spectra of the complexes **1**–**5** recorded in DCM at 298 K are given in Figure 5, and the photoluminescence (PL) properties are listed in Table 1. All complexes show a similar fluorescence band at ca. 400 nm.

**Table 1:** Photoluminescence data of complexes **1**–**5** in DCM at room temperature and 77 K.

Complex	λ_max_ (nm)	Φ_PL_ (%)	τ (ns)	*k*_r_ (10^8^ s^−1^)	*k*_nr_ (10^8^ s^−1^)	λ_max_ (nm)	τ (μs)
DCM, RT						DCM, 77 K	
**1**	393	98	2.9	3.4	~0.1	460, 540 542	–*
**2**	393	57	2.3	2.5	1.7	455, 530	–*
**3**	401	20	5.2	0.4	1.5	538^a^	173^b^
**4**	394	21	1.6	1.3	5.0	524^a^	297^b^
**5**	412	7	2.9	0.2	3.2	524^a^	184^b^

* values were not determined, ^a^ spectral position of the most pronounced vibronic subband, ^b^ average lifetime from the biexponential decay fit.

The two‐fold coordinate complex **1** has an extraordinarily high PL efficiency (Φ_**PL**_) of 98 % in DCM solution, whereas 7‐amino‐4‐methylcoumarin emits with only Φ_**PL**_ of 79 % in DCM. To the best of our knowledge, such high Φ_**PL**_ is only reported for few gold(I) complexes in non‐polar solvents.[^12^, ^38^, ^39^, ^40^, ^41^] We note that except for one example,^[22]^ coumarin based gold(I) complexes also emit with low Φ_**PL**._[^23^, ^24^, ^25^, ^42^, ^43^] To enable comparability of our findings with some of the reported coumarin gold(I) complexes, we measured Φ_**PL**_ of **1** in DMSO and determined it to be 94 %. Additionally, carbazole‐based two‐coordinate coinage metal complexes usually emit from intramolecular charge transfer states.[^12^, ^40^, ^44^]

To test the metal influence on the emission of complex **1**, we synthesized the isostructural copper(I) compound **6** (Figure S34). While this results in similar fluorescence properties (refer to Figure S35), the quantum yield of this compound measured to be only 23 %. Additional quantum chemical calculations reveal that the absorption process is similar to that in complex **1**, with the absorption spectrum being dominated by a HOMO–LUMO transition of ICT character (Table S10 and Figure S43). However, the contribution of the Cu ion to the LUMO of **6** is significantly smaller than that of Au in compound **1** as is evident from Mulliken population analyses (Tables S14 and S24), thus rendering the ICT process less efficient. Consequently, the nature of the metal plays a key role for the PL properties. Moreover, a similar trend of lower quantum yield for a Cu^I^ complex compared to an analogous Au^I^ complex was observed in previous studies.^41^

The fluorescence lifetime of **1** in DCM is 2.9 ns, resulting in a high radiative rate constant (*k*_**r**_) of 3.4*10^8^ s^−1^ (Table 1). The lifetimes of the di‐ and trinuclear complexes vary between 1.6 and 5.2 ns. The Φ_**PL**_ of the carbene‐coordinated gold complexes is inversely proportional to the number of gold atoms. The decrease in Φ_**PL**_ follows the order **1**>**2**>**3**, which is also the order for *k*_**r**_. The *k*_**r**_ values decrease by ten‐fold for the trinuclear complexes **3** and **5** relative to **1**. Even though complex **4** has a high *k*_**r**_ value, non‐radiative transitions play a predominant role as indicated by the rate of non‐radiative decay (*k*_nr_=5.0 * 10^8^ s^−1^), thereby, resulting in low Φ_**PL**_. However, the Φ_**PL**_ of 20 % for complex **4** is a seven‐fold increase when compared to the similar trinuclear complex with dansylamide (Φ_**PL**_=3 %) instead of coumarin.^[29]^ When we replace the dansylamide and PPh_3_ ligands with the NHC and coumarin derivative, the resulting complex **3** exhibits a similar increase in PL efficiency. The enhanced non‐radiative decay rates for the di‐ and trinuclear complexes **2**–**5** compared to **1** are likely because of increased rotational and vibrational degrees of freedom introduced by an additional Au‐amide ligand bond.[^12^, ^45^]

Remarkable changes occur in the emission spectra of the complexes upon freezing and cooling their DCM solutions to 77 K (Figure 5). A bathochromic shift of the coumarin‐based fluorescence is observed for all complexes. The fluorescence of **1** consists now of two subbands at ca. 460 and 540 nm, which are separated by ca. 3200 cm^−1^ and show the same PLE spectra and decay traces. The above energy separation may be attributed to the N−H stretching vibration in the bridge between the gold atom and coumarin (Figure 2). The analogous copper(I) compound **6** does not show a second fluorescent band at 77 K (Figure S35). A similar kind of observation is also made for the dinuclear complex **2** which emits a similarly structured fluorescence in frozen DCM. In contrast to **1** and **2**, the major emission of the trinuclear complexes **3**–**5** in DCM at 77 K is phosphorescence with vibronically structured bands at 500–600 nm and decay kinetics on the order of hundreds of microseconds. It is tentatively attributed to metal‐metal‐to‐ligand charge‐transfer transitions (MMLCT).^[46]^ The coumarin‐based fluorescence is observed as a minor band at ca. 450 nm, making up only a few percent of the total emission. Accordingly, intersystem crossing (ISC) from the excited singlet to the triplet MMLCT state is quite efficient and dominates over fluorescence‐related transitions in **3**–**5** rigidly configured in frozen DCM solutions. The efficient ISC may also correlate with aurophilic interactions in **3**–**5**.[^47^, ^48^, ^49^]

**Figure 6:**
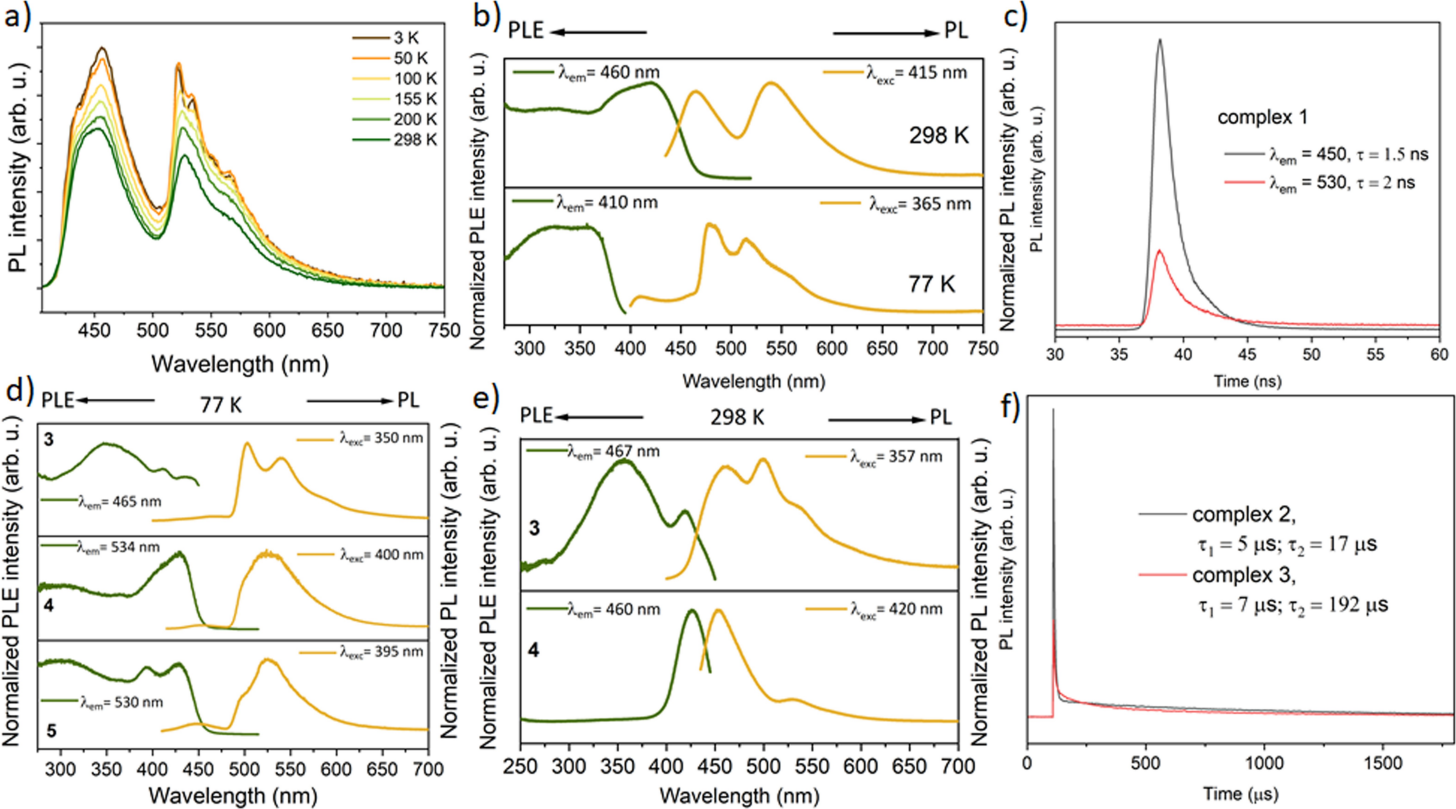
Temperature‐dependent emission (PL) and excitation (PLE) spectra and decay traces of solid (polycrystalline) complexes: a) mononuclear gold complex **1** (*λ*_exc_=400 nm); b) dinuclear complex **2** at 298 K and 77 K; c) fluorescence decay of **1** (*λ*_exc_=371 nm); d) trinuclear complexes **3**–**5** at 77 K and e) at room temperature (complex **5** is non‐emissive at room temperature); f) phosphorescence decay of **2** and **3** at 77 K (*λ*_exc_=371 nm); (PLE and PL spectra were recorded at the depicted wavelengths *λ*_em_ and *λ*_exc_, respectively).

The PL properties of solid (polycrystalline) complexes **1**–**5** (Figure 6) in general correspond to those found in frozen DCM solutions as described above. For instance, the mononuclear complex **1** features “two‐band” fluorescence, both at room and low temperatures (Figure 6a, c), assigned to the ~3200 cm^−1^ vibronic splitting (see above). The solid dinuclear complex **2** emits the coumarin‐based fluorescence at ca. 410 nm at room temperature (Figure 6b) and minor phosphorescence bands at 480–550 nm, which increase in intensity with decreasing temperature and become dominant at 77 K. The PL patterns of solid trinuclear complexes **3**–**5** at low temperatures are quite close to those in frozen DCM solutions (Figure 5), with the phosphorescence bands at about 500–600 nm, minor fluorescence at ca. 450 nm (Figure 6d), and similar decay timescales. A contribution of the coumarin‐based fluorescence increases, however, in **3** and **4** by raising the temperature and becomes comparable/dominant relative to the phosphorescence at 298 K (like in **2**). Contrary to **3** and **4**, the PyPPh_2_‐coordinated complex **5** does not show any appreciable luminescence in the solid state at room temperature.

### Conclusion

We report a new strategy to enhance the photophysical properties of gold(I) complexes by incorporating a coumarin dye. Specifically, we have achieved the facile synthesis of several gold complexes of different nuclearities comprising a coumarin dye and ancillary NHC and phosphine ligands. These complexes feature complicated and rich photophysical properties due to the coumarin dye.

Mono‐ and dinuclear complexes bearing IPr ligands feature high Φ_**PL**_ values of 98 % and 57 % in polar solvents like DCM and DMSO (94 % for complex **1**). The emission from these complexes is fluorescent in nature. The emission properties are strongly influenced by the number of gold atoms. The trinuclear complexes feature aurophilic interactions, and the emissive excited states decay on the microsecond timescale. The quantum efficiency of the trinuclear compounds is low compared to the mono‐ and dinuclear complexes. However, the PL efficiency of the trinuclear compounds in solution, which is typically very low, is significantly improved due to the coumarin dye. The rich photophysical properties and the high quantum efficiencies in polar solvents make these complexes intriguing candidates for the fabrication of organic light emitting diodes and their application in bio‐imaging techniques.

## Conflict of Interests

The authors declare no conflict of interest.

1

## Supporting information

As a service to our authors and readers, this journal provides supporting information supplied by the authors. Such materials are peer reviewed and may be re‐organized for online delivery, but are not copy‐edited or typeset. Technical support issues arising from supporting information (other than missing files) should be addressed to the authors.

Supporting Information

Supporting Information

Supporting Information

Supporting Information

Supporting Information

## Data Availability

The data that support the findings of this study are available in the supplementary material of this article.

## References

[1] R. F. Ziolo , S. Lipton , Z. Dori , J. Chem. Soc. D 1970, 1124–1125.

[2] N. Estrada-Ortiz , F. Guarra , I. A. M. de Graaf , L. Marchetti , M. H. de Jager , G. M. M. Groothuis , C. Gabbiani , A. Casini , ChemMedChem 2017, 12, 1429–1435. 28741878 10.1002/cmdc.201700316

[3] C. Zhang , C. Hemmert , H. Gornitzka , O. Cuvillier , M. Zhang , R. W.-Y. Sun , ChemMedChem 2018, 13, 1218–1229. 29603648 10.1002/cmdc.201800181

[4] M. T. Proetto , K. Alexander , M. Melaimi , G. Bertrand , N. C. Gianneschi , Chem. Eur. J. 2021, 27, 3772–3778. 33090571 10.1002/chem.202004317

[5] Z. Lei , M. Endo , H. Ube , T. Shiraogawa , P. Zhao , K. Nagata , X.-L. Pei , T. Eguchi , T. Kamachi , M. Ehara , T. Ozawa , M. Shionoya , Nat. Commun. 2022, 13, 4288. 35948553 10.1038/s41467-022-31891-3 PMC9365809

[6] R. Galassi , M. M. Ghimire , B. M. Otten , S. Ricci , R. N. McDougald , R. M. Almotawa , D. Alhmoud , J. F. Ivy , A.-M. M. Rawashdeh , V. N. Nesterov , E. W. Reinheimer , L. M. Daniels , A. Burini , M. A. Omary , PNAS 2017, 201700890. 10.1073/pnas.1700890114 PMC5495244 28615438

[7] C.-Y. Wong , S.-L. Lai , M.-Y. Leung , M.-C. Tang , L.-K. Li , M.-Y. Chan , V. W.-W. Yam , J. Am. Chem. Soc. 2023, 145, 2638–2646. 36633557 10.1021/jacs.2c12674

[8] M. Baron , C. Tubaro , A. Biffis , M. Basato , C. Graiff , A. Poater , L. Cavallo , N. Armaroli , G. Accorsi , Inorg. Chem. 2012, 51, 1778–1784. 22268766 10.1021/ic2020786

[9] T. P. Seifert , V. R. Naina , T. J. Feuerstein , N. D. Knöfel , P. W. Roesky , Nanoscale 2020, 12, 20065–20088. 33001101 10.1039/d0nr04748a

[10] V. R. Naina , F. Krätschmer , P. W. Roesky , Chem. Commun. 2022, 58, 5332–5346. 10.1039/d2cc01093c 35416815

[11] J. Carlos Lima , L. Rodríguez , Chem. Soc. Rev. 2011, 40, 5442–5456. 21769365 10.1039/c1cs15123a

[12] J. Ma , J. Schaab , S. Paul , S. R. Forrest , P. I. Djurovich , M. E. Thompson , J. Am. Chem. Soc. 2023, 145, 20097–20108. 37642694 10.1021/jacs.3c07743

[13] T. J. Feuerstein , T. P. Seifert , A. P. Jung , R. Müller , S. Lebedkin , M. M. Kappes , P. W. Roesky , Chem. Eur. J. 2020, 26, 16676–16682. 32520425 10.1002/chem.202002466 PMC7756867

[14] A. Ying , S. Gong , Chem. - Eur. J. 2023, 29, e202301885. 37431981 10.1002/chem.202301885

[15] V. W.-W. Yam , K. Kam-Wing Lo , K. Man-Chung Wong , J. Organomet. Chem. 1999, 578, 3–30.

[16] S. Bestgen , M. T. Gamer , S. Lebedkin , M. M. Kappes , P. W. Roesky , Chem. Eur. J. 2015, 21, 601–614. 25377172 10.1002/chem.201404985

[17] Q.-M. Wang , Y.-A. Lee , O. Crespo , J. Deaton , C. Tang , H. J. Gysling , M. Concepción Gimeno , C. Larraz , M. D. Villacampa , A. Laguna , R. Eisenberg , J. Am. Chem. Soc. 2004, 126, 9488–9489. 15291522 10.1021/ja048091d

[18] R. Visbal , I. Ospino , J. M. López-de-Luzuriaga , A. Laguna , M. C. Gimeno , J. Am. Chem. Soc. 2013, 135, 4712–4715. 23485100 10.1021/ja401523x

[19] L. Gao , D. S. Niedzwiecki , N. Deligonul , M. Zeller , A. D. Hunter , T. G. Gray , Chem. Eur. J. 2012, 18, 6316–6327. 22473678 10.1002/chem.201102502

[20] V. R. Naina , A. K. Singh , P. Rauthe , S. Lebedkin , M. T. Gamer , M. M. Kappes , A.-N. Unterreiner , P. W. Roesky , Chem. Eur. J. 2023, 29, e202300497. 36930531 10.1002/chem.202300497

[21] V. R. Naina, A. K. Singh, Shubham, F. Krätschmer, S. Lebedkin, M. M. Kappes, P. W. Roesky, *Dalton Trans*. **2023**, *52*, 12618-12622. 10.1039/d3dt02317f 37642577

[22] M. Ali , L. Dondaine , A. Adolle , C. Sampaio , F. Chotard , P. Richard , F. Denat , A. Bettaieb , P. Le Gendre , V. Laurens , C. Goze , C. Paul , E. Bodio , J. Med. Chem. 2015, 58, 4521–4528. 25973667 10.1021/acs.jmedchem.5b00480

[23] L. Dondaine , D. Escudero , M. Ali , P. Richard , F. Denat , A. Bettaieb , P. Le Gendre , C. Paul , D. Jacquemin , C. Goze , E. Bodio , Eur. J. Inorg. Chem. 2016, 545–553.

[24] A. Trommenschlager , F. Chotard , B. Bertrand , S. Amor , P. Richard , A. Bettaïeb , C. Paul , J.-L. Connat , P. Le Gendre , E. Bodio , ChemMedChem 2018, 13, 2408–2414. 30203922 10.1002/cmdc.201800474

[25] C. Sobrerroca , I. Angurell , A. de Aquino , G. Romo , C. Jubert , L. Rodríguez , ChemPlusChem 2023, 88, e202300020. 36800440 10.1002/cplu.202300020

[26] S. Balcıoğlu , M. Olgun Karataş , B. Ateş , B. Alıcı , İ. Özdemir , Bioorg. Med. Chem. Lett. 2020, 30, 126805. 31753700 10.1016/j.bmcl.2019.126805

[27] K. R. G. Lim , D. Darwan , H. Wijaya , Z. C. Lim , J. Shanmugam , T. Wang , L. J. Lim , W. H. Ang , Z.-K. Tan , Adv. Mater. Interfaces 2020, 7, 2000920.

[28] R. Hoffmann , Angew. Chem. Int. Ed. 1982, 21, 711–724.

[29] H.-Y. Chao , B.-C. Su , C.-L. Li , C.-K. Lam , X.-L. Feng , Inorg. Chem. Commun. 2011, 14, 1436–1439.

[30] J. Zheng , Z. Lu , K. Wu , G.-H. Ning , D. Li , Chem. Rev. 2020, 120, 9675–9742. 32786416 10.1021/acs.chemrev.0c00011

[31] J. C. Vickery , M. M. Olmstead , E. Y. Fung , A. L. Balch , Angew. Chem. Int. Ed. 1997, 36, 1179–1181.

[32] M. M. Ghimire , V. N. Nesterov , M. A. Omary , Inorg. Chem. 2017, 56, 12086–12089. 28956897 10.1021/acs.inorgchem.7b01679

[33] V. R. Naina, Dissertation, Karlsruhe Institute of Technology (Cuvillier Verlag, Göttingen), **2023**.

[34] TURBOMOLE V. 7.7 University of Karlsruhe and Forschungszentrum Karlsruhe GmbH 1989–2007. TURBOMOLE GmbH since 2007. Available from https://turbomole.org.

[35] Y. J. Franzke , C. Holzer , J. H. Andersen , T. Begušić , F. Bruder , S. Coriani , F. Della Sala , E. Fabiano , D. A. Fedotov , S. Fürst , S. Gillhuber , R. Grotjahn , M. Kaupp , M. Kehry , M. Krstić , F. Mack , S. Majumdar , B. D. Nguyen , S. M. Parker , F. Pauly , A. Pausch , E. Perlt , G. S. Phun , A. Rajabi , D. Rappoport , B. Samal , T. Schrader , M. Sharma , E. Tapavicza , R. S. Treß , V. Voora , A. Wodyński , J. M. Yu , B. Zerulla , F. Furche , C. Hättig , M. Sierka , D. P. Tew , F. Weigend , J. Chem. Theo. Comp. 2023, 19, 6859–6890. 10.1021/acs.jctc.3c00347 PMC10601488 37382508

[36] A. D. Laurent , D. Jacquemin , Int. J. Quantum Chem. 2013, 113, 2019–2039.

[37] R. S. Mulliken , Chem. Phys. 1955, 23, 1833–1840.

[38] A. S. Romanov , S. T. E. Jones , Q. Gu , P. J. Conaghan , B. H. Drummond , J. Feng , F. Chotard , L. Buizza , M. Foley , M. Linnolahti , D. Credgington , M. Bochmann , Chem. Sci. 2020, 11, 435–446. 32190264 10.1039/c9sc04589a PMC7067249

[39] D. Di , A. S. Romanov , L. Yang , J. M. Richter , J. P. H. Rivett , S. Jones , T. H. Thomas , M. Abdi Jalebi , R. H. Friend , M. Linnolahti , M. Bochmann , D. Credgington , Science 2017, 356, 159–163. 28360136 10.1126/science.aah4345

[40] R. Hamze , S. Shi , S. C. Kapper , D. S. Muthiah Ravinson , L. Estergreen , M.-C. Jung , A. C. Tadle , R. Haiges , P. I. Djurovich , J. L. Peltier , R. Jazzar , G. Bertrand , S. E. Bradforth , M. E. Thompson , J. Am. Chem. Soc. 2019, 141, 8616–8626. 31062972 10.1021/jacs.9b03657

[41] R. Hamze , M. Idris , D. S. Muthiah Ravinson , M. C. Jung , R. Haiges , P. I. Djurovich , M. E. Thompson , Front. Chem. 2020, 8, 401. 32457877 10.3389/fchem.2020.00401 PMC7225363

[42] J. Arcau , V. Andermark , E. Aguiló , A. Gandioso , A. Moro , M. Cetina , J. C. Lima , K. Rissanen , I. Ott , L. Rodríguez , Dalton Trans. 2014, 43, 4426–4436. 24302256 10.1039/c3dt52594e

[43] A. Pinto , C. Cunha , G. Aullón , J. C. Lima , L. Rodríguez , J. S. Seixas de Melo , J. Phys. Chem. B 2021, 125, 11751–11760. 34665627 10.1021/acs.jpcb.1c07985

[44] C. N. Muniz , J. Schaab , A. Razgoniaev , P. I. Djurovich , M. E. Thompson , J. Am. Chem. Soc. 2022, 144, 17916–17928. 36126274 10.1021/jacs.2c06948

[45] T.-y. Li , D. S. Muthiah Ravinson , R. Haiges , P. I. Djurovich , M. E. Thompson , J. Am. Chem. Soc. 2020, 142, 6158–6172. 32118418 10.1021/jacs.9b13755

[46] V. W.-W. Yam , T.-F. Lai , C.-M. Che , J. Chem. Soc. Dalton Trans. 1990, 3747–3752.

[47] M. A. Rauf , S. Hisaindee , J. Mol. Struct. 2013, 1042, 45–56.

[48] I. O. Koshevoy , Y.-C. Chang , A. J. Karttunen , M. Haukka , T. Pakkanen , P.-T. Chou , J. Am. Chem. Soc. 2012, 134, 6564–6567. 22469012 10.1021/ja3018994

[49] V. R. Naina , A. K. Singh , Shubham , J. Krämer , M. Iqbal , P. W. Roesky , Inorg. Chem. Front. 2024, 11, 6079–6088..

