# Supplementary material for: Dye Induced Luminescence Properties of Gold(I) Complexes with near Unity Quantum Efficiency
Source: Angew Chem Int Ed Engl. 2024 Oct 22;64(1):e202414517. doi: 10.1002/anie.202414517 (PMC11701351; doi:10.1002/anie.202414517)
Supplement: Supplementary file 5 — Supporting Information [file ANIE-64-e202414517-s004.pdf]

Angewandte Chemie
Eine Zeitschrift der Gesellschaft Deutscher Chemiker

Supporting Information

Dye Induced Luminescence Properties of Gold(I) Complexes with near Unity Quantum Efficiency

*V. R. Naina, S. Gillhuber, C. Ritschel, D. Jin, Shubham, S. Lebedkin, C. Feldmann, F. Weigend, M. M. Kappes, P. W. Roesky**

Supporting Information for

Dye induced luminescence properties of gold(I) complexes with near unity quantum efficiency

Vanitha R. Naina,[a] Sebastian Gillhuber,[a] Christian Ritschel,[a] Da Jin,[a] Shubham,[a] Sergei Lebedkin,[b] Claus Feldmann[a], Florian Weigend,[c] Manfred M. Kappes[b,d] and Peter W. Roesky*[a,b]

a. Institute of Inorganic Chemistry, Karlsruhe Institute of Technology, Engesserstraße 15, 76131, Karlsruhe, Germany.
b. Institute of Nanotechnology, Karlsruhe Institute of Technology, Hermann-von-Helmholtz-Platz 1, Eggenstein-Leopoldshafen, 76344, Karlsruhe, Germany.
c. Fachbereich Chemie, Philipps-Universität Marburg, Hans-Meerwein-Straße 4, 35032 Marburg, Germany
d. Institute of Physical Chemistry, Karlsruhe Institute of Technology, Fritz-Haber-Weg 2, 76131, Karlsruhe, Germany

*- corresponding author

Table of Contents

I. Synthesis and characterization S2

I.1 General procedures S2

II. NMR spectra S10

III. IR spectra S19

IV. Mass Spectra S22

V. X-ray crystallography S26

V.1 General methods S26

V.2. Table S1: Summary of crystal data S27

V.3 Crystal structures S29

VI. Photoluminescence data S32

VII. Quantum Chemical Calculations S34

VIII. References S60

I. Synthesis and characterization

I.1 General procedures

All air- and moisture-sensitive manipulations were performed under dry N_2 or Ar atmosphere using standard Schlenk techniques or in an argon-filled MBraun glovebox, unless otherwise stated. All solvents (Et_2O and *n*-pentane) were dried using an MBraun solvent purification system (SPS-800) and degassed. THF and DCM were distilled under nitrogen from potassium benzophenone ketyl and P_2O_5, respectively. THF-d_8 was dried over Na-K alloy. CD_2Cl_2 and $CDCl_3$ were dried over CaH_2. The deuterated solvents were degassed by freeze-pump-thaw cycles. [IPrAuOH],[1] [Au(PPh_3)Cl],[2] [(IPrAu)$_2$OH],[3] [*i*-PrNHCAu$_3$O],[4] [PPh$_3$Au$_3$O][5] and [PyPh$_2$Au$_3$O][6] were synthesized according to literature procedures. All other chemicals were obtained from commercial sources and used without further purification.

Elemental analyses were carried out with an Elementar vario MICRO cube.

NMR spectra were recorded on Bruker spectrometers (Avance Neo 300 MHz, Avance Neo 400 MHz or Avance III 400 MHz). Chemical shifts are referenced internally using signals of the residual protio solvent (^{1}H) or the solvent ($^{13}C\{^{1}H\}$) and are reported relative to tetramethylsilane (^{1}H, $^{13}C\{^{1}H\}$), H_3PO_4 (^{31}P). All NMR spectra were measured at 298 K, unless otherwise specified. The multiplicity of the signals is indicated as s = singlet, d = doublet, dd = doublet of doublets, t = triplet, q = quartet, m = multiplet and br = broad. Assignments were determined based on unambiguous chemical shifts, coupling patterns and ^{13}C-DEPT experiments.

Infrared (IR) spectra were recorded in the region 4000–400 cm^{-1} on a Bruker Tensor 37 FTIR spectrometer equipped with a room temperature DLaTGS detector, a diamond attenuated total reflection (ATR) unit and a nitrogen-flushed chamber. In terms of their intensity, the signals were classified into different categories (vs = very strong, s = strong, m = medium, w = weak, and sh = shoulder).

Synthesis of mononuclear NHC-coordinated gold complex [(Coum)AuIPr] (1)

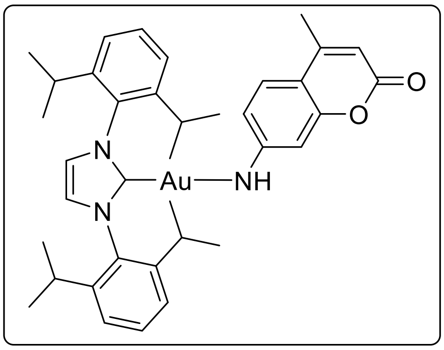

[IPrAuOH] (83.0 mg, 0.14 mmol, 1.0 eq) and coumarin (24.1 mg, 0.14 mmol, 1.0 eq), were dissolved in a vial containing 2 mL of THF. The reaction mixture was stirred overnight. On addition of *n*-pentane to the vial, yellow colored solid was obtained.

Analytical data for 1:

Yield: 83 mg (79 %). Anal. Calcd for $C_{37}H_{44}N_3O_2Au$ (759.74 g/mol): C, 58.49; H, 5.84; N, 5.53. Found: C, 58.30; H, 6.37; N, 5.41.

^{1}H NMR (400 MHz, THF-d_8): δ(ppm) = 7.58 (s, 2H, NHC-C*H*), 7.50 (t, *J* = 7.8 Hz, 2H, Ar-C*H*), 7.40-7.28 (m, 5H, Ar-C*H* and Coum-C*H*), 6.49 (d, *J* = 8.6 Hz, 1H, Coum-C*H*), 6.38 (s, 1H, Coum-C*H*), 5.81 (s, 1H, Coum-C*H*), 5.35 (s (br), 1H, N-*H*), 2.78-2.49 (m, 4H, -C*H*(CH_3)$_2$), 2.29 (s, 3H, Coum-CH_3), 1.35 (d, *J* = 6.9 Hz, 12H, -CH_3), 1.22 (d, *J* = 6.9 Hz, 12H, -CH_3).

^{13}C{^{1}H} NMR (101 MHz, THF-d_8): δ(ppm) = 177.4 (N*C*N), 161.0 (Coum-*C*O), 157.4 (Coum-*C*), 153.8 (Coum-*C*), 153.1 (Coum-*C*), 146.8 (Ar-*C*), 135.8 (Ar-*C*), 131.4 (Ar-*C*H), 126.5 (Coum-*C*H), 125.0 (Ar-*C*H), 124.7 (NHC-*C*H), 111.7 (Coum-*C*H), 111.0 (Coum-*C*), 110.0 (Coum-*C*H), 100.5 (Coum-*C*H), 29.8 (-*C*H(CH_3)$_2$), 24.3 (-*C*H_3), 18.5 (Coum-*C*H_3).

IR (ATR): $\tilde{\nu}$ (cm^{-1}) = 3439 (m), 3352 (m), 3246 (m), 2963 (s), 2927 (m), 2868 (m), 1686 (vs), 1629 (vs), 1548 (m), 1456 (m), 1399 (s), 1360 (m), 1332 (m), 1261 (m), 1214 (m), 1179 (m), 1157 (m), 1116 (m), 1037 (m), 1061 (m), 979 (m), 941 (m), 893 (m), 854 (m), 834 (m), 805(vs), 762 (s), 744 (s), 705 (m), 639 (m), 579 (m), 541 (m), 464 (sh), 447 (s).

Synthesis of mononuclear PPh_3-coordinated gold complex [(Coum)Au(PPh_3)$_2$]:

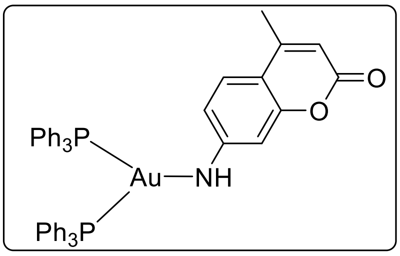

To a solution of [Au(PPh_3)Cl] (36.6 mg, 0.074 mmol) in dichloromethane (DCM) (10 ml) was added a solution of KNHCoumarin, which was prepared by reacting coumarin (13.0 mg, 0.074 mmol) with ^{t}BuOK (8.3 mg, 0.074 mmol) in methanol (10 ml). After stirring at room temperature for 3 hours, the solvent was evaporated. The residue was extracted with dichloromethane, and the filtrate was concentrated.

***In-situ* characterization data for [(Coum)Au(PPh_3)$_2$]:**

^{1}H NMR (400 MHz, CD_2Cl_2): δ(ppm) = 7.58-7.44 (m, 31H, Ph-C*H* and Coum-C*H*), 7.17 (d, *J* = 8.0 Hz, 1H, Coum-C*H*), 6.63-6.54 (s, 1H, Coum-C*H*, merged with unidentified product), 5.65 (s, 1H, Coum-C*H*), 4.99 (s, 1H, N*H*), 2.25 (s, 3H, Coum-C*H₃*).
^{31}P{^{1}H} NMR (162 MHz, 298 K, CD_2Cl_2): δ(ppm) = 32.7 (s).
^{13}C{^{1}H} NMR (101 MHz, CD_2Cl_2): δ(ppm) = 162.8 (Coum-*C*O), 162.3 (Coum-*C*), 156.7 (Coum-*C*), 153.2 (Coum-*C*), 134.2 (Ph-*C*H), 131.8 (Ph-*C*H), 129.7 (Ph-*C*), 129.1 (Ph-*C*H), 125.2 (Coum-*C*H), 113.7 (Coum-*C*H), 107.8 (Coum-*C*), 105.3 (Coum-*C*H), 99.8 (Coum-*C*H), 18.3 (Coum-*C*H_3).

The complex decomposes with time to yield red coloured crystals (during slow diffusion of diethylether into acetonitrile solution). The cell parameters obtained from single crystal X-ray diffraction analysis correspond to the reported Au_{11} cluster.

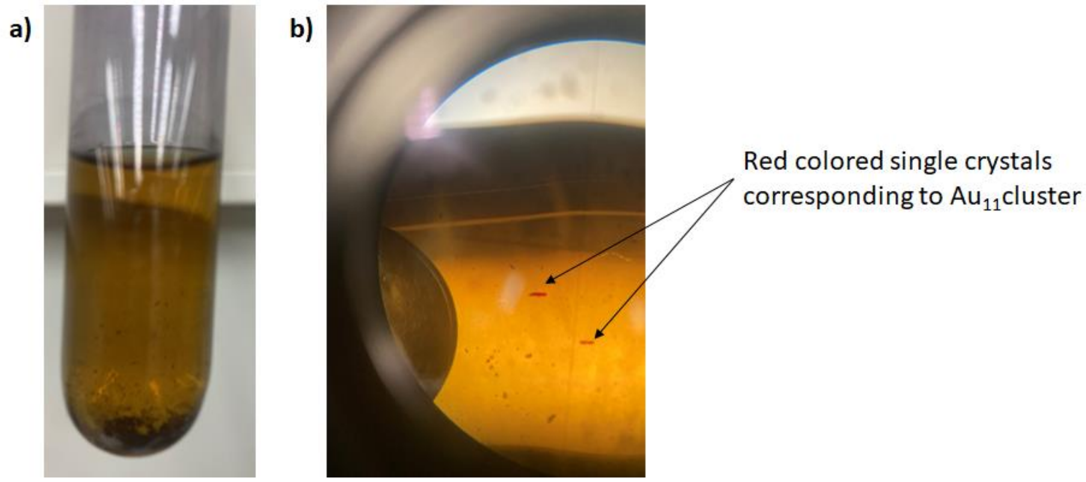

Figure S1. a) A picture of the decomposed reaction mixture; b) Microscopic image of the red crystals of Au_{11} formed during the crystallization procedure.

Synthesis of mononuclear *i*-PrNHC coordinated gold complex [(Coum)Au(PPh_3)$_2$]:

A similar procedure as that of [(Coum)Au(PPh_3)$_2$] (except *i*-PrNHCAuCl was used instead of PPh_3AuCl) was followed to attempt the isolation of mononuclear *i*-PrNHC coordinated gold complex. However, the reaction mixture turns black within 10 min and no reasonable ^{1}H NMR was obtained for the reaction mixture.

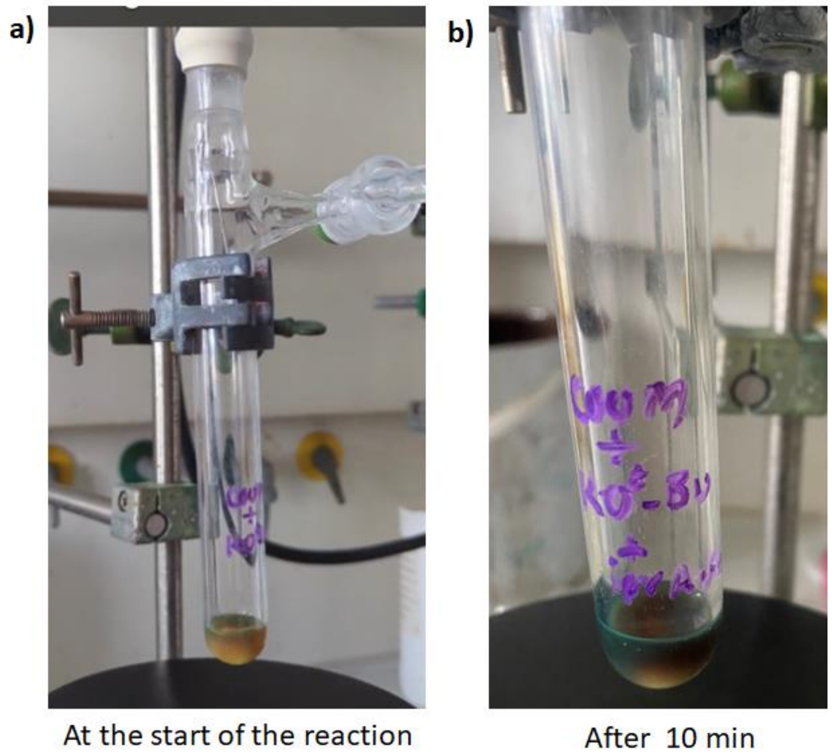

Figure S2. A picture of the reaction mixture a) at the beginning of the reaction; b) after 10 min.

Synthesis of dinuclear Dipp-coordinated gold complex [Coum(AuIPr)$_2$][BF$_4$] (2)

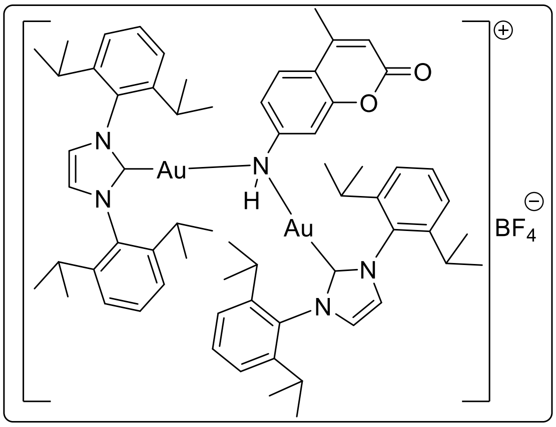

[(IPrAu)$_2$OH] (30.0 mg, 0.03 mmol, 1.0 eq) and coumarin (5.3 mg, 0.03 mmol, 1.0 eq), were dissolved in a Schlenk flask containing 2 mL of DCM in the presence of molecular sieves. The reaction mixture was stirred overnight. On addition of *n*-pentane to the flask, the desired product was isolated.

Analytical data for 2:

Yield: 26 mg (75 %) **HRMS (ESI)**: m/z Calcd for $[C_{64}H_{80}Au_2N_5O_2]^+$: 1344.5638 $[M-BF_4]^+$; found: 1344.5544.

^{1}H NMR (400 MHz, $CDCl_3$): δ (ppm) = 7.47 (t, *J* = 7.8 Hz, 4H, Ar-C*H*), 7.26 (d, *J* = 7.8 Hz, 4H, Ar-C*H*), 7.24 (s, 4H, NHC-C*H*), 7.08 (d, *J* = 7.8 Hz, 4H, Ar-C*H*), 7.00 (d, *J* = 8.6 Hz, 1H, Coum-C*H*), 6.16 (s, 1H, Coum-C*H*), 5.96 (d, *J* = 8.6 Hz, 1H, Coum-C*H*), 5.64 (s, 1H, Coum-C*H*), 3.41 (s, 1H, N*H*), 2.39 (s, 3H, Coum-CH_3), 2.39-2.21 (m, 8H, -C*H*(CH_3)$_2$), 1.17 (d, *J* = 6.8 Hz, 12H, -CH_3), 1.10 (dd, *J* = 6.8, 3.3 Hz, 24H, -CH_3), 0.84 (d, *J* = 6.8 Hz, 12H, -CH_3).

$^{13}C\{^1H\}$ NMR (101 MHz, $CDCl_3$): δ(ppm) = 171.0 (N*C*N), 161.0 (Coum-*C*O), 154.9 (Coum-*C*), 153.7 (Coum-*C*), 152.4 (Coum-*C*), 145.3 (Ar-*C*), 133.6 (Ar-*C*), 130.8 (Ar-*C*H), 125.3 (Coum-*C*H), 124.3 (Ar-CH), 124.1 (NHC-*C*H), 116.9 (Coum-*C*H), 114.3 (Coum-*C*H), 112.2 (Coum-*C*H), 108.4 (Coum-*C*H), 28.7 (-*C*H(CH_3)$_2$), 28.6 (-*C*H(CH_3)$_2$), 24.7 (-*C*H_3), 24.0 (-*C*H_3), 23.9 (-*C*H_3), 24.0 (-*C*H_3), 18.7 (Coum-*C*H_3).

IR (ATR): $\tilde{\nu}$ (cm^{-1}) = 3171 (m), 3145 (w), 3073 (m), 2961 (vs), 2927 (s), 2869 (s), 1719 (s), 1653 (w), 1645 (w), 1594 (s), 1559 (m), 1536 (w), 1455 (sh), 1436 (s), 1419 (sh), 1385 (s), 1363 (s), 1330 (m), 1257 (m), 1215 (m), 1183 (m), 1139 (m), 1088 (s), 1054 (vs), 998 (m), 979 (m), 947 (m), 850 (w), 803 (s), 756 (s), 707 (m), 692 (m), 641 (w), 540 (m), 518 (m), 453 (m).

Synthesis of trinuclear *i*-PrNHC gold complex [Coum{Au(*i*-PrNHC)}$_3$][BF_4] (3)

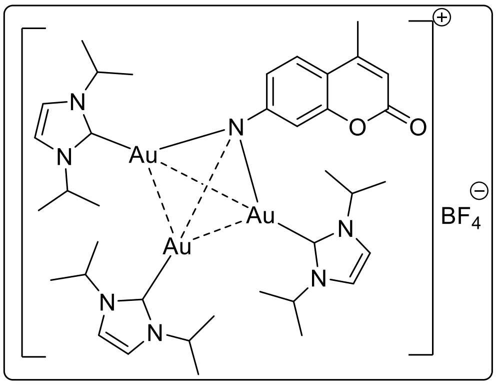

To a vial containing [(*i*-PrNHC)$_3$$Au_3O$] cluster (110 mg, 1.0 eq) and coumarin (20 mg, 1.0 eq), 2 mL of DCM were added. After stirring for 4 h at rt, the reaction mixture was filtered and concentrated. Diffusion of diethylether into a DCM solution led to the isolation of single crystals.

<u>Analytical data for 3:</u>

Yield: 105 mg (73 %). Anal. Calcd for $C_{37}H_{55}N_7O_2Au_3BF_4$ (1307.60 g/mol): C, 33.99; H, 4.24; N, 7.50. Found: C, 33.43; H, 4.32; N, 7.44.

^1H NMR (400 MHz, CD_2Cl_2): δ(ppm) = 7.56-7.37 (m, 2H, Coum-C*H*), 7.31 (d, *J* = 8.6 Hz, 1H, Coum-C*H*), 7.08 (s, 6H, NHC-C*H*), 5.93 (s, 1H, Coum-C*H*), 4.98 (sept, *J* = 6.8 Hz, 6H, NHC-C*H*(CH_3)$_2$), 2.34 (d, *J* = 1.2 Hz, 3H, Coum-C*H*$_3$), 1.50 (d, *J* = 6.8 Hz, 36H, (NHC-CH(C*H*$_3$)$_2$)).

$^{13}C\{^1H\}$ NMR (101 MHz, CD_2Cl_2): δ(ppm) = 170.2 (N*C*N), 168.5 (Coum-*C*O), 162.0 (Coum-*C*), 154.7 (Coum-*C*), 153.1 (Coum-*C*), 123.9 (Coum-*C*H), 122.8 (Coum-*C*H), 117.5 (NHC-*C*H), 113.3 (Coum-*C*), 111.6 (Coum-*C*H), 110.2 (Coum-*C*H), 53.9 (NHC-*C*H(CH_3)$_2$) (signal is covered by CD_2Cl_2 signal), 23.5 (NHC-CH(*C*H_3)$_2$), 18.8 (Coum-*C*H_3).

HRMS (ESI): m/z Calcd for $[C_{37}H_{55}Au_3N_7O_2]^+$: 1220.3408 $[M-BF_4]^+$; found: 1220.3373.

IR (ATR): $\tilde{\nu}$ (cm^{-1}) = 3159 (w), 3132 (w), 3092 (w), 2972 (s), 2933 (m), 2876 (w), 1719 (vs), 1695 (sh), 1590 (vs), 1559 (m), 1530 (m), 1490 (w), 1466 (m), 1431 (m), 1417 (m), 1387 (s), 1368 (s), 1322 (w), 1304 (w), 1282 (w), 1257 (s), 1210 (s), 1179 (m), 1135 (s), 1048 (vs), 978 (m), 882 (w), 847 (m), 815 (w), 739 (m), 692 (m), 669 (w), 628 (w), 574 (w), 513 (m), 450 (w), 437 (w).

Synthesis of trinuclear PPh_3 gold complex [Coum($AuPPh_3$)$_3$][BF_4] (4)

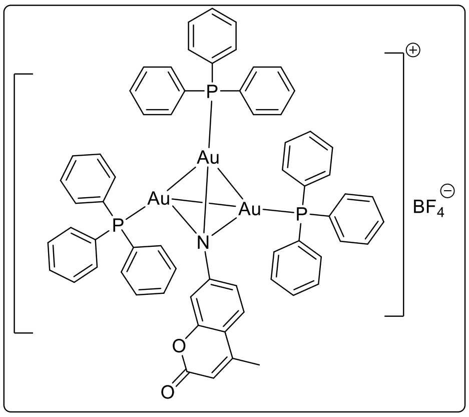

To a Schlenk flask containing [PPh_3Au_3O] cluster (14.8 mg, 1.0 eq) and coumarin (3.5 mg, 2.0 eq), 2 mL of DCM was added. After stirring overnight at rt, the reaction mixture was filtered and dried. Diffusion of diethylether into a THF solution of cluster **18** led to the isolation of single crystals.

<u>Analytical data for 4:</u>

Yield: 10 mg (61 %). **HRMS (ESI)**: m/z Calcd for [$C_{64}H_{52}Au_3NO_2P_3$]$^+$: 1550.2202 [M-BF_4]$^+$; found: 1550.2206.

^{1}H NMR (400 MHz, 298 K, CD_2Cl_2): δ(ppm) 7.54-7.38 (m, 30H, Ph-C*H*), 7.33-7.23 (m, 18H, Ph-C*H* and Coum-C*H*), 6.00 (s, 1H, Coum-C*H*), 2.35 (s, 3H, Coum-C*H*$_3$).

^{31}P{^{1}H} NMR (162 MHz, 298 K, CD_2Cl_2): δ(ppm) = 28.3 (s).

^{13}C{^{1}H} NMR (100 MHz, 298 K, CD_2Cl_2): δ(ppm) = 161.5 (Coum-*C*O), 154.6 (Coum-*C*), 152.9 (Ph-*C*), 134.4 (Ph-*C*H), 132.5 (Ph-*C*H), 129.7 (Ph-*C*H), 129.3 (Coum-*C*H), 128.7 (Coum-*C*H), 124.7 (Coum-*C*), 122.3 (Coum-*C*), 114.8 (Coum-*C*), 111.9 (Coum-*C*H), 111.5 (Coum-*C*H), 18.8 (Coum-*C*H$_3$).

IR (ATR): $\tilde{\nu}$ (cm^{-1}) = 3072 (w), 3056 (w), 3010 (w), 2973 (w), 2925 (w), 2854 (w), 1893 (w), 1814 (w), 1771 (w), 1719 (s), 1635 (w), 1613 (sh), 1593 (vs), 1535 (w), 1480 (m), 1436 (s), 1414 (m), 1385 (m), 1364 (m), 1331 (w), 1313 (m), 1285 (w), 1258 (s), 1219 (w), 1184 (m), 1139 (s), 1099 (s), 1052 (s), 996 (m), 930 (m), 885 (w), 850 (w), 816 (m), 749 (s), 710 (m), 691 (s), 643 (w), 618 (w), 564 (w), 538 (s), 498 (s), 433 (m).

Synthesis of trinuclear $PyPh_2$ gold complex [Coum($AuPyPPh_2$)$_3$][BF_4] (5)

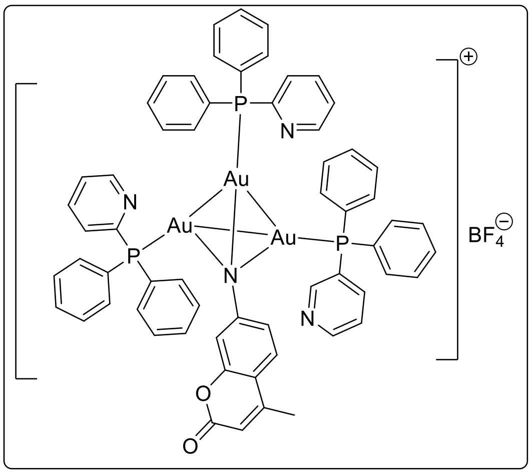

[$PyPh_2Au_3O$] cluster (14.8 mg, 1.0 eq) and coumarin (3.5 mg, 2.0 eq), were dissolved in 2 mL of DCM and stirred overnight. The cluster **5** was obtained as crystals on addition of diethylether to a conc. solution.

<u>Analytical data for 5:</u>

Yield: 18 mg (65 %). **HRMS (ESI)**: m/z Calcd for [$C_{61}H_{49}Au_3N_4O_2P_3$]$^+$: 1553.2059 [M-$BF_4$]$^+$; found: 1553.1960.

^{1}**H NMR** (400 MHz, CD_2Cl_2): δ(ppm) = 8.61 (d, *J* = 5.0 Hz, 3H, Py-C*H*), 7.76-7.52 (m, 21H, Ar-C*H*), 7.50-7.38 (m, 7H, Ar-C*H* + Coum-C*H*), 7.37-7.17 (m, 14H, Ar-C*H* + Coum-C*H*), 6.00 (s, 1H, Coum-C*H*), 2.35 (s, 3H, Coum-CH_3).

^{31}**P{^{1}H} NMR** (162 MHz, 298 K, CD_2Cl_2): δ(ppm) = 27.4 (s).

^{13}**C{^{1}H} NMR** (101 MHz, CD_2Cl_2): δ(ppm) = 165.2 (Py-*C*), 161.5 (Coum-*C*O), 154.6 (Coum-*C*), 153.8 (Coum-*C*), 152.9 (Coum-*C*), 151.8 (Ar-*C*H), 137.2 (Ar-*C*H), 134.7 (Ar-*C*H), 132.6 (Ar-*C*H), 130.9 (Ar-*C*H), 129.6 (Ar-*C*H), 129.0 (Ar-*C*), 125.9 (Coum-*C*H), 124.83 (Coum-*C*H), 122.43 (Ar-*C*H), 114.8 (Coum-*C*), 111.93 (Coum-*C*H), 111.53 (Coum-*C*H), 18.83 (Coum-*C*H_3).

IR (ATR): $\tilde{\nu}$ (cm^{-1}) = 3053 (w), 2165 (w), 1718 (m), 1700 (m), 1594 (s), 1570 (m), 1537 (w), 1480 (m), 1435 (m), 1422 (m), 1386 (m), 1366 (m), 1313 (w), 1260 (m), 1185 (m), 1139 (m), 1099 (m), 1050 (vs), 984 (m), 953 (w), 847 (m), 817 (w), 770 (m), 744 (s), 728 (m), 690 (vs), 617 (m), 580 (w), 539 (vs), 497 (vs), 436 (m).

Synthesis of mononuclear NHC-coordinated copper(I) complex [(Coum)CuIPr] (6)

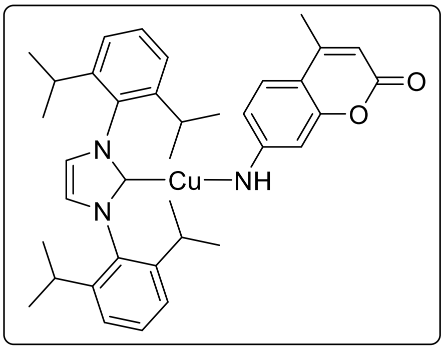

[IPrCuOH] (71.0 mg, 0.15 mmol, 1.0 eq) and coumarin (26.5 mg, 0.15 mmol, 1.0 eq), were suspended in a NMR containing 0.5 mL of C_6D_6. The reaction mixture was stirred overnight at 60 °C. The reaction mixture was filtered and the volatiles were removed. The desired product was extracted with THF. Yellow colored single crystals were obtained by layering the THF solution with n-pentane.

<u>Analytical data for 6:</u>

Yield: 14 mg (70 %).

^{1}**H NMR** (400 MHz, C_6D_6): δ(ppm) = 8.38 (s, 2H, NHC-C*H*), 8.13 – 7.84 (m, 7H, Ar-C*H* and Coum-C*H*), 7.77 (d, *J* = 7.7 Hz, 1H, Coum-C*H*), 7.53 (d, *J* = 8.6 Hz, 1H, Coum-C*H*), 6.50 (s, 1H, Coum-C*H*), 4.36 (s, 1H, N-*H*), 3.43-3.23 (m, 4H, -C*H*(CH_3)$_2$), 2.49 (s, 3H, Coum-CH_3), 2.14 (d, *J* = 6.9 Hz, 12H, -CH_3), 1.88 (d, *J* = 6.9 Hz, 12H, -CH_3).

^{13}**C{^{1}H} NMR** (101 MHz, C_6D_6): δ(ppm) = 182.5 (N*C*N), 165.1 (Coum-*C*O), 162.8 (Coum-*C*), 158.0 (Coum-*C*), 152.2 (Coum-*C*), 145.8 (Ar-*C*), 134.8 (Ar-*C*), 131.7 (Ar-*C*H), 130.6 (Coum-*C*H), 124.8 (Ar-*C*H), 122.8 (NHC-*C*H), 114.4 (Coum-*C*H), 106.6 (Coum-*C*), 104.8 (Coum-*C*H), 101.0 (Coum-*C*H), 29.0 (-*C*H(CH_3)$_2$), 25.0 (-*C*H_3), 23.8 (-*C*H_3), 18.0 (Coum-*C*H_3).

IR (ATR): $\tilde{\nu}$ (cm^{-1}) = 3355 (m), 3113 (w), 3073 (w), 2960 (s), 2926 (m), 2867 (m), 1696 (s), 1604 (s), 1577 (vs), 1524 (m), 1513 (m), 1459 (m), 1397 (vs), 1365 (m), 1342 (m), 1256 (m), 1204 (m), 1181 (w), 1141 (m), 1116 (m), 1060 (m), 973 (w), 937 (w), 881 (w), 803 (m), 756 (m), 699 (m), 645 (w), 605 (w), 578 (w), 548 (w), 515 (w), 497 (m), 448 (m).

II. NMR spectra

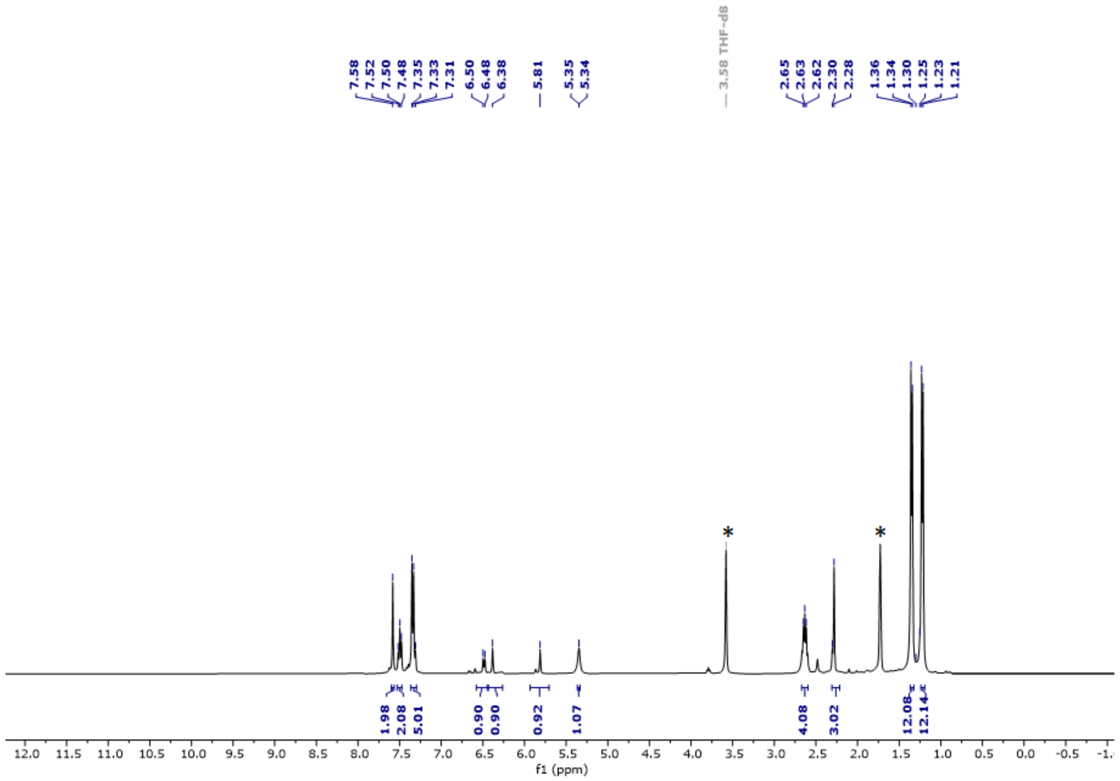

Figure S3. ^{1}H NMR spectrum of **1** in THF-d_8 at room temperature. *, residual protio solvent signal.

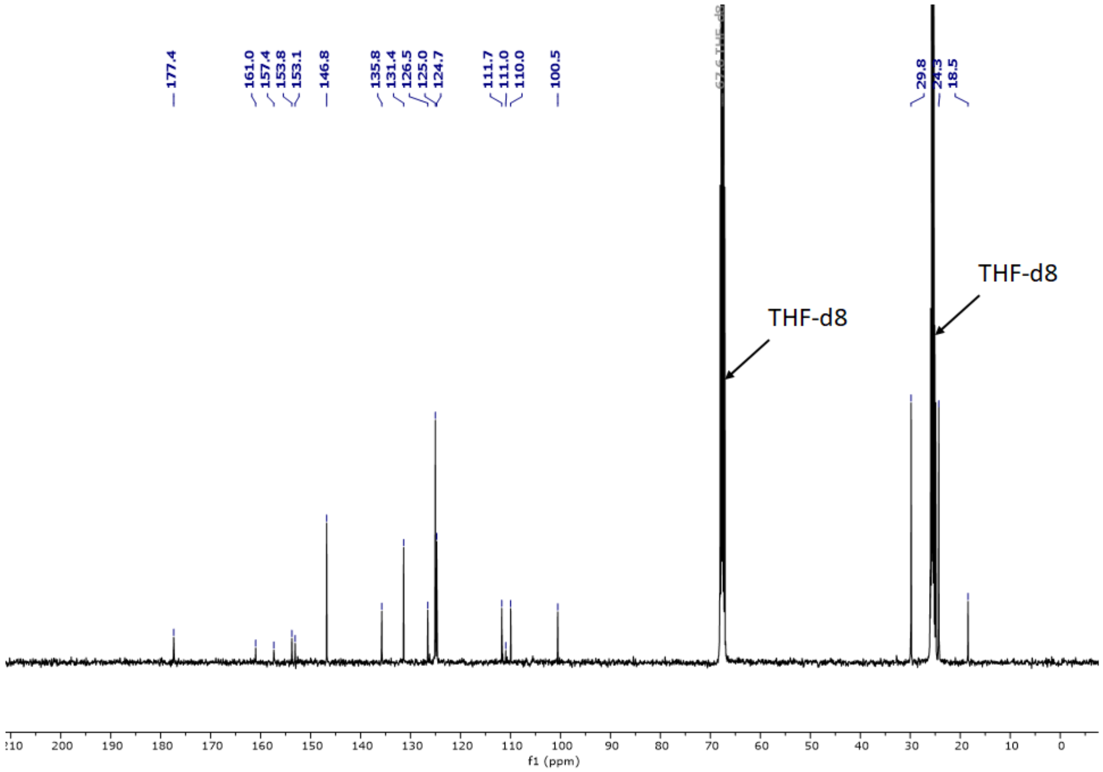

Figure S4. $^{13}C\{^{1}H\}$ NMR spectrum of **1** in THF-d_8 at room temperature.

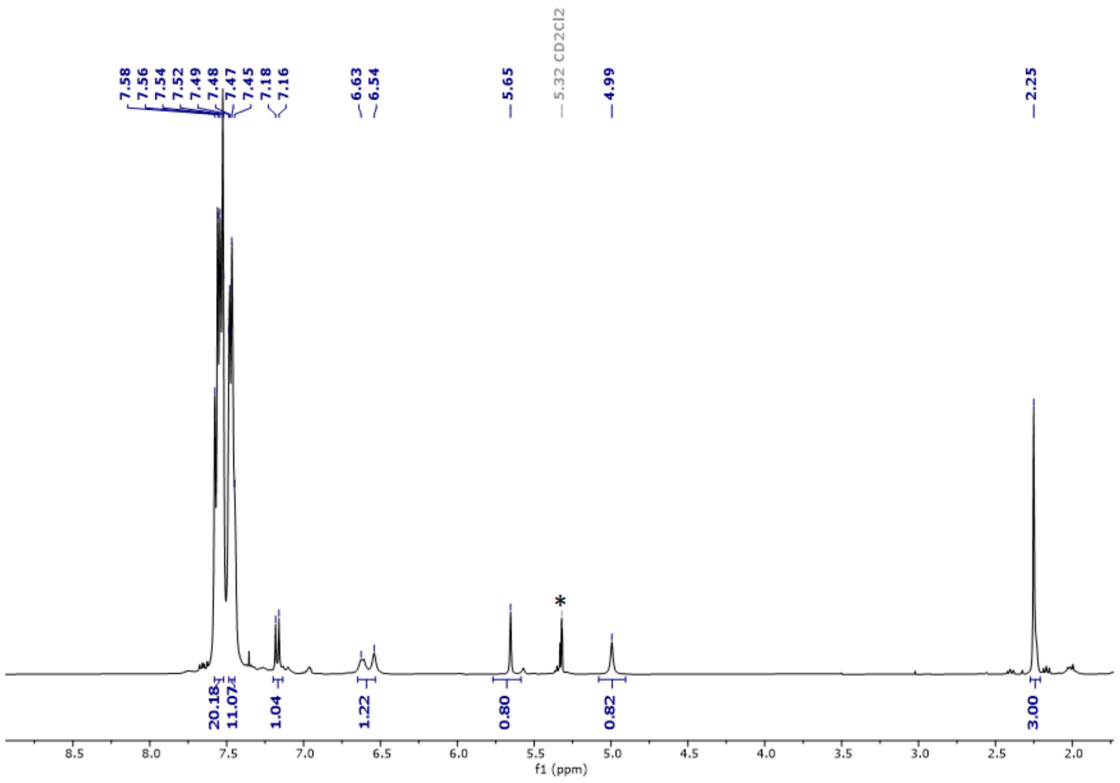

Figure S5. ^1H NMR spectrum of $[(Coum)Au(PPh_3)_2]$ in CD_2Cl_2 at room temperature (*in situ* reaction mixture). *, residual protio solvent signal.

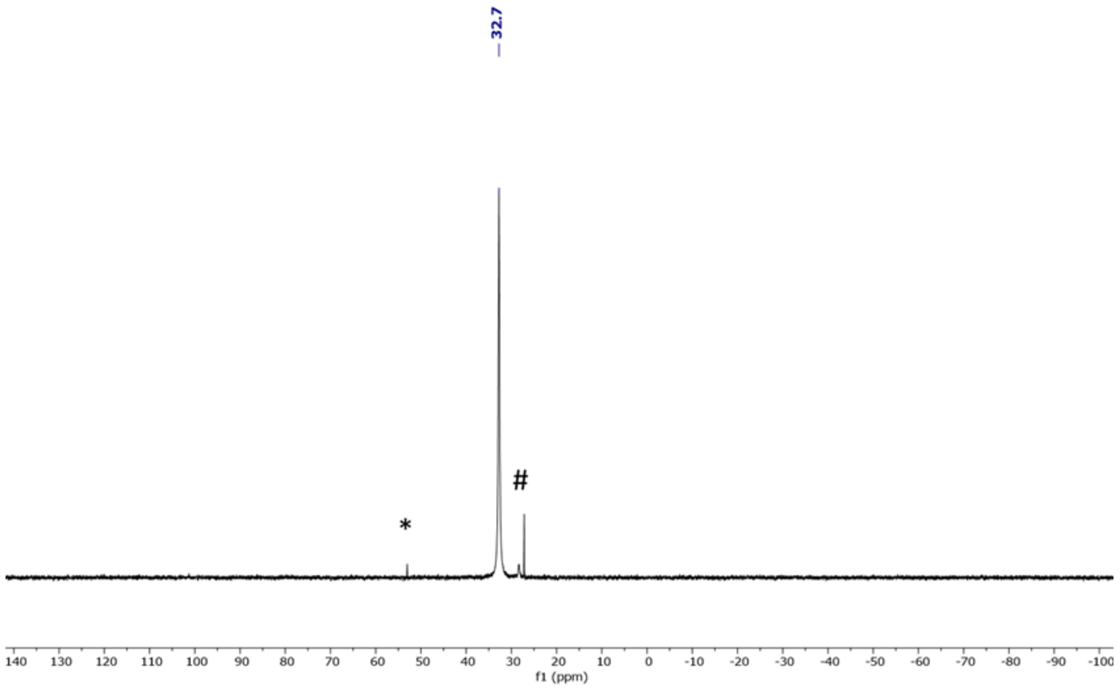

Figure S6. $^{31}P\{^1H\}$ NMR spectrum of $[(Coum)Au(PPh_3)_2]$ in CD_2Cl_2 at room temperature (*in situ* reaction mixture). *, Au_{11} cluster; #, unidentified minor products.

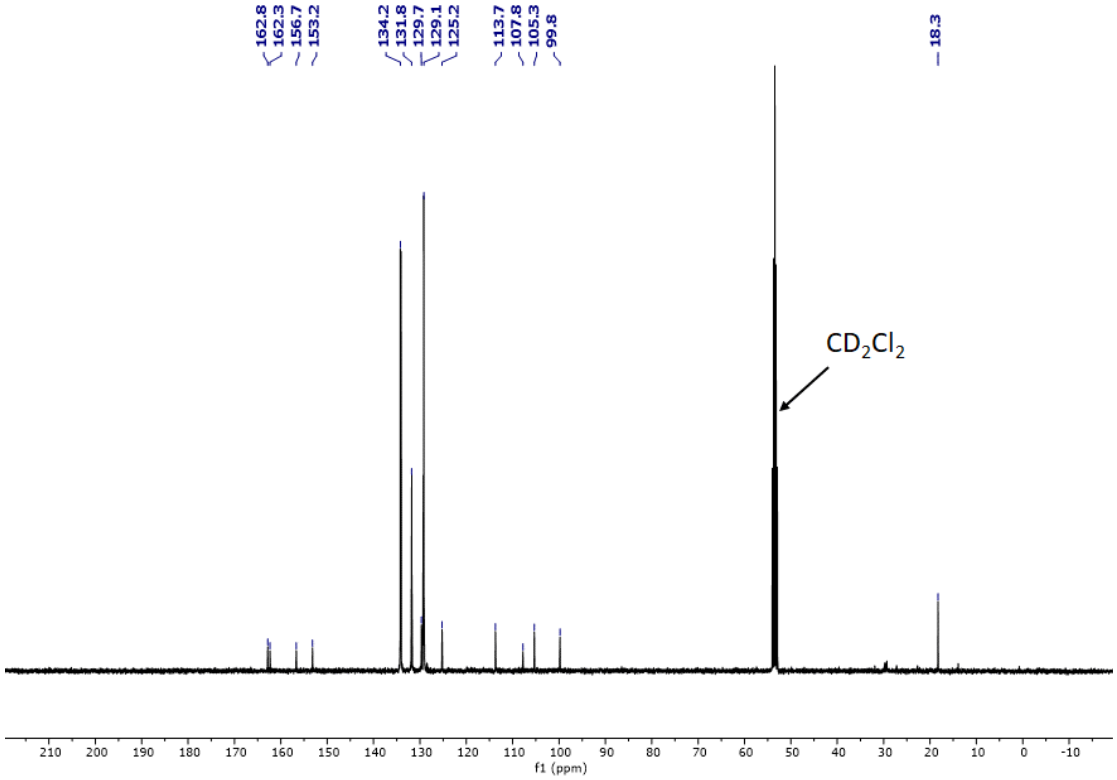

Figure S7. $^{13}C\{^{1}H\}$ NMR spectrum of $[(Coum)Au(PPh_3)_2]$ in CD_2Cl_2 at room temperature (*in situ* reaction mixture).

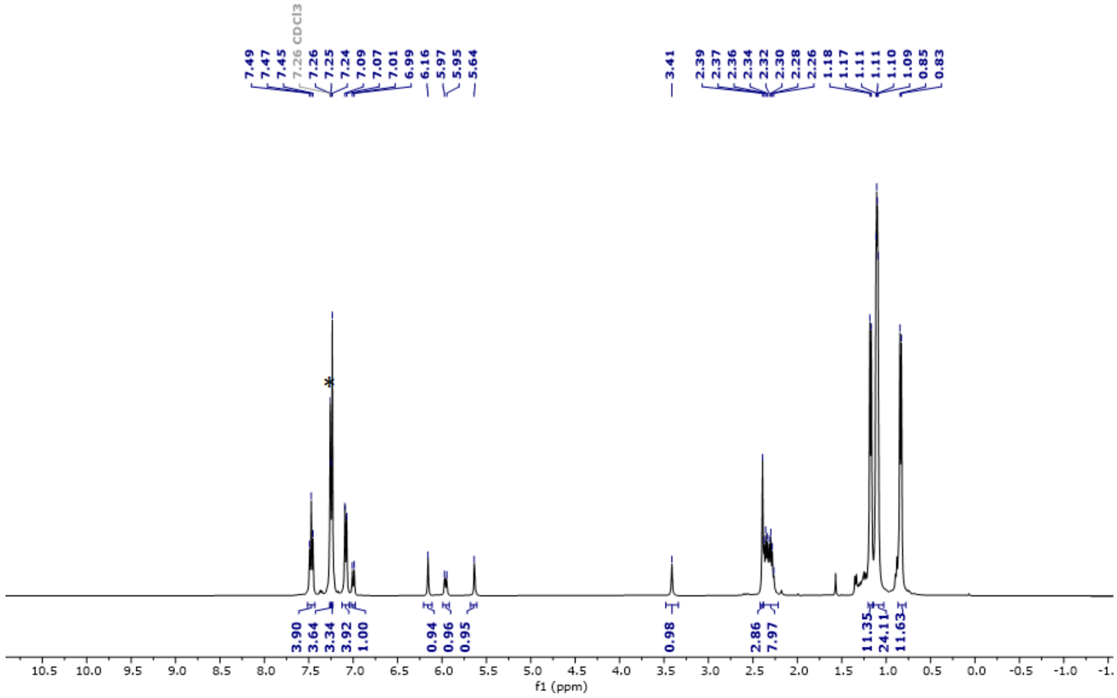

Figure S8. ^{1}H NMR spectrum of **2** in $CDCl_3$ at room temperature. *, residual protio solvent signal.

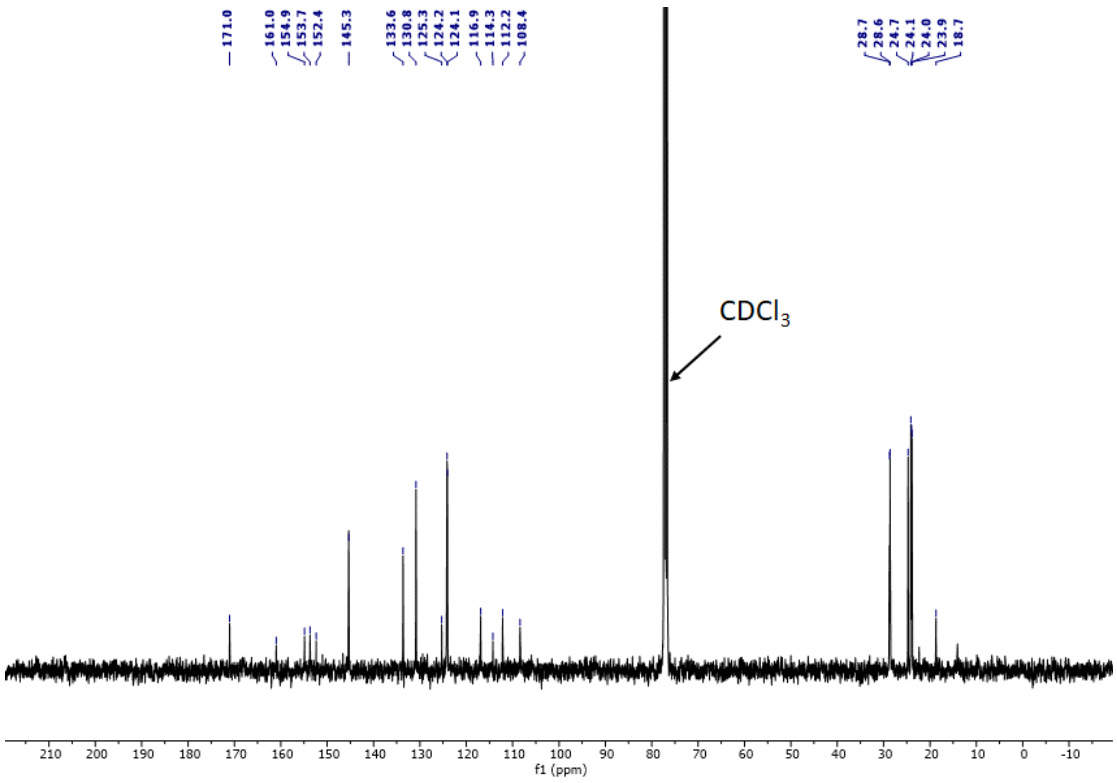

Figure S9. $^{13}C\{^1H\}$ NMR spectrum of **2** in $CDCl_3$ at room temperature.

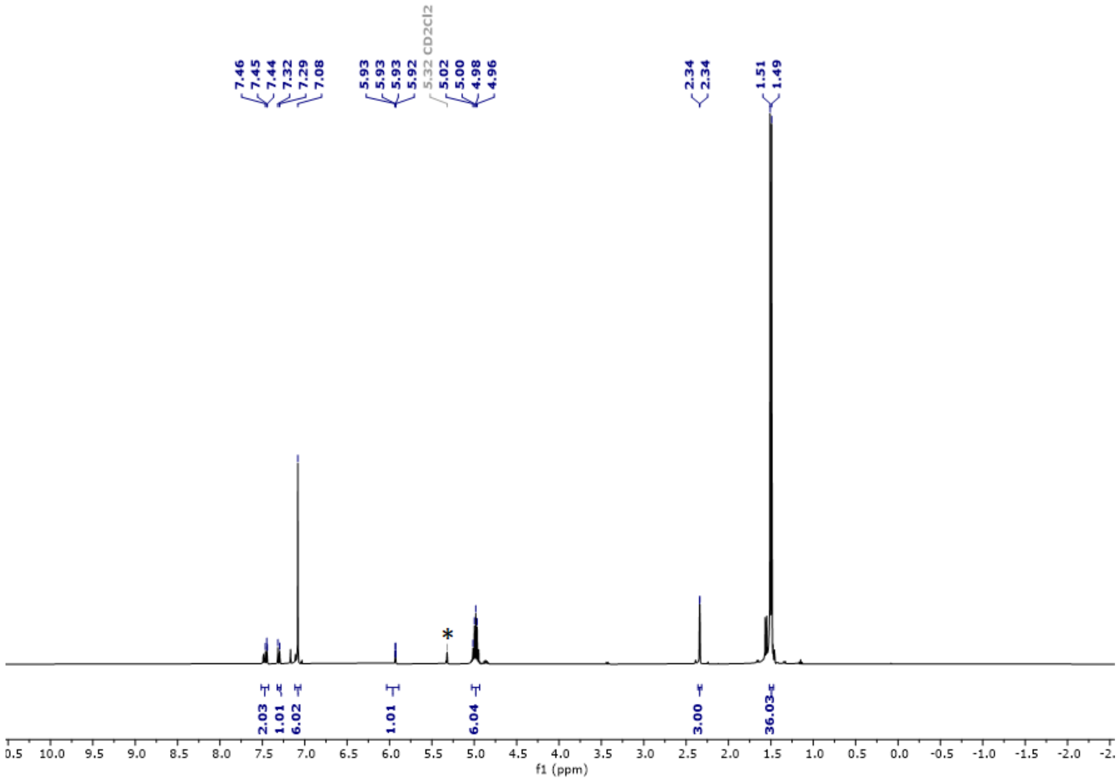

Figure S10. ^1H NMR spectrum of **3** in CD_2Cl_2 at room temperature. *, residual protio solvent signal.

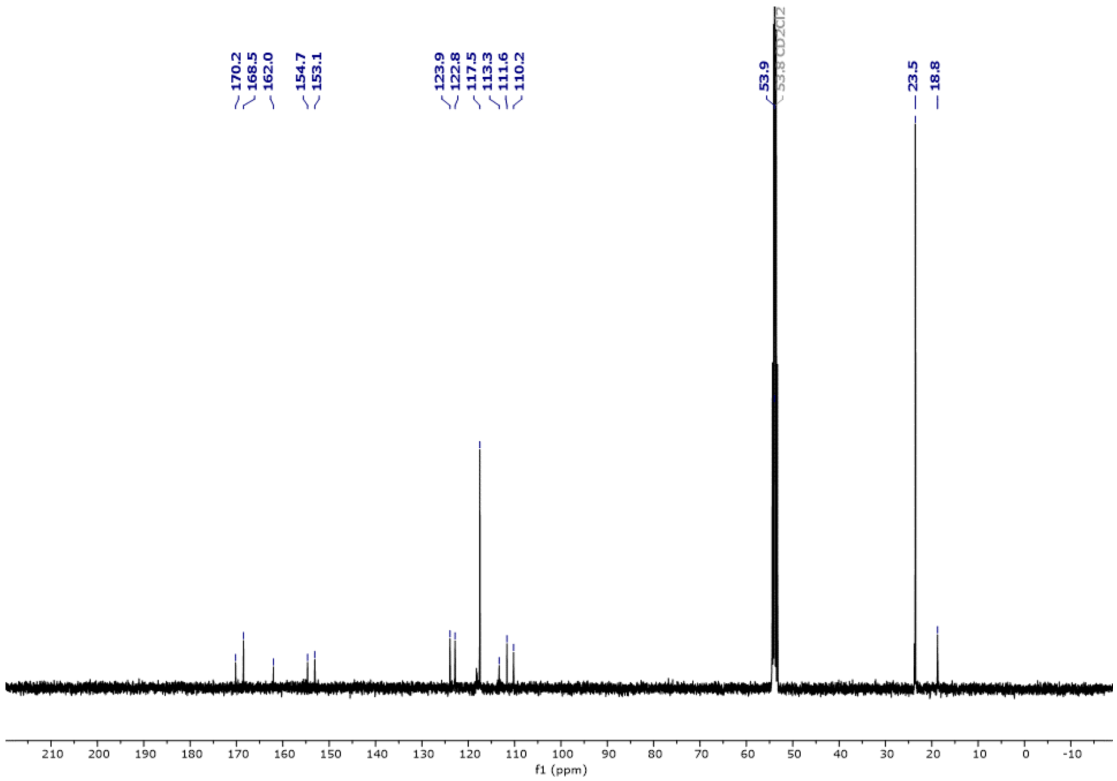

Figure S11. $^{13}C\{^1H\}$ NMR spectrum of **3** in CD_2Cl_2 at room temperature.

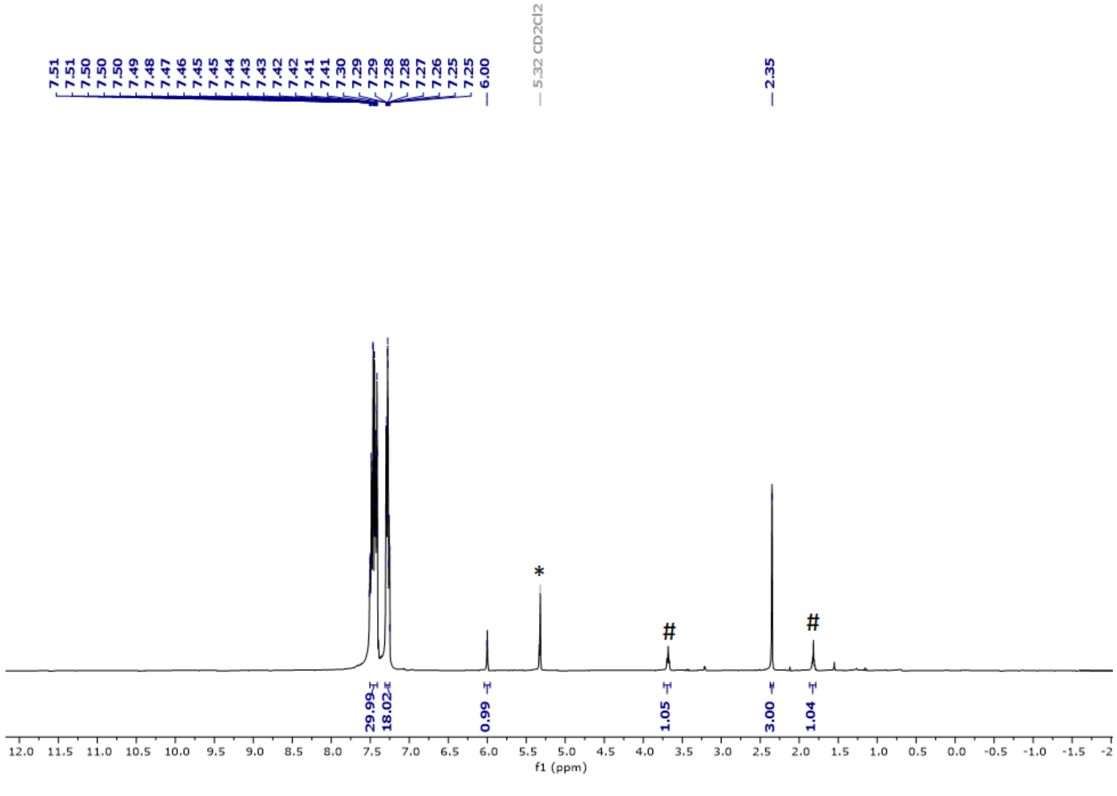

Figure S12. ^1H NMR spectrum of **4** in CD_2Cl_2 at room temperature. *, residual protio solvent signal. #- THF.

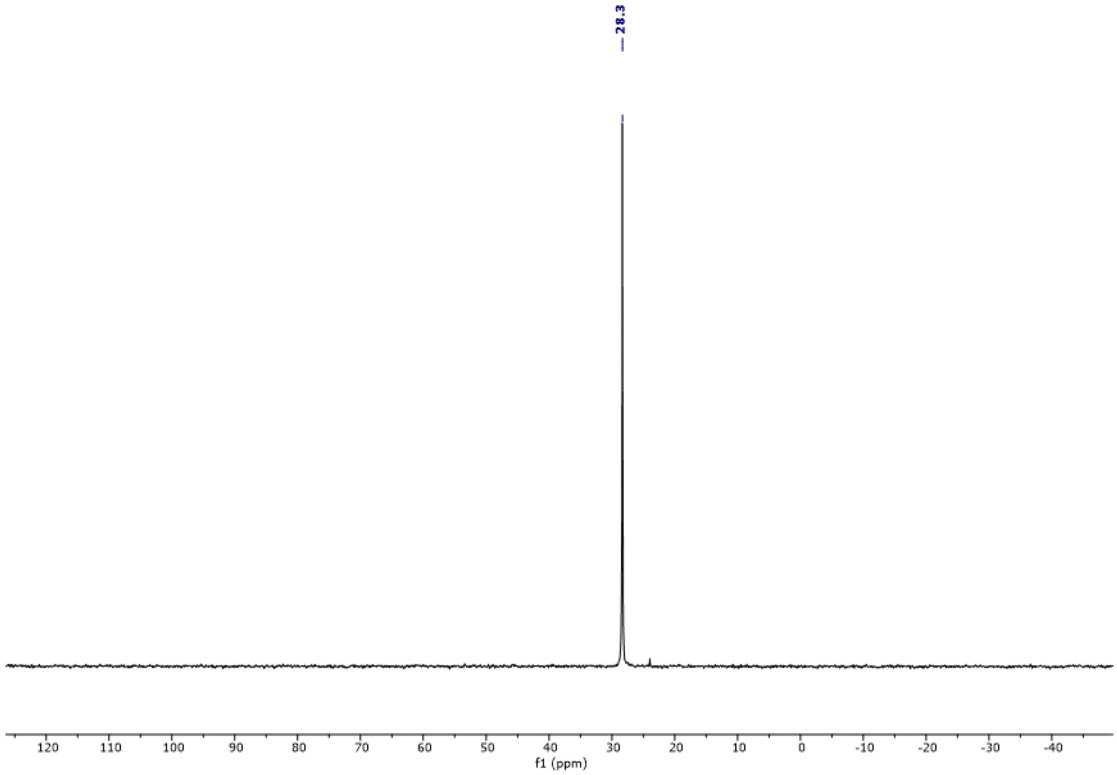

Figure S13. $^{31}P\{^{1}H\}$ NMR spectrum of **4** in CD_2Cl_2 at room temperature.

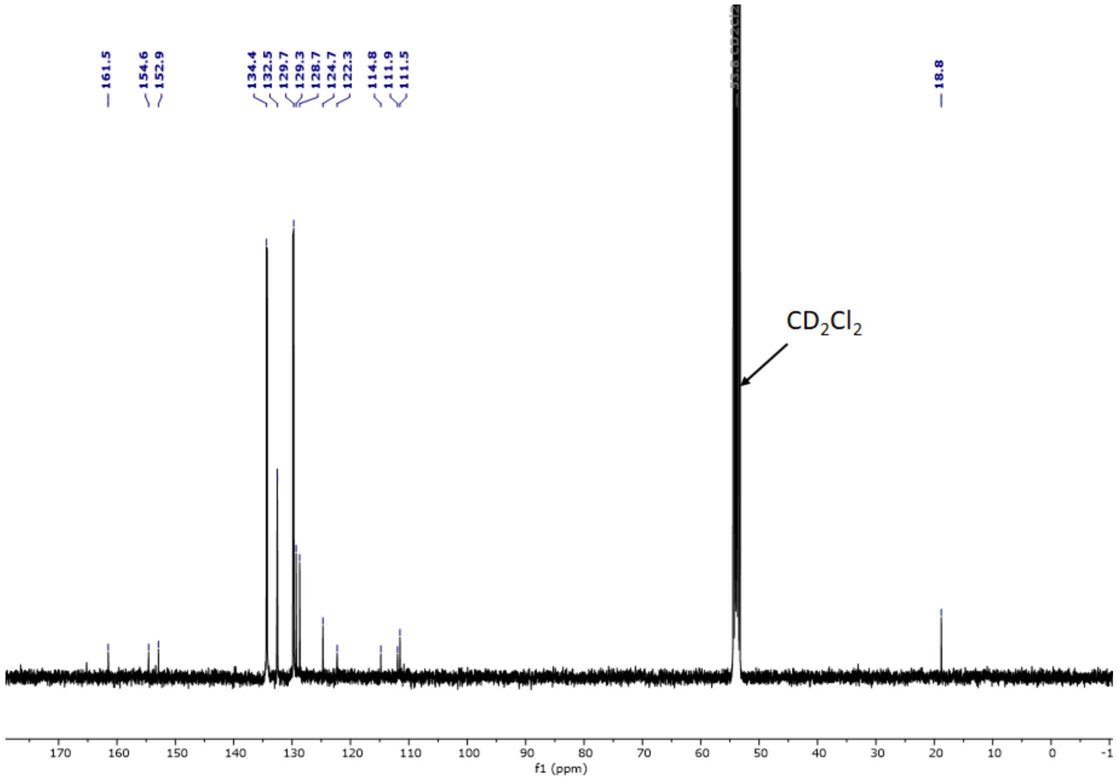

Figure S14. $^{13}C\{^{1}H\}$ NMR spectrum of **4** in CD_2Cl_2 at room temperature.

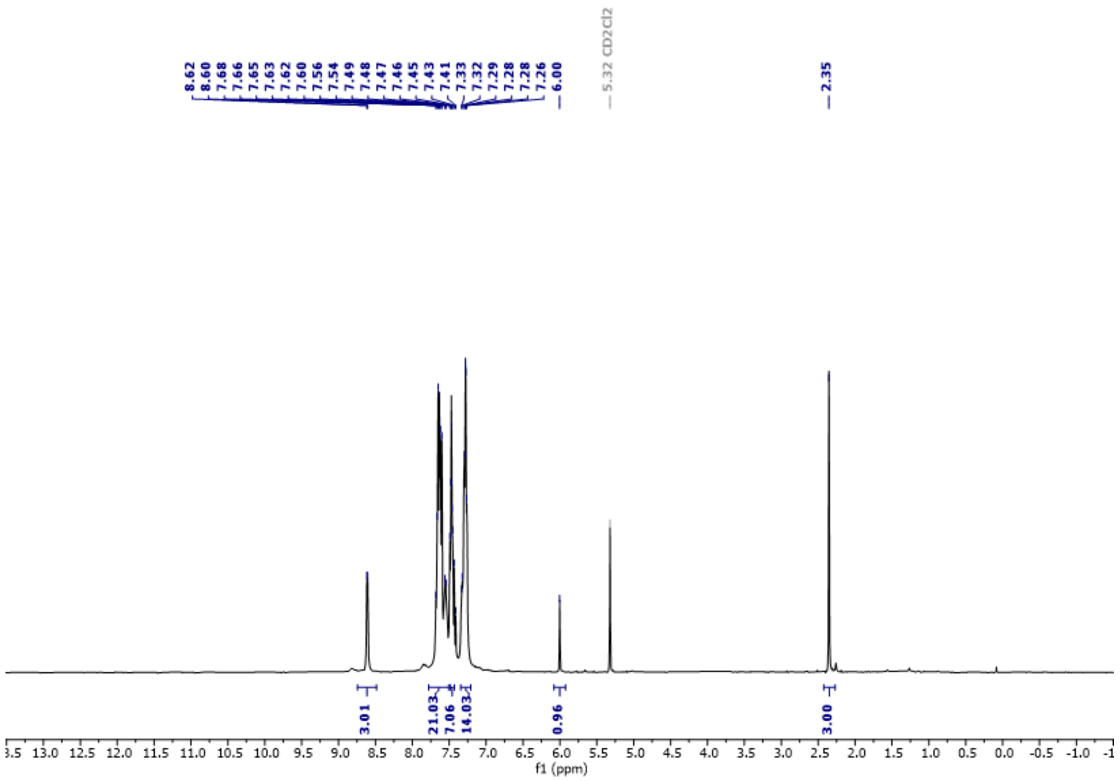

Figure S15. ^{1}H NMR spectrum of **5** in CD_2Cl_2 at room temperature. *, residual protio solvent signal.

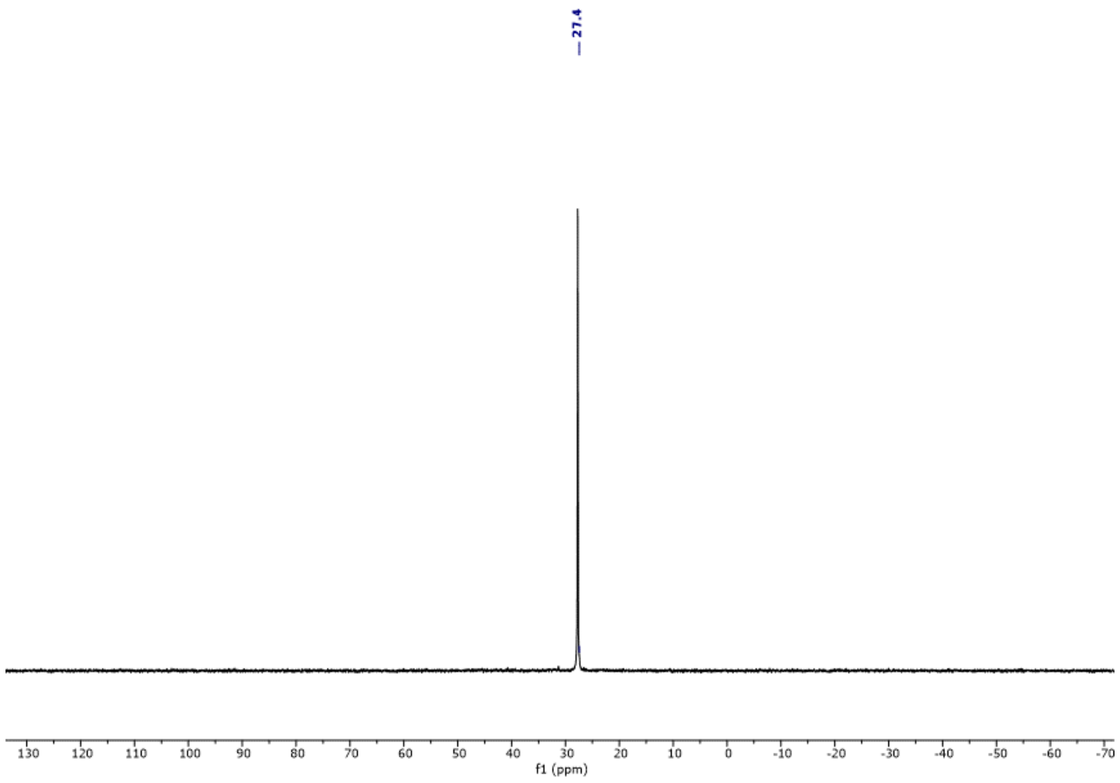

Figure S16. $^{31}P\{^1H\}$ NMR spectrum of **5** in CD_2Cl_2 at room temperature.

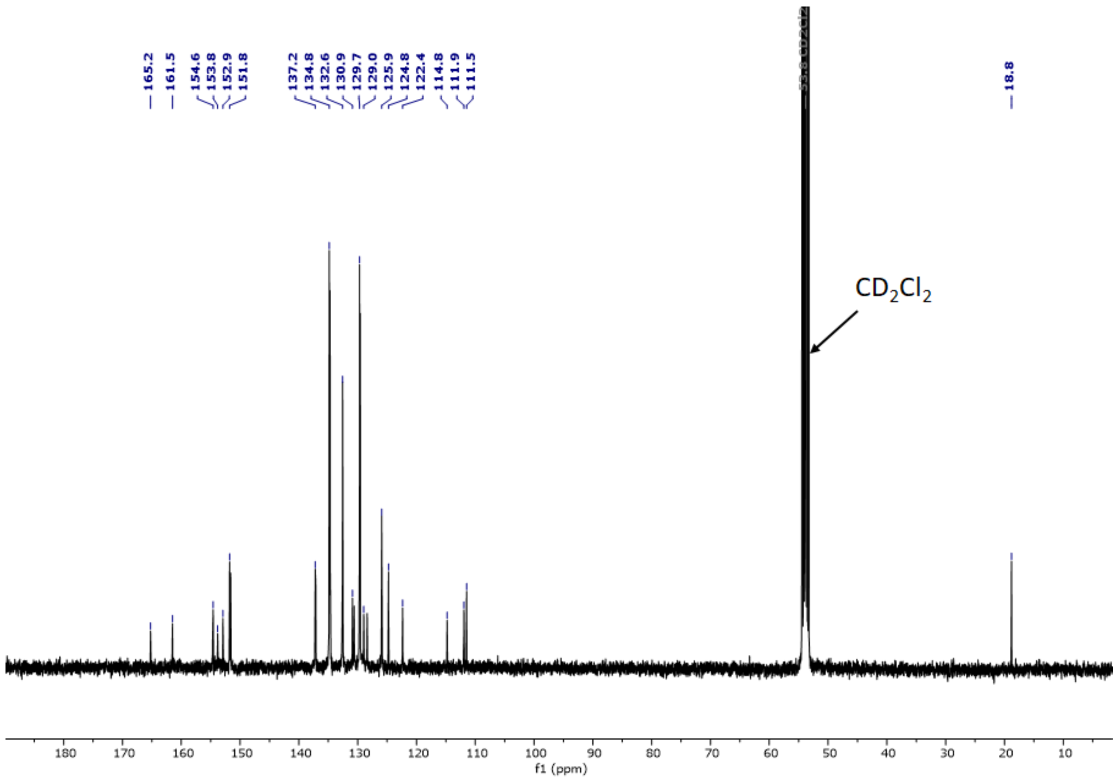

Figure S17. $^{13}C\{^1H\}$ NMR spectrum of **5** in CD_2Cl_2 at room temperature.

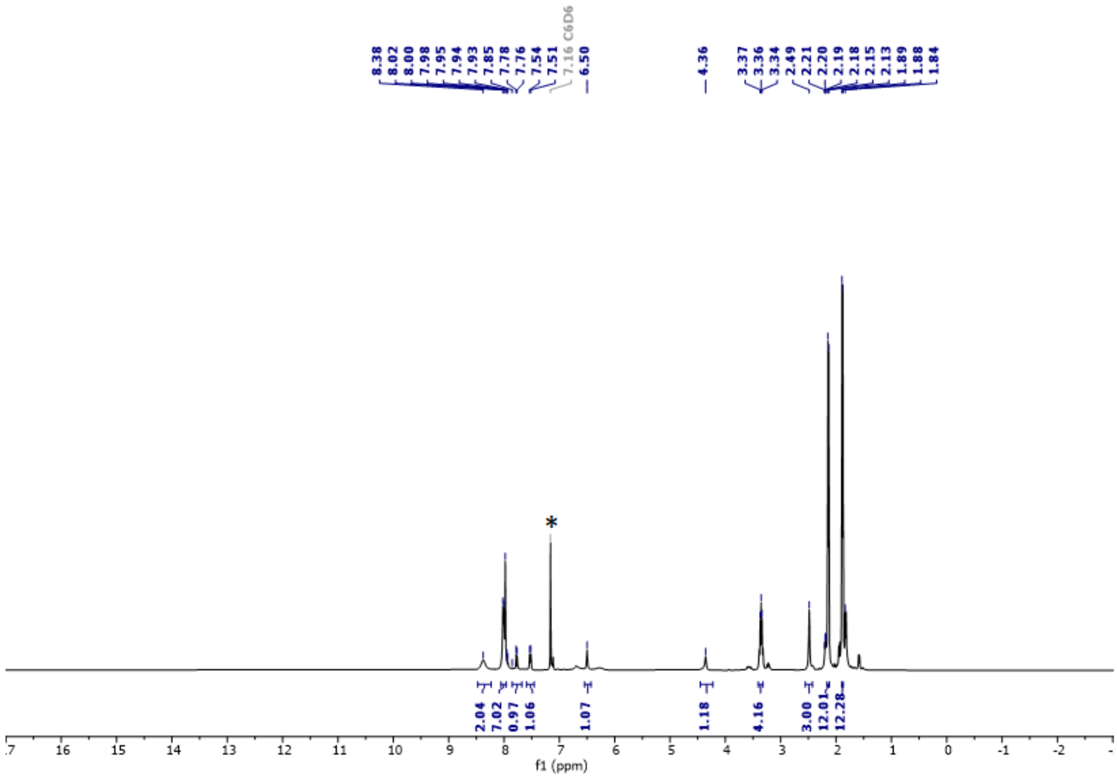

Figure S18. ^1H NMR spectrum of **6** in C_6D_6 at room temperature. *, residual protio solvent signal.

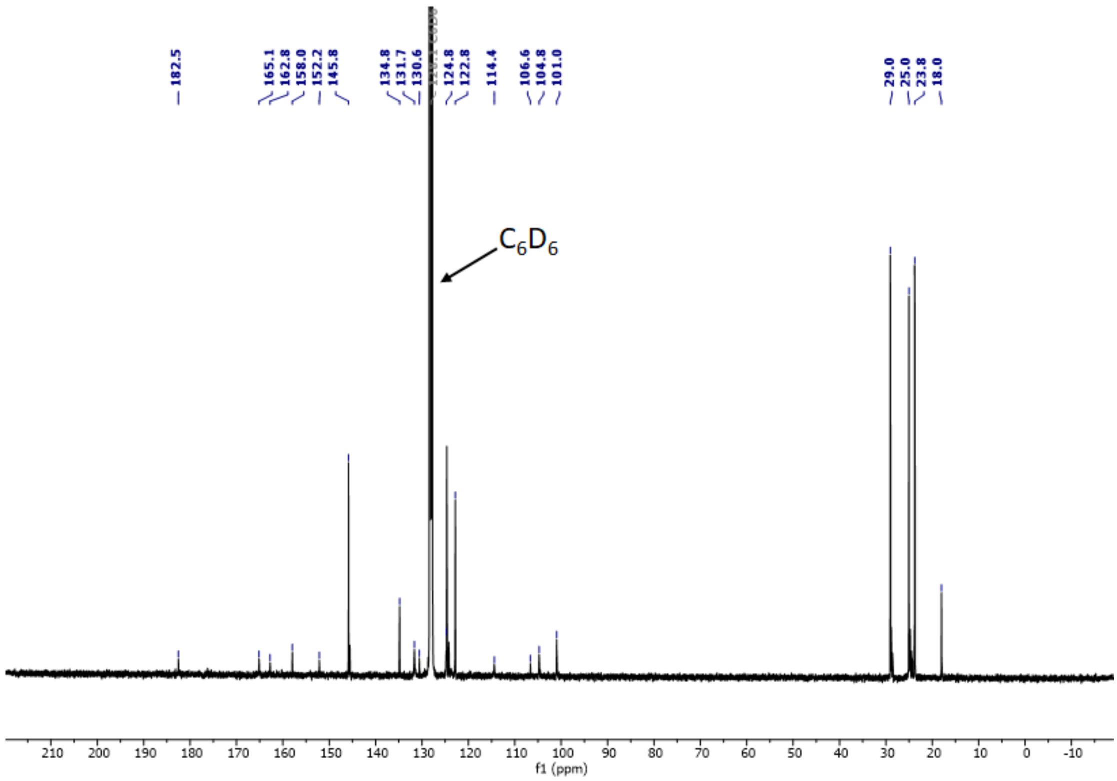

Figure S19. $^{13}C\{^1H\}$ NMR spectrum of **6** in C_6D_6 at room temperature.

III. IR spectra

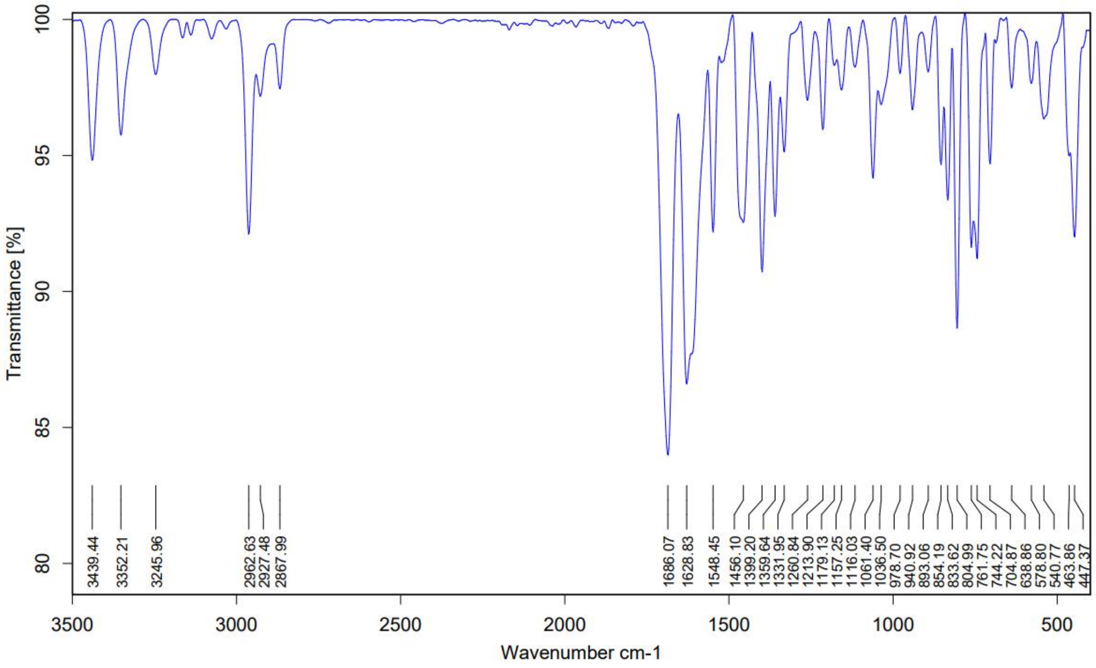

Figure S20. IR spectrum of complex **1**.

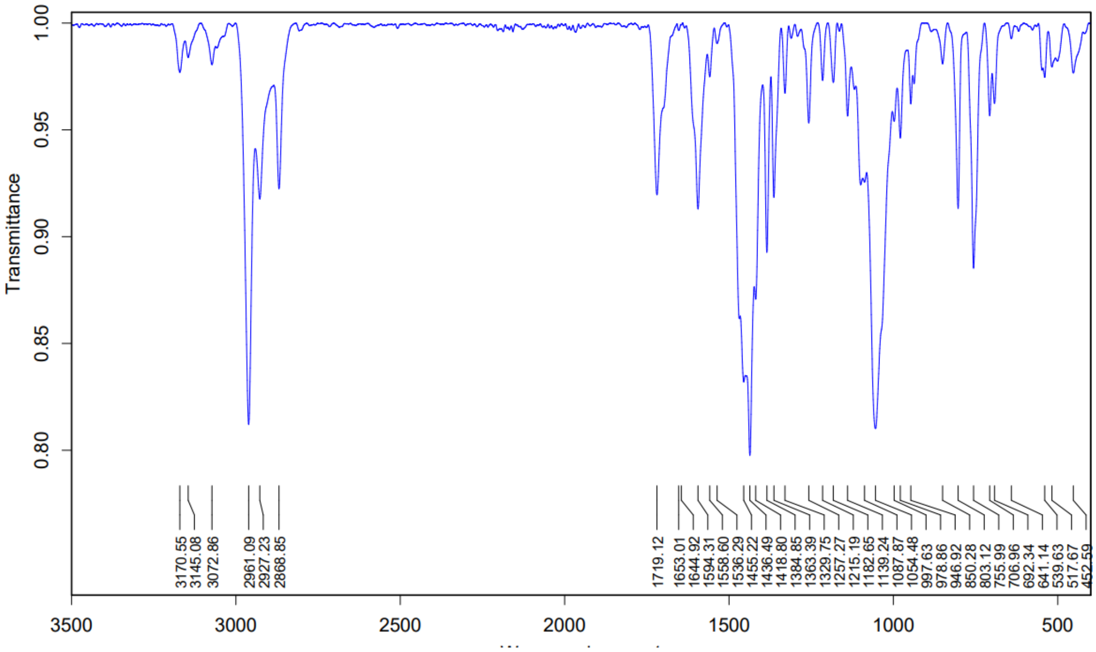

Figure S21. IR spectrum of complex **2**.

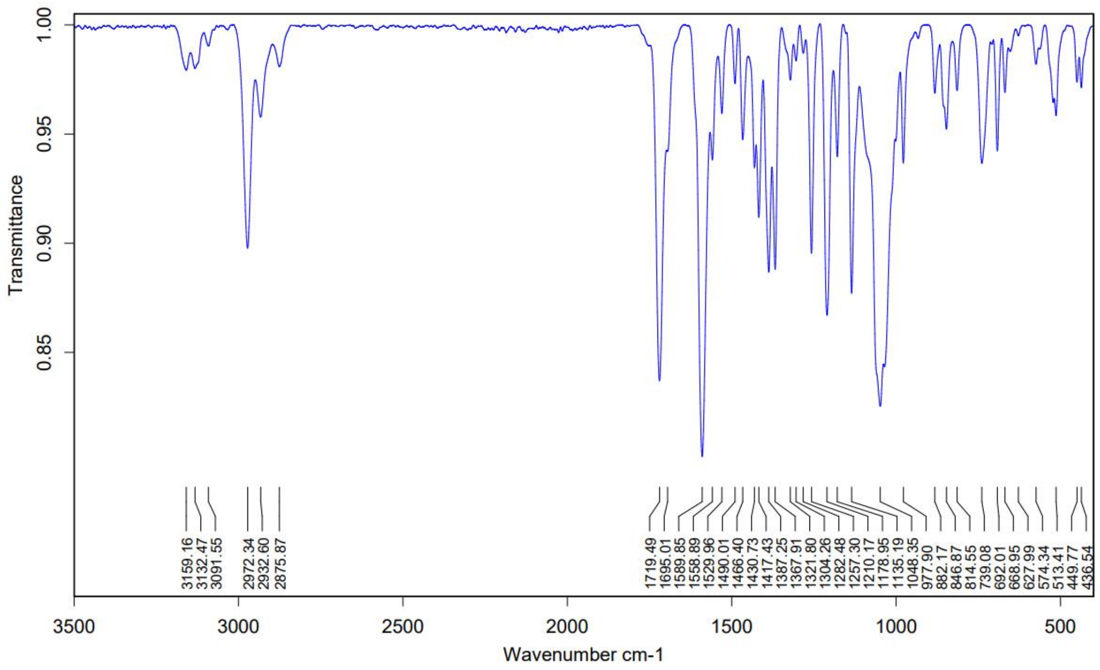

Figure S22. IR spectrum of complex **3**

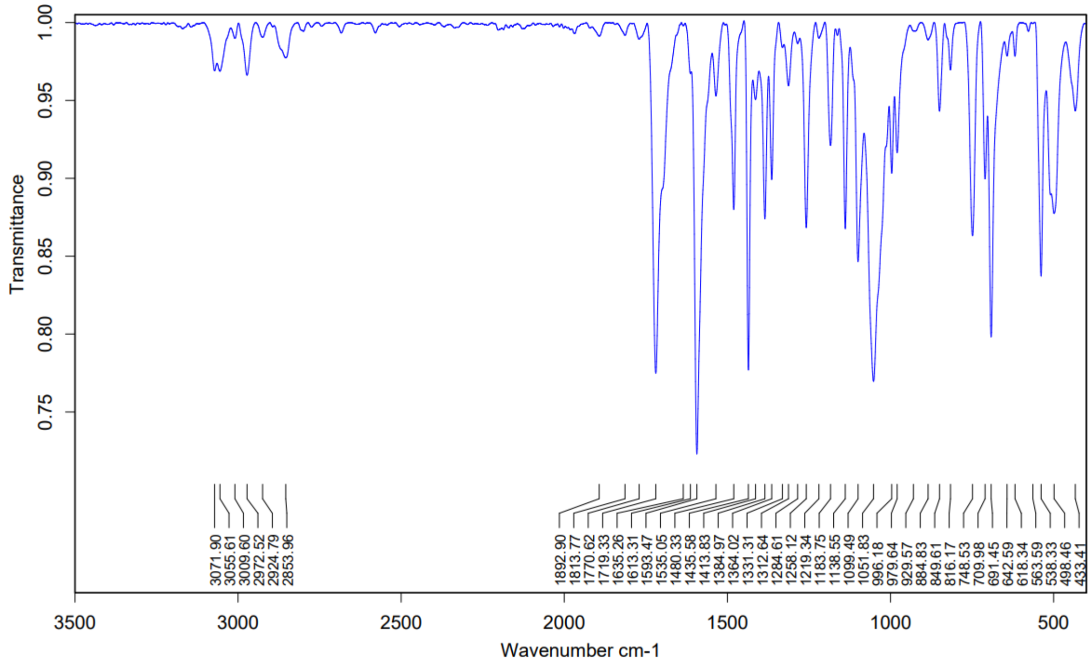

Figure S23. IR spectrum of complex **4**.

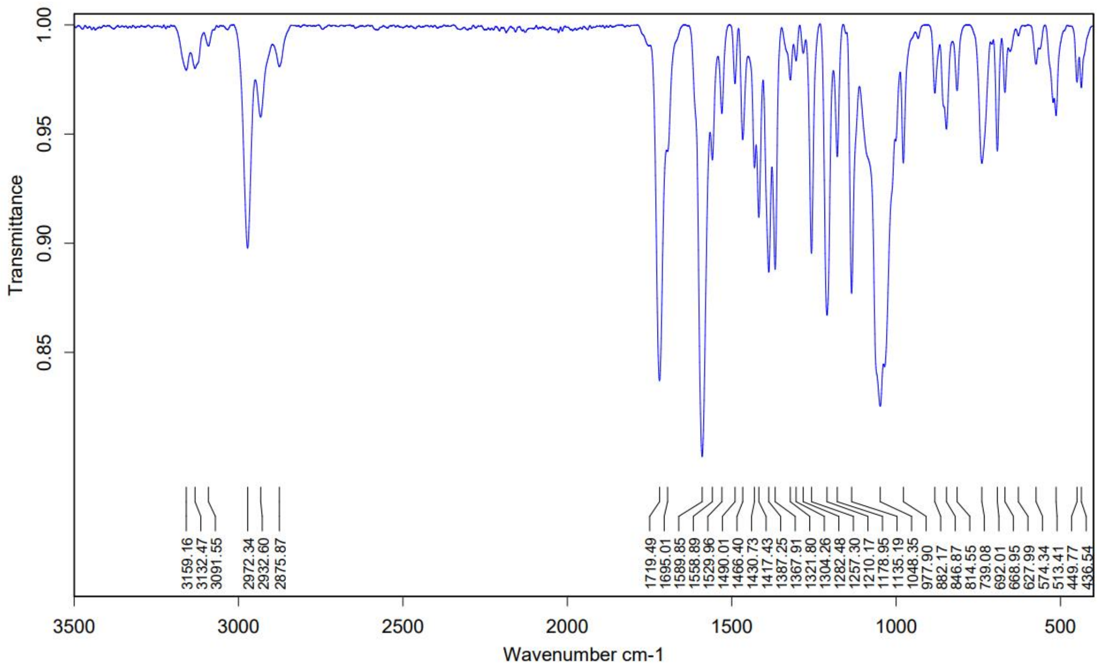

Figure S24. IR spectrum of complex **5**.

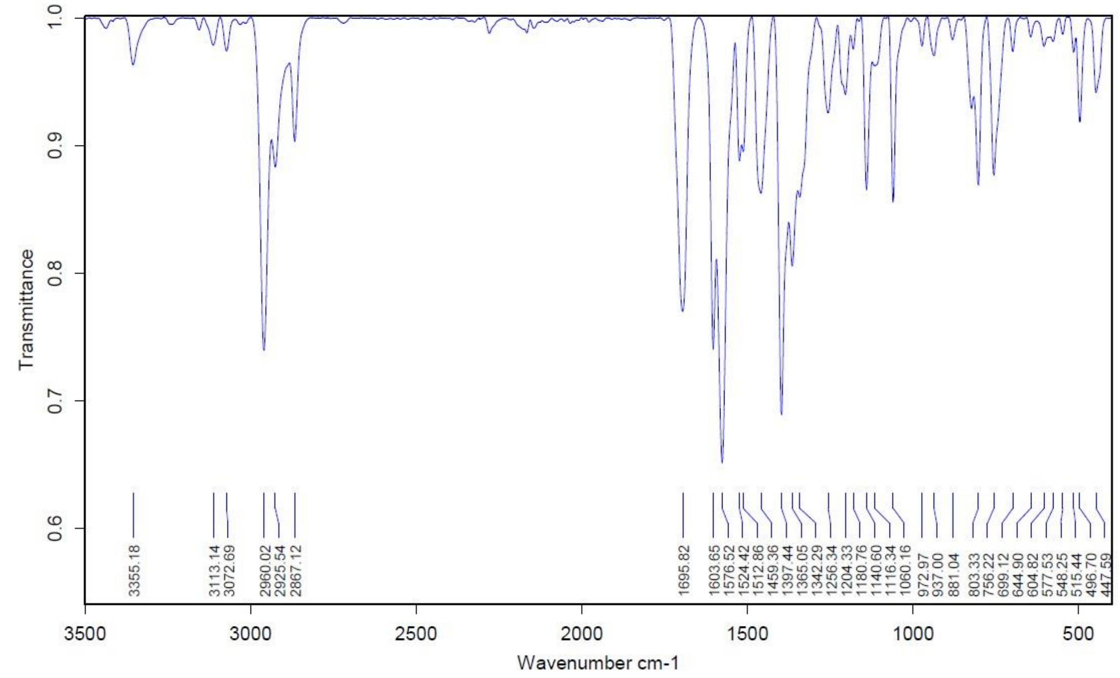

Figure S25. IR spectrum of complex **6**.

IV. Mass Spectra

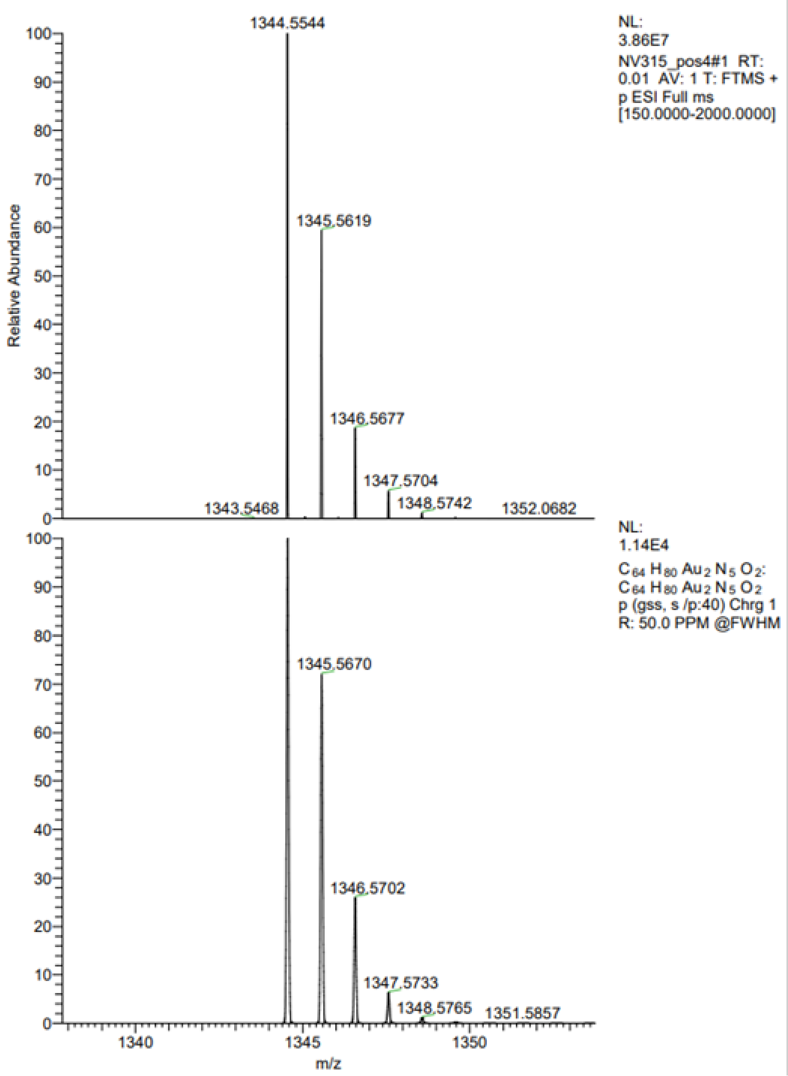

Figure S26. HRMS (ESI) spectra of complex **2**. Shown is the molecular ion peak $[M\text{-}BF_4]^+$ Top: experimental spectrum, Bottom: simulated signals.

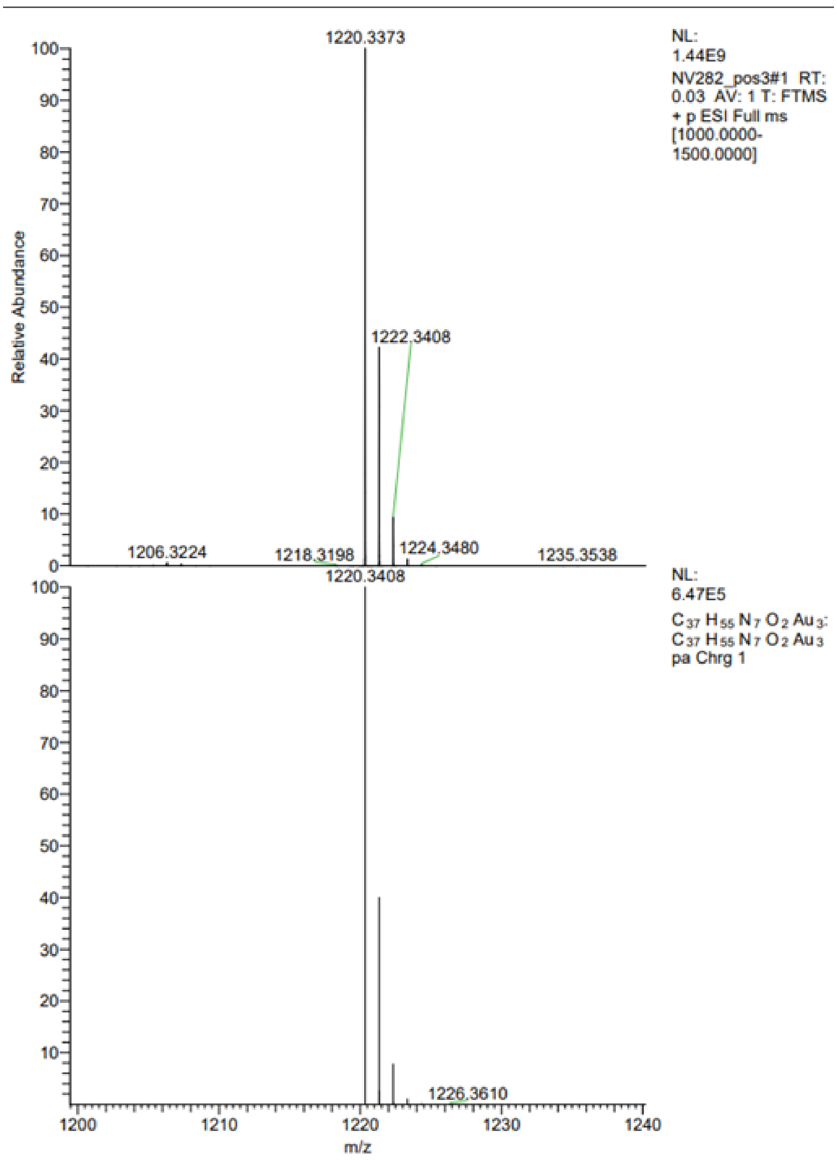

Figure S27. HRMS (ESI) spectra of complex **3**. Shown is the molecular ion peak $[M\text{-}BF_4]^+$ Top: experimental spectrum, Bottom: simulated signals.

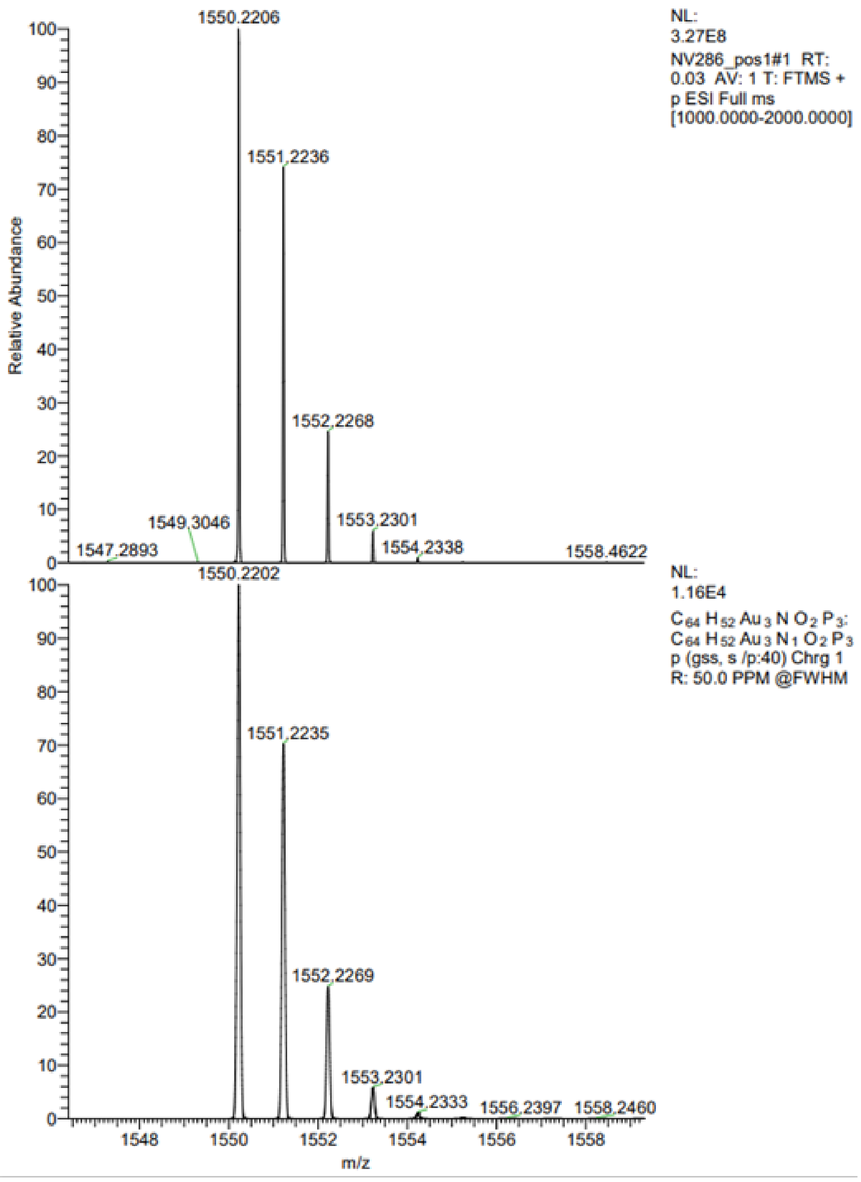

Figure S28. HRMS (ESI) spectra of complex **4**. Shown is the molecular ion peak $[M\text{-}BF_4]^+$ Top: experimental spectrum, Bottom: simulated signals.

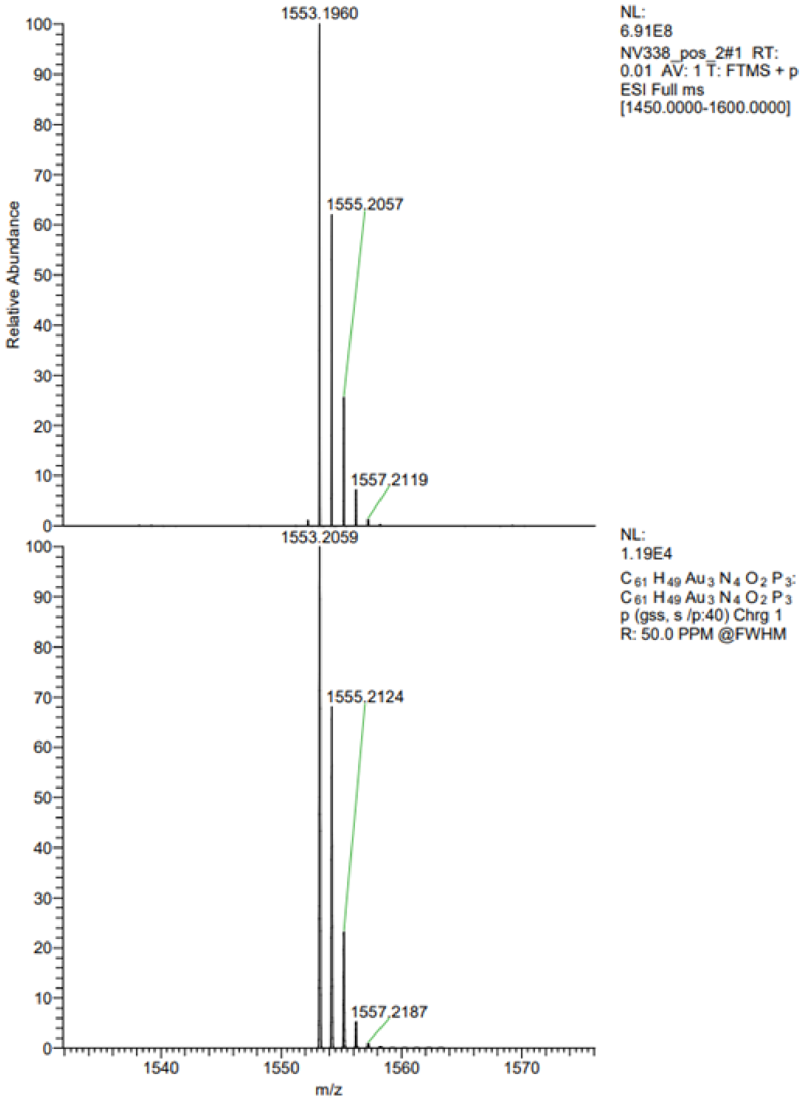

Figure S29. HRMS (ESI) spectra of complex **5**. Shown is the molecular ion peak $[M-BF_4]^+$ Top: experimental spectrum, Bottom: simulated signals.

V. X-ray crystallography

V.1 General methods

Suitable crystals for the X-ray analysis of all compounds were obtained as described above. A suitable crystal was covered in mineral oil (Aldrich) and mounted on a glass fibre. The crystal was transferred directly to the cold stream of a STOE StadiVari (100 K or 150 K) diffractometer. All structures were solved by using the program SHELXS/T[7,8] and Olex2.[9] The remaining non-hydrogen atoms were located from successive difference Fourier map calculations. The refinements were carried out by using full-matrix least-squares techniques on F^2 by using the program SHELXL.[8,9] The H-atoms were introduced into the geometrically calculated positions (SHELXL procedures) unless otherwise stated and refined riding on the corresponding parent atoms. In each case, the locations of the largest peaks in the final difference Fourier map calculations, as well as the magnitude of the residual electron densities, were of no chemical significance. Summary of the crystal data, data collection and refinement for all compounds are given in Table S1.

Crystallographic data for the structures reported in this paper have been deposited with the Cambridge Crystallographic Data Centre as a supplementary publication no. 2341059-2341062 (**1-5**) and 2374861 (**6**). Copies of the data can be obtained free of charge on application to CCDC, 12 Union Road, Cambridge CB21EZ, UK (fax: (+(44)1223-336-033; email: deposit@ccdc.cam.ac.uk).

V.2. Table S1: Summary of crystal data

Compound	**1**	**3**	**4**	**5**
Chemical formula	$C_{40}H_{47}AuCl_9N_3O_2$	$C_{38}H_{57}Au_3BCl_2F_4N_7O_2$	$C_{70}H_{65}Au_3BF_4N_4O_{3.5}P_3$	$C_{65}H_{59}Au_3BF_4N_4O_3P_3$
Formula weight	1117.82	1392.51	1746.85	1714.78
Temperature/K	100	100	100	100
Crystal system	orthorhombic	monoclinic	monoclinic	triclinic
Space group	$P2_12_12_1$	$P2_1/c$	$P2_1/c$	$P\bar{1}$
a/Å	13.9636(4)	11.2062(2)	16.5661(11)	13.6908(17)
b/Å	17.9666(6)	30.8018(6)	20.5903(10)	14.3575(18)
c/Å	18.5704(6)	13.2126(2)	17.9789(11)	18.3540(15)
α/°				78.990(8)
β/°		96.4440(10)	90.137(5)	67.808(8)
γ/°				65.154(8)
Volume/Å^3	4658.9(3)	4531.79(14)	6132.6(6)	3029.1(6)
Z	4	4	4	2
ρ_{calc}g/cm^3	1.594	2.041	1.892	1.880
μ/mm^{-1}	3.710	9.863	7.300	7.389
F(000)	2224.0	2648.0	3372.0	1648.0
Colour/shape	Orange/fragment	Yellow/plate	Colourless/needle	Yellow/prism
Crystal size/mm^3	0.146 × 0.113 × 0.073	0.415 × 0.191 × 0.038	0.322 × 0.1 × 0.064	0.078 × 0.051 × 0.026
Radiation	Mo Kα (λ = 0.71073)	Mo Kα (λ = 0.71073)	Mo Kα (λ = 0.71073)	Mo Kα (λ = 0.71073)
2Θ range for data collection/°	3.154 to 63.102	5.036 to 58.342	3.882 to 63.578	3.626 to 60.366
Index ranges	-19 ≤ h ≤ 19, -21 ≤ k ≤ 25, -22 ≤ l ≤ 26	-15 ≤ h ≤ 15, -41 ≤ k ≤ 41, -17 ≤ l ≤ 17	-24 ≤ h ≤ 23, -29 ≤ k ≤ 28, -25 ≤ l ≤ 24	-18 ≤ h ≤ 19, -19 ≤ k ≤ 18, -25 ≤ l ≤ 22
Reflections collected	31501	64031	70196	41832
Independent reflections	12476 [R_{int} = 0.0355, R_{sigma} = 0.0626]	11098 [R_{int} = 0.0598, R_{sigma} = 0.0346]	17777 [R_{int} = 0.0423, R_{sigma} = 0.0504]	15004 [Rint = 0.0355, Rsigma = 0.0577]
Data/restraints/parameters	12476/0/485	11098/310/566	17777/322/833	15004/2087/995
Goodness-of-fit on F^2	1.020	1.022	1.053	1.024
Final R indexes [I>=2σ (I)]	R_1 = 0.0452, wR_2 = 0.0884	R_1 = 0.0551, wR_2 = 0.1380	R_1 = 0.0404, wR_2 = 0.0845	R1 = 0.0409, wR2 = 0.0885
Final R indexes [all data]	R_1 = 0.0681, wR_2 = 0.0972	R_1 = 0.0793, wR_2 = 0.1621	R_1 = 0.0797, wR_2 = 0.1162	R1 = 0.0773, wR2 = 0.1021
Largest diff. peak/hole / e Å^{-3}	1.03/-0.79	3.19/-2.56	2.60/-2.08	1.59/-1.75

Compound	**6**
Chemical formula	$C_{141}H_{192}Cu_3N_9O_{13.5}$
Formula weight	2419.64
Temperature/K	120
Crystal system	orthorhombic
Space group	$P2_12_12_1$
a/Å	13.9189(2)
b/Å	18.6249(3)
c/Å	53.6763(13)
α/°	
β/°	
γ/°	
Volume/Å^3	13914.9(5)
Z	4
ρ_{calc}g/cm^3	1.155
μ/mm^{-1}	0.514
F(000)	5184.0
Colour/shape	Yellow/block
Crystal size/mm^3	0.262 × 0.107 × 0.072
Radiation	Mo Kα (λ = 0.71073)
2Θ range for data collection/°	3.156 to 50.5
Index ranges	-16 ≤ h ≤ 16, -22 ≤ k ≤ 22, -64 ≤ l ≤ 57
Reflections collected	65636
Independent reflections	24882 [R_{int} = 0.0314, R_{sigma} = 0.0509]
Data/restraints/parameters	24882/2335/1591
Goodness-of-fit on F^2	1.022
Final R indexes [I>=2σ (I)]	R_1 = 0.0616, wR_2 = 0.1580
Final R indexes [all data]	R_1 = 0.0975, wR_2 = 0.1833
Largest diff. peak/hole / e Å^{-3}	1.16/-0.41
Flack parameter	0.52(2)

V.3 Crystal structures

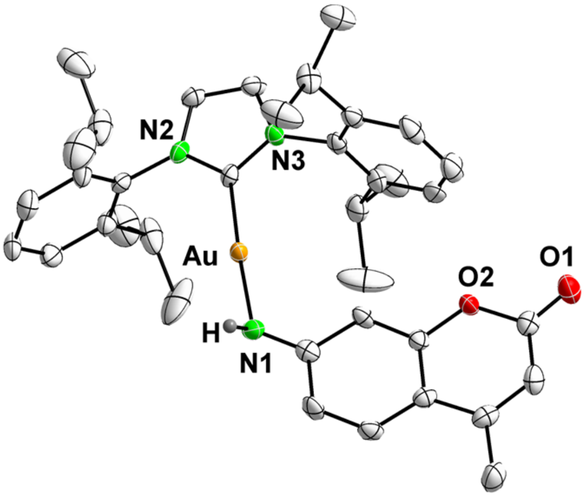

Figure S30. Molecular structure of IPrNHC coordinated gold imido complex **1** in the solid state. Hydrogen atoms (except N-H proton) and non-coordinating solvents are removed for clarity. Selected bond distances (Å) and angles (°): Au–C11 1.981(6), Au–N1 1.997(6); C11–Au–N1 176.8(3). Thermal ellipsoids are drawn at 50 % probability.

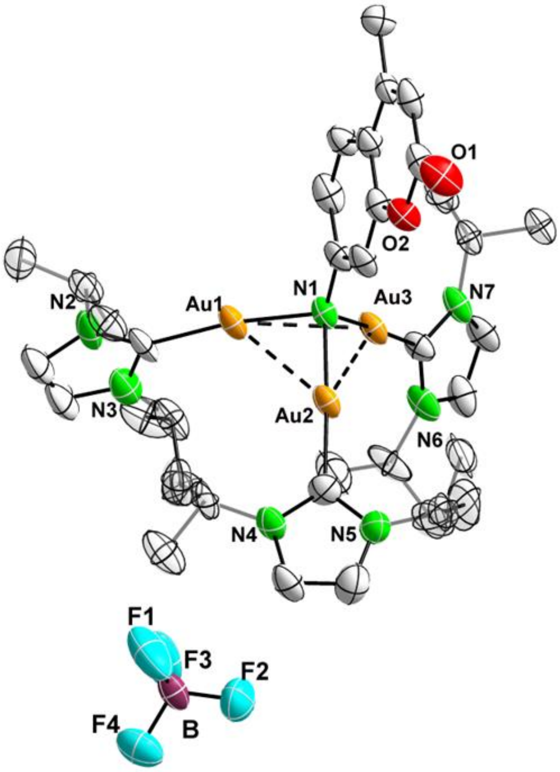

Figure S31: Molecular structure of *i*-PrNHC coordinated gold imido cluster **3** in the solid state. Hydrogen atoms and non-coordinating solvents are removed for clarity. Selected bond distances (Å) and angles (°): Au1–Au2 3.0042(6), Au2–Au3 3.1721(5), Au3–Au1 3.0956(6), Au1–N1 2.055(8), Au2–N1 2.053(9), Au3–N1 2.067(8), Au1–C11 2.002(10), Au2–C20 1.991(10), Au3–C29 2.004(10); Au1–N1–Au2 94.0(3), Au2–N1–Au3 100.7(4), Au3–N1–Au1 97.4(3), N1–Au1–C11 171.1(4), N1–Au2–C20 177.5(4), N1–Au3–C29 178.7(4). Thermal ellipsoids are drawn at 50 % probability.

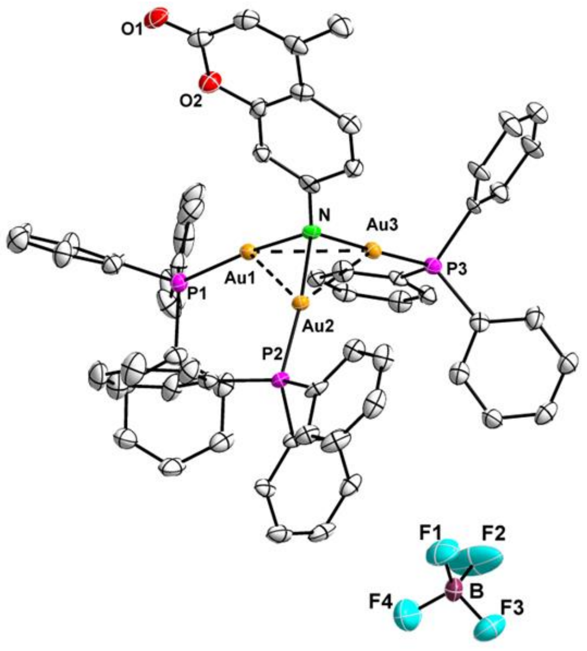

Figure S32. Molecular structure of PPh_3 coordinated gold imido cluster **4** in the solid state. Hydrogen atoms and non-coordinating solvent molecules are removed for clarity. Selected bond distances (Å) and angles (°): Au1–Au2 3.0551(4), Au2–Au3 3.2081(4), Au3–Au1 2.9339(4), Au1–N 2.056(6), Au2–N 2.034(6), Au3–N 2.057(6), Au1–P1 2.222(2), Au2–P2 2.236(2), Au3–P3 2.226(2); Au1–N–Au2 96.6(2), Au2–N–Au3 103.3(3), Au3–N–Au1 91.0(2), N–Au1–P1 173.0(2), N–Au2–P2 172.1(2), N–Au3–P3 174.2(2). Thermal ellipsoids are drawn at 50 % probability.

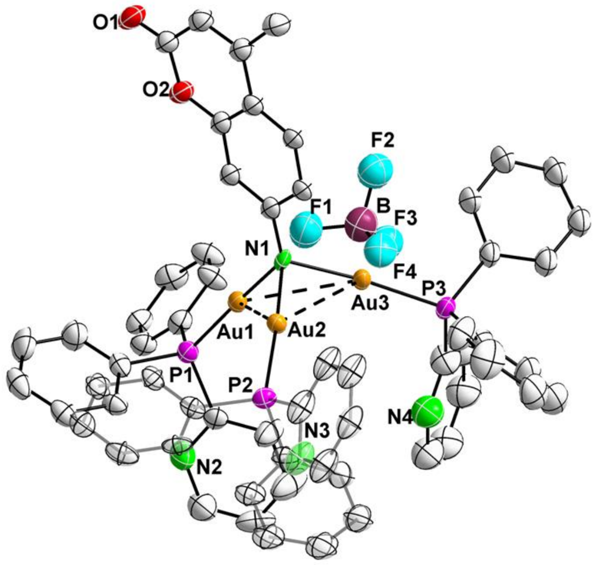

Figure S33: Molecular structure of $PyPPh_2$ coordinated gold imido cluster **5** in the solid state. Hydrogen atoms and non-coordinating solvents are removed for clarity. Selected bond distances (Å) and angles (°): Au1–Au2 2.9752(4), Au2–Au3 3.0046(4), Au3–Au1 3.1998(5), Au1–N1 2.061(5), Au2–N1 2.068(5), Au3–N1 2.059(5), Au1–P1 2.240(2), Au2–P2 2.229(2), Au3–P3 2.239(2); Au1–N1–Au2 92.2(2), Au2–N1–Au3 93.5(2), Au1–N1–Au3 101.9(2), N1–Au1–P1 175.99(14), N1–Au2–P2 172.28(15), N1–Au3–P3 175.40(15). Thermal ellipsoids are drawn at 50 % probability.

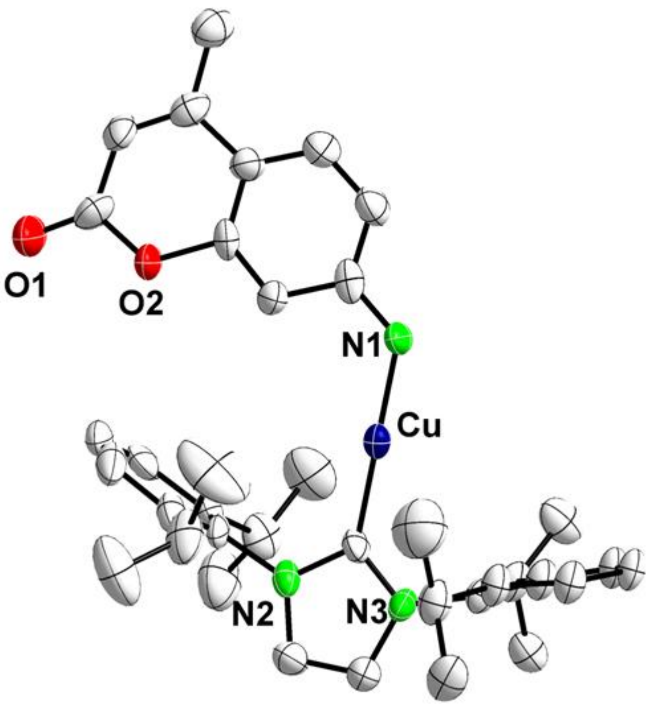

Figure S34. Molecular structure of IPrNHC coordinated copper(I) imido complex **6** in the solid state. Hydrogen atoms and non-coordinating solvents are removed for clarity. Selected bond distances (Å) and angles (°): Cu–C11 1.874(8), Cu–N1 1.843(6); C11–Cu–N1 176.5(3). Thermal ellipsoids are drawn at 50 % probability.

VI. Photoluminescence data

PL measurements were carried out on a PTI QuantaMasterTM 8075-22 fluorometer with double excitation and emission monochromators (HORIBA Jobin Yvon GmbH). The samples (polycrystalline solids) were each sealed under inert atmosphere in NMR tubes with a J. Young valve (material Suprasil® quartz glass). The tube was placed in a glass dewar vessel (equipped with a suprasil finger on the bottom where spectroscopy takes place) which was filled with liquid nitrogen for measurements at 77 K. The temperature-dependent emission spectra (Figure 6a) were measured with a closed-cycle helium cryostat. For emission detection, a R928 photomultiplier (250–800 nm) (HORIBA Jobin Yvon GmbH) was used. All spectra were corrected for the wavelength dependent response of the detector (in relative photon flux units) and the spectrometer. For detection of the emission decay traces, the sample was excited with either a Delta DiodeTM (HORIBA Jobin Yvon GmbH, Model DD-370, λ_{exc} = 371 nm, pulse <2 ns, 2 µW) for fluorescence lifetimes or a PTI XenonFlash^{TM} (set before the emission monochromators, frequency max. 300 Hz) for phosphorescence decay times. In case of using the Delta Diode, the signal was recorded until a satisfying signal-to-noise ratio was obtained. When using the Xenon Flash lamp, 10000 traces were recorded.

Quantum yields of compounds in solution were determined by following the procedure reported by Friend *et al.* wherein the diffuse reflection and emission of both the sample and a blank were recorded at the same excitation wavelength.[22] The reflected and emitted photons were integrated by using an Ulbricht sphere which resulted in the absolute quantum yield. Corrections were made for the detector sensitivity, spectral power of the excitation source and reflection behavior of the Ulbricht sphere.

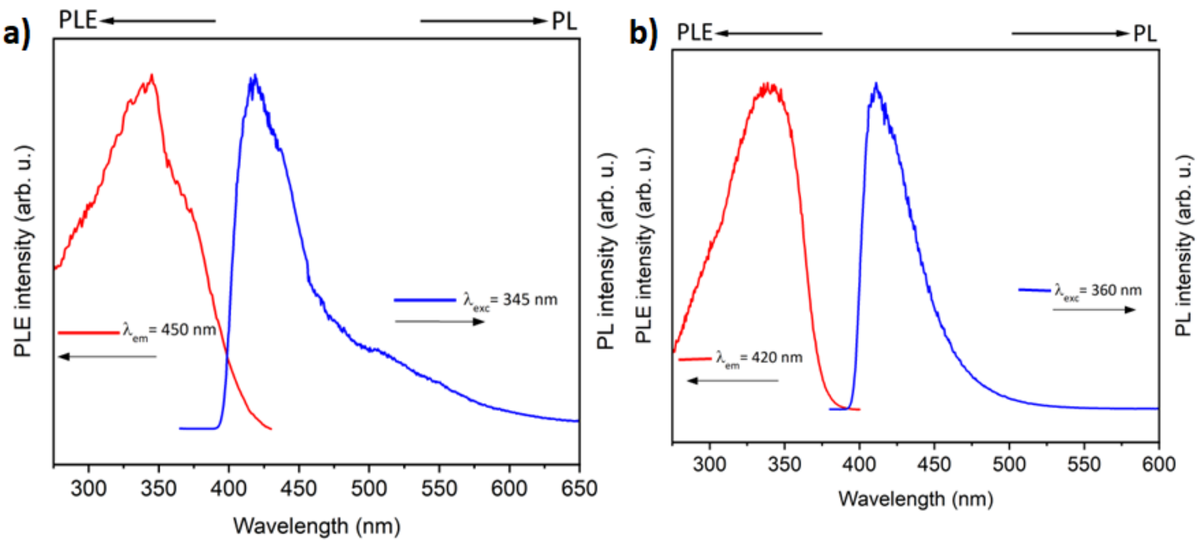

Figure S35. Normalized photoluminescence excitation (PLE) and emission (PL) spectra of DCM solution of the copper(I) complex **6** a) at 77 K; b) at room temperature.

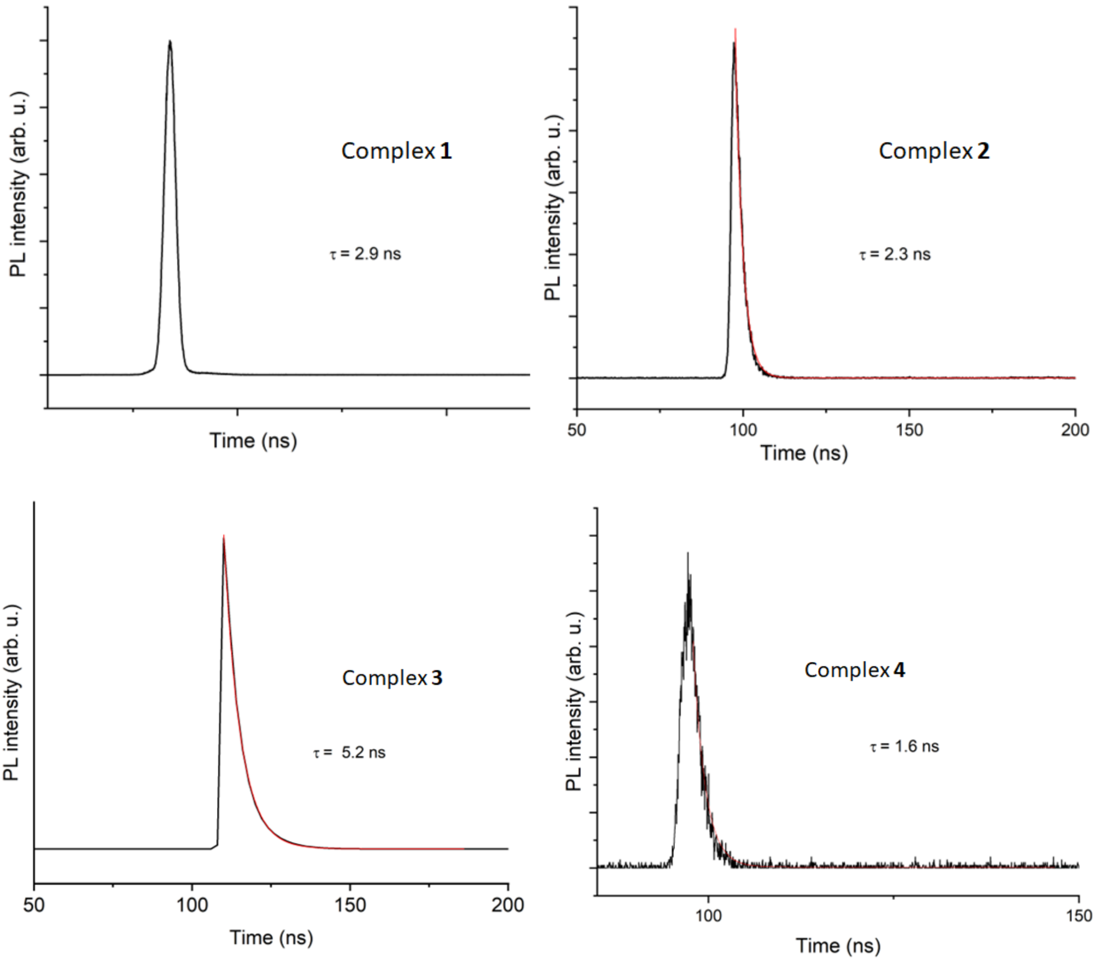

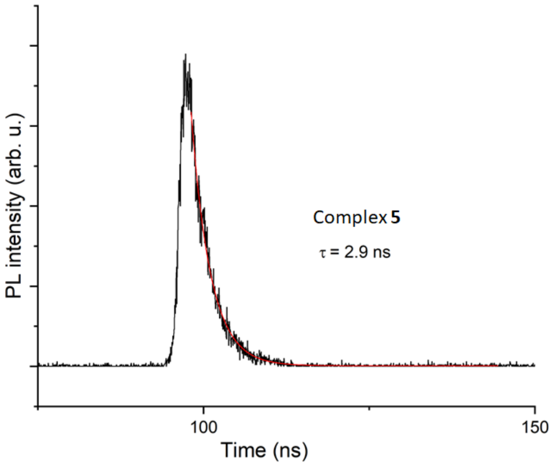

Figure S36. Emission decay traces of DCM solutions of complexes **1**-**5** in DCM solutions at room temperature.

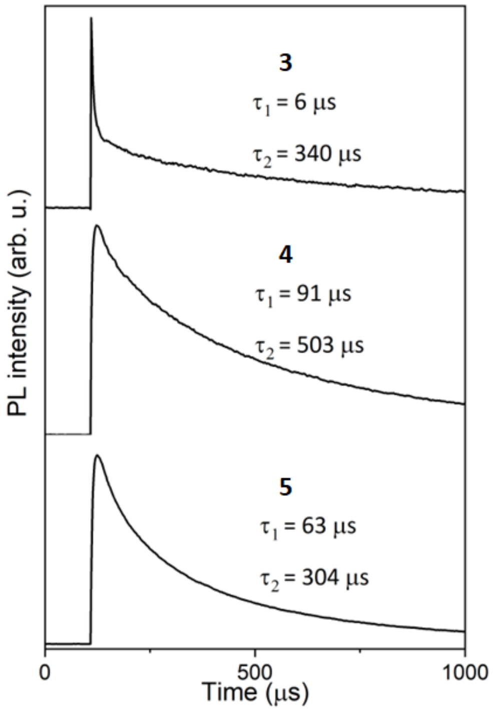

Figure S37. Emission decay traces of DCM solutions of trinuclear complexes **3**-**5** at 77 K.

VII. Quantum Chemical Calculations

Quantum chemical calculations at the density functional theory (DFT) level were performed with TURBOMOLE.[10,11] Initial structures were taken from XRD data, except for compound **2**, for which no crystal structure is available, and optimized. Starting from the optimized structure of **1**, the structure of **1** with perpendicular coumarin and NHC ligand planes was generated by rotating the coumarin moiety by 90° around the Au-N bond and reoptimizing the structure under the constraint of a fixed dihedral angle N24-C25-Au22-C4 (numbers

refer to atom numbers in optimized-structures.txt). The PBE0 hybrid functional[13,14] and def2-TZVP basis sets[15] together with the effective core potential (ECP) for Au (ECP-60) were used.[16] Optimized structures of all compounds considered in the calculations are given in the separate ASCII file optimized-structures.txt.

The resolution of the identity approximation for the Coulomb part (RI-*J*)[17] in combination with the corresponding auxiliary basis sets as well as the multipole-accelerated RI-*J* approximation (MARI-*J*)[18] were employed. The D4 dispersion correction was used in all cases.[19]

Self-consistent field (SCF) thresholds were set to 10^{-8} E_h, and large grids (gridsize 5) were used for the numerical integration of the exchange-correlation terms.[20] The convergence criterion for the root mean square of the density matrix was set to 10^{-7} E_h. Difference densities were analyzed using the Peak Analyzing Machine described in Ref. [21].[21]

Counter ions were not considered in the calculations.

Table S2: Selected experimental (Exp.) and calculated (Calc., PBE0/def2-TZVP) bond distances (Å) and angles (°) of compounds **1** and **3**-**5**. Note that no experimental data is available for compound **2**. Atom labels refer to Figures S30-S33. Calculated values are given in ascending order.

	**1**		**3**		**4**		**5**	
	Exp.	Calc.	Exp.	Calc.	Exp.	Calc.	Exp.	Calc.
**Au-Coumarin**	**Au-N11** 1.997(6)	2.003	**Au1-N1** 2.055(8)<br>**Au2-N1** 2.053(9)<br>**Au3-N1** 2.067(8)	2.051, 2.052, 2.054	**Au1–N** 2.056(6)<br>**Au2–N** 2.034(6)<br>**Au3–N** 2.057(6)	2.057, 2.068, 2.082	**Au1–N1** 2.061(5)<br>**Au2–N1** 2.068(5)<br>**Au3–N1** 2.059(5),	2.065, 2.070, 2.075
**Au-Ligand**	**Au-C11** 1.981(6)	1.979	**Au1–C11** 2.002(10)<br>**Au2–C20** 1.991(10)<br>**Au3–C29** 2.004(10)	1.993, 1.993, 1.999	**Au1–P1** 2.222(2)<br>**Au2–P2** 2.236(2)<br>**Au3–P3** 2.226(2)	2.249, 2.250, 2.251	**Au1–P1** 2.240(2)<br>**Au2–P2** 2.229(2)<br>**Au3–P3** 2.239(2)	2.246, 2.251, 2.252
**Au-Au**	-	-	**Au1-Au2** 3.0042(6)<br>**Au2-Au3** 3.1721(5)<br>**Au3-Au1** 3.0956(6)	3.128, 3.128, 3.159	**Au1–Au2** 3.0551(4)<br>**Au2–Au3** 3.2081(4)<br>**Au3–Au1** 2.9339(4)	2.974, 3.056, 3.060	**Au1–Au2** 2.9752(4)<br>**Au2–Au3** 3.0046(4)<br>**Au3–Au1** 3.1998(5)	2.952, 3.010, 3.087
**Au-Coumarin-Au**	**-**	-	**Au1–N1–Au2** 94.0(3)<br>**Au2–N1–Au3** 100.7(4)<br>**Au3–N1–Au1** 97.4(3)	99.3, 99.3, 100.6	**Au1–N–Au2** 96.6(2)<br>**Au2–N–Au3** 103.3(3)<br>**Au3–N–Au1** 91.0(2)	91.5, 95.4, 95.6	**Au1–N1–Au2** 92.2(2)<br>**Au2–N1–Au3** 93.5(2)<br>**Au1–N1–Au3** 101.9(2)	90.8, 93.4, 96.4

Table S2 continued

**Coumarin-Au-Ligand**	**C11-Au-N1** 176.8(3)	175.6	**N1–Au1–C11** 171.1(4)	177.4, 178.3, 178.9	**N–Au1–P1** 173.0(2)	169.7, 170.0, 175.0	**N1–Au1–P1** 175.99(14)	167.4, 172.0, 174.5
			**N1–Au2–C20** 177.5(4)		**N–Au2–P2** 172.1(2)		**N1–Au2–P2** 172.28(15)	
			**N1–Au3–C29** 178.7(4)		**N–Au3–P3** 174.2(2)		**N1–Au3–P3** 175.40(15)	

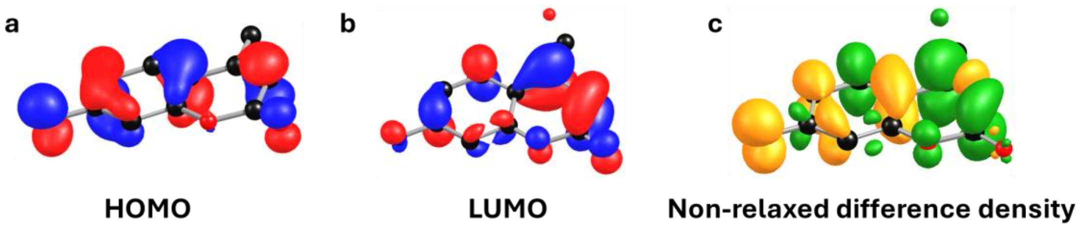

Figure S38: Highest occupied molecular orbital (a, HOMO), lowest unoccupied molecular orbital (b, LUMO) (contours at 0.05 a.u.) and non-relaxed difference density (c, contours at 0.002 a.u.) between the lowest energy excited singlet and the ground state of 7-amino-4-methylcoumarin (PBE0/def2-TZVP). For the latter, orange color indicates a surplus of electron density for the ground state, green for the excited state. Hydrogen atoms are omitted for clarity. Carbon black, nitrogen blue, oxygen red.

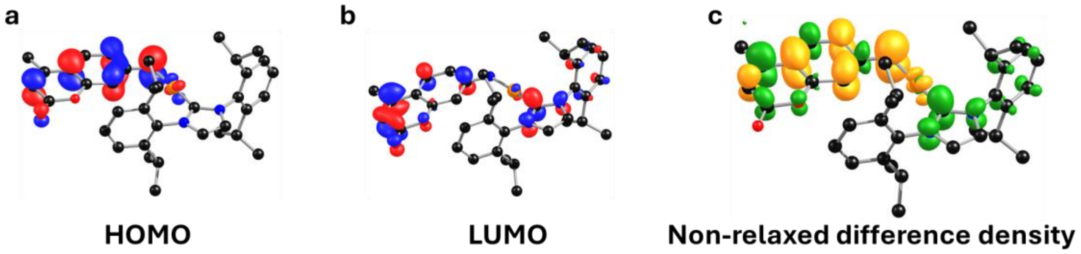

Figure S39: Highest occupied molecular orbital (a, HOMO), lowest unoccupied molecular orbital (b, LUMO) (contours at 0.05 a.u.) and non-relaxed difference density (c, contours at 0.002 a.u.) between the lowest energy excited singlet and the ground state of compound **1** (PBE0/def2-TZVP). For the latter, orange color indicates a surplus of electron density for the ground state, green for the excited state. Hydrogen atoms are omitted for clarity. Carbon black, nitrogen blue, oxygen red, gold orange.

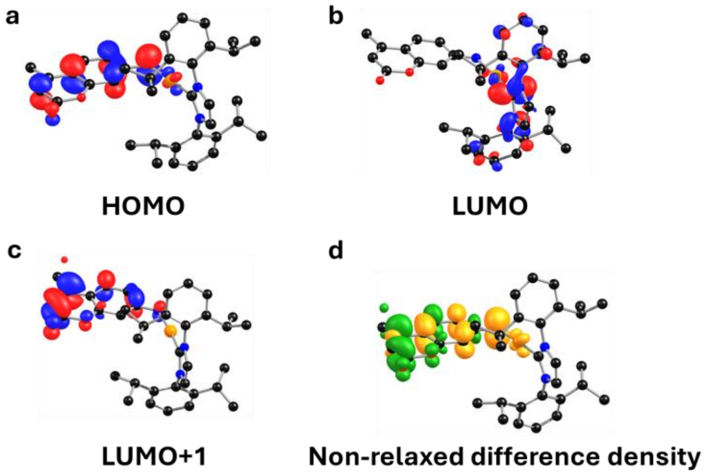

Figure S40: Highest occupied molecular orbital (a, HOMO), lowest unoccupied molecular orbital (b, LUMO), LUMO+1 (c) (contours at 0.05 a.u.) and non-relaxed difference density (d, contours at 0.002 a.u.) between the two lowest energy singlet vertical excitations (weighted by their oscillator strength) and the ground state of compound **1** with perpendicularly oriented coumarin and NHC ligand planes (PBE0/def2-TZVP). For the latter, orange color

indicates a surplus of electron density for the ground state, green for the excited state. Hydrogen atoms are omitted for clarity. Carbon black, nitrogen blue, oxygen red, gold orange.

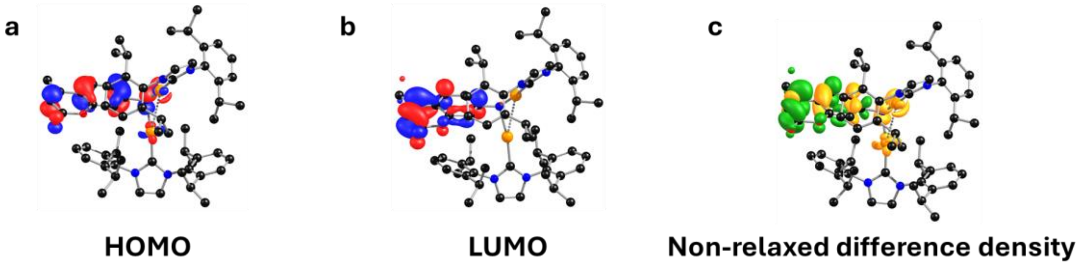

Figure S41: Highest occupied molecular orbital (a, HOMO), lowest unoccupied molecular orbital (b, LUMO) (contours at 0.05 a.u.) and non-relaxed difference density (c, contours at 0.002 a.u.) between the lowest energy excited singlet and the ground state of compound **2** (PBE0/def2-TZVP). For the latter, orange color indicates a surplus of electron density for the ground state, green for the excited state. Hydrogen atoms are omitted for clarity. Carbon black, nitrogen blue, oxygen red, gold orange.

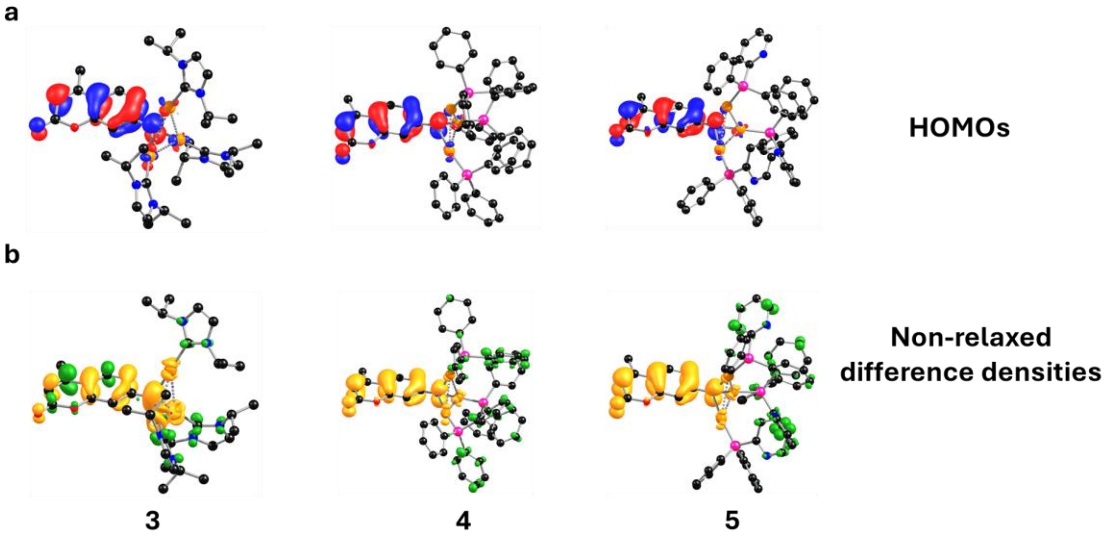

Figure S42: Highest occupied molecular orbitals (a, HOMOs, contours at 0.05 a.u.) and non-relaxed difference densities (c, contours at 0.002 a.u.) between the ten lowest energy excited singlet vertical excitations (weighted by their oscillator strength) and the ground state of compounds **3-5** (PBE0/def2-TZVP). For the latter, orange color indicates a surplus of electron density for the ground state, green for the excited state. Hydrogen atoms are omitted for clarity. Carbon black, nitrogen blue, oxygen red, phosphorous magenta, gold orange.

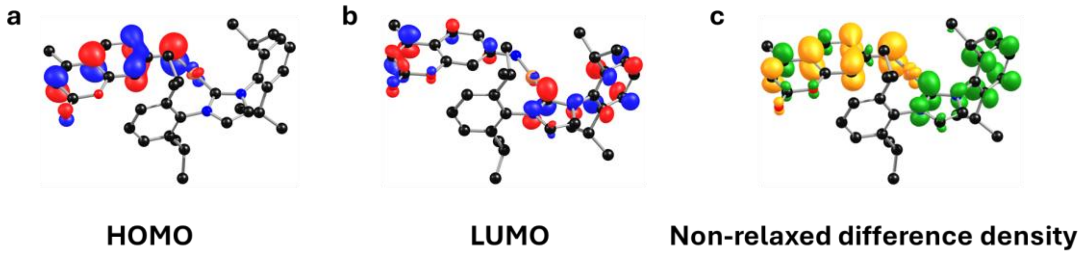

Figure S43: Highest occupied molecular orbital (a, HOMO), lowest unoccupied molecular orbital (b, LUMO) (contours at 0.05 a.u.) and non-relaxed difference density (c, contours at 0.002 a.u.) between the lowest energy excited singlet and the ground state of compound **6** (PBE0/def2-TZVP). For the latter, orange color indicates a surplus of electron density for the ground state, green for the excited state. Hydrogen atoms are omitted for clarity. Carbon black, nitrogen blue, oxygen red, copper orange.

Table S3: Ten lowest energy singlet vertical excitations (PBE0/def2-TZVP) of 7-amino-4-methylcoumarin. HOMO – Highest occupied molecular orbital, LUMO – Lowest unoccupied molecular orbital.

	Energy / nm	Oscillator strength / Velocity representation	Contributions (> 10 %)	Orbital pair contribution
1	302.176	0.35278	HOMO-LUMO	94.2
2	269.425	0.00640	HOMO-1-LUMO	56.5
			HOMO-LUMO+1	39.3
3	260.815	0.00005	HOMO-2-LUMO	92.9
4	245.503	0.04067	HOMO-LUMO+1	51.7
			HOMO-1-LUMO	41.7
5	221.272	0.01313	HOMO-LUMO+2	73.7
			HOMO-3-LUMO	17.6
6	211.202	0.01187	HOMO-LUMO+3	81.3
			HOMO-3-LUMO	13.2
7	206.013	0.16064	HOMO-3-LUMO	48.9
			HOMO-1-LUMO+1	24.9
8	196.017	0.01529	HOMO-2-LUMO+1	77.7
			HOMO-2-LUMO+2	11.2
9	194.353	0.52070	HOMO-1-LUMO+1	43.4
			HOMO-LUMO+4	18.3
10	193.165	0.13547	HOMO-LUMO+4	78.2
			HOMO-1-LUMO+1	10.4

Table S4: Ten lowest energy singlet vertical excitations (PBE0/def2-TZVP) of **1**. HOMO – Highest occupied molecular orbital, LUMO – Lowest unoccupied molecular orbital.

	Energy / nm	Oscillator strength / Velocity representation	Contributions (> 10 %)	Orbital pair contribution
1	382.223	0.35464	HOMO-LUMO	95.7
2	347.188	0.02626	HOMO-LUMO+1	97.2
3	342.536	0.00000	HOMO-LUMO+2	98.7
4	330.447	0.06039	HOMO-LUMO+3	94.6
5	315.485	0.00006	HOMO-LUMO+5	86.5
			HOMO-LUMO+4	12.0
6	312.336	0.02154	HOMO-LUMO+4	83.5
			HOMO-LUMO+5	11.6

7	287.254	0.03979	HOMO-LUMO+8	66.8
			HOMO-1-LUMO	24.7
8	278.817	0.00258	HOMO-LUMO+7	77.7
			HOMO-LUMO+9	17.1
9	278.682	0.00516	HOMO-LUMO+6	97.3
10	267.539	0.08195	HOMO-1-LUMO	60.2
			HOMO-LUMO+8	27.5

Table S5: Ten lowest energy singlet vertical excitations (PBE0/def2-TZVP) of **1** with perpendicularly oriented coumarin and NHC ligand planes. HOMO – Highest occupied molecular orbital, LUMO – Lowest unoccupied molecular orbital.

	Energy / nm	Oscillator strength / Velocity representation	Contributions (> 10 %)	Orbital pair contribution
1	390.165	0.00019	HOMO-LUMO	92.3
2	357.452	0.35326	HOMO-LUMO+1	95.8
3	348.292	0.00021	HOMO-LUMO+2	95.1
4	343.257	0.01205	HOMO-LUMO+3	98.8
5	333.847	0.01233	HOMO-LUMO+4	99.1
6	329.653	0.00043	HOMO-LUMO+5	94.6
7	291.397	0.01040	HOMO-LUMO+7	47.9
			HOMO-LUMO+6	42.7
8	286.881	0.01045	HOMO-LUMO+6	56.4
			HOMO-LUMO+7	33.3
9	269.195	0.03078	HOMO-LUMO+9	48.2
			HOMO-1-LUMO+1	32.6
			HOMO-LUMO+7	13.2
10	263.423	0.00777	HOMO-LUMO+8	46.1
			HOMO-LUMO+10	43.0

Table S6: Ten lowest energy singlet vertical excitations (PBE0/def2-TZVP) of **2**. HOMO – Highest occupied molecular orbital, LUMO – Lowest unoccupied molecular orbital.

	Energy / nm	Oscillator strength / Velocity representation	Contributions (> 10 %)	Orbital pair contribution
1	311.752	0.25062	HOMO-LUMO	95.9
2	285.882	0.06345	HOMO-LUMO+1	89.0
3	279.451	0.00844	HOMO-1-LUMO	63.3
4	264.546	0.00006	HOMO-12-LUMO	57.3

			HOMO-3-LUMO	16.0
			HOMO-2-LUMO	11.7
5	262.099	0.02854	HOMO-LUMO+2	62.4
			HOMO-1-LUMO	11.2
6	261.446	0.0048	HOMO-3-LUMO	38.2
			HOMO-2-LUMO	23.9
			HOMO-12-LUMO	18.6
			HOMO-LUMO+2	10.3
7	259.425	0.00073	HOMO-2-LUMO	53.0
			HOMO-3-LUMO	31.1
8	254.604	0.02145	HOMO-LUMO+6	32.3
			HOMO-LUMO+4	20.1
9	248.208	0.00273	HOMO-6-LUMO	45.6
			HOMO-5-LUMO	16.2
			HOMO-LUMO+3	12.3
10	247.808	0.00521	HOMO-7-LUMO	22.8
			HOMO-LUMO+3	22.6
			HOMO-LUMO+8	17.3
			HOMO-8-LUMO	11.0

Table S7: Ten lowest energy singlet vertical excitations (PBE0/def2-TZVP) of **3**. HOMO – Highest occupied molecular orbital, LUMO – Lowest unoccupied molecular orbital.

	Energy / nm	Oscillator strength / Velocity representation	Contributions (> 10 %)	Orbital pair contribution
1	332.884	0.09106	HOMO-LUMO+1	70.0
			HOMO-LUMO	23.5
2	330.15	0.39283	HOMO-LUMO	71.3
			HOMO-LUMO+1	24.3
3	319.613	0.05437	HOMO-LUMO+2	93.8
4	293.418	0.07495	HOMO-LUMO+3	86.3
5	283.76	0.04082	HOMO-1-LUMO	56.2
			HOMO-1-LUMO+1	29.1
6	280.874	0.07482	HOMO-1-LUMO+1	48.8
			HOMO-1-LUMO	36.6
7	276.926	0.0226	HOMO-2-LUMO	55.6
			HOMO-LUMO+5	25.2
8	270.42	0.08009	HOMO-1-LUMO+2	73.8
			HOMO-1-LUMO+1	15.2

9	264.504	0.00765	HOMO-4-LUMO	76.9
			HOMO-4-LUMO+3	10.1
10	258.141	0.00931	HOMO-LUMO+4	62.2
			HOMO-LUMO+5	12.3

Table S8: Ten lowest energy singlet vertical excitations (PBE0/def2-TZVP) of **4**. HOMO – Highest occupied molecular orbital, LUMO – Lowest unoccupied molecular orbital.

	Energy / nm	Oscillator strength / Velocity representation	Contributions (> 10 %)	Orbital pair contribution
1	361.900	0.01476	HOMO-LUMO	54.3
			HOMO-LUMO+2	22.6
			HOMO-LUMO+1	11.9
2	348.441	0.01349	HOMO-LUMO	36.8
			HOMO-LUMO+2	34.9
			HOMO-LUMO+1	16.6
3	347.582	0.21570	HOMO-LUMO+1	61.3
			HOMO-LUMO+2	31.4
4	341.155	0.01224	HOMO-LUMO+3	80.2
5	333.503	0.01465	HOMO-LUMO+4	86.4
6	324.907	0.08348	HOMO-LUMO+6	80.9
7	323.626	0.07109	HOMO-LUMO+5	74.5
8	318.394	0.04031	HOMO-LUMO+7	82.8
9	305.422	0.00823	HOMO-LUMO+8	88.6
10	304.243	0.09054	HOMO-LUMO+9	86.6

Table S9: Ten lowest energy singlet vertical excitations (PBE0/def2-TZVP) of **5**. HOMO – Highest occupied molecular orbital, LUMO – Lowest unoccupied molecular orbital.

	Energy / nm	Oscillator strength / Velocity representation	Contributions (> 10 %)	Orbital pair contribution
1	381.888	0.00185	HOMO-LUMO	86.6
			HOMO-LUMO+1	10.5
2	375.143	0.00123	HOMO-LUMO+1	81.6
			HOMO-LUMO	11.6
3	368.416	0.01341	HOMO-LUMO+2	88.2
4	351.985	0.03666	HOMO-LUMO+3	58.3
			HOMO-LUMO+4	21.1
5	344.120	0.08950	HOMO-LUMO+4	63.7

			HOMO-LUMO+3	28.5
6	330.421	0.12288	HOMO-LUMO+5	87.5
7	326.496	0.08223	HOMO-LUMO+6	76.0
			HOMO-LUMO+4	11.4
8	319.220	0.04787	HOMO-LUMO+7	61.8
			HOMO-LUMO+8	17.6
9	315.578	0.12106	HOMO-LUMO+8	49.0
			HOMO-LUMO+9	23.5
10	312.249	0.00959	HOMO-LUMO+9	56.0
			HOMO-LUMO+8	16.8
			HOMO-LUMO+7	13.7

Table S10: Ten lowest energy singlet vertical excitations (PBE0/def2-TZVP) of **6**. HOMO – Highest occupied molecular orbital, LUMO – Lowest unoccupied molecular orbital.

	Energy / nm	Oscillator strength / Velocity representation	Contributions (> 10 %)	Orbital pair contribution
1	392.643	0.26392	HOMO-LUMO	89.7
2	363.191	0.03894	HOMO-LUMO+1	90.7
3	360.303	0.00003	HOMO-LUMO+2	98.9
4	342.019	0.09627	HOMO-LUMO+3	92.9
5	327.465	0.00007	HOMO-LUMO+5	83.4
			HOMO-LUMO+4	15.4
6	324.514	0.02116	HOMO-LUMO+4	80.5
			HOMO-LUMO+5	14.9
7	291.855	0.01097	HOMO-2-LUMO	62.2
			HOMO-2-LUMO+3	20.4
8	289.491	0.04048	HOMO-LUMO+8	74.3
			HOMO-1-LUMO	13.1
9	286.662	0.00479	HOMO-LUMO+6	97.4
10	284.537	0.0103	HOMO-LUMO+7	70.9
			HOMO-LUMO+9	19.8

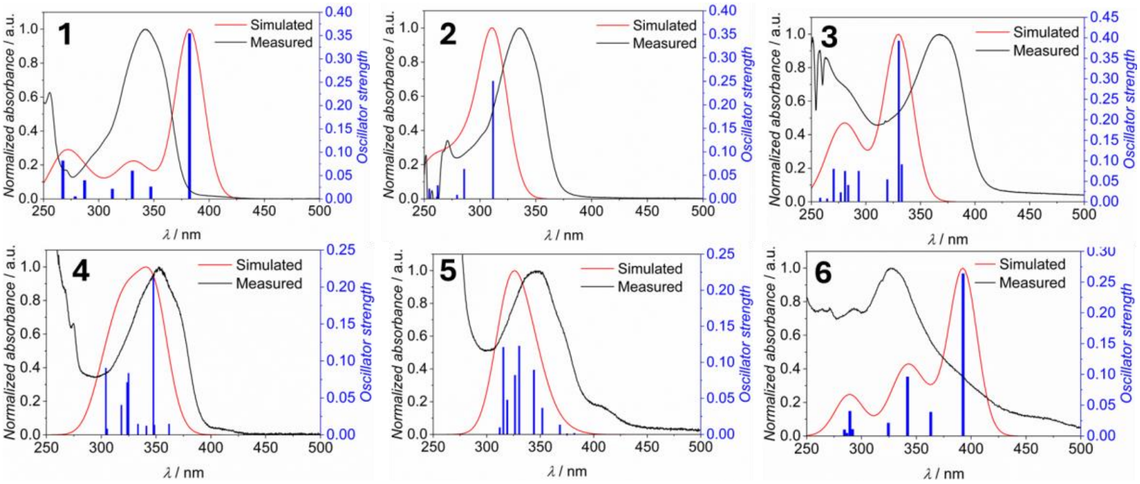

Figure S44: Measured UV/Vis absorption spectra of complexes **1**-**6** in DCM superimposed with simulated spectra (FWHM 30 nm) based on the calculated oscillator strengths (velocity representation) of the ten lowest energy singlet vertical excitations (PBE0/def2-TZVP, refer to Tables S4 and S6-S10).

Table S11: Mulliken population analysis of the HOMO of 7-amino-4-methylcoumarin. Labels of atoms refer to numbering in the provided coordinate file optimized-structures.txt.

atom	total	s	p	d	f
1c	0.02782	0.00000	0.01050	0.01667	0.00066
2c	0.33267	-0.00000	0.33243	0.00007	0.00018
4c	0.19997	0.00012	0.18119	0.01757	0.00110
9c	0.04330	-0.00002	0.02007	0.02246	0.00079
10c	0.35824	0.00008	0.35624	0.00159	0.00033
11c	0.08141	0.00009	0.06876	0.01187	0.00070
12c	0.04922	0.00017	0.02845	0.01979	0.00081
14c	0.24876	-0.00034	0.24424	0.00441	0.00045
16n	0.37511	0.00978	0.36281	0.00248	0.00004
18c	0.10138	-0.00052	0.09579	0.00562	0.00049
20o	0.14808	-0.00000	0.14824	-0.00016	-0.00001
21o	0.01243	0.00000	0.01198	0.00042	0.00003

Table S12: Mulliken population analysis of the LUMO of 7-amino-4-methylcoumarin. Labels of atoms refer to numbering in the provided coordinate file optimized-structures.txt.

atom	total	s	p	d	f
1c	0.18141	0.00000	0.15575	0.02485	0.00081
2c	0.35933	0.00001	0.32970	0.02873	0.00089
4c	0.18249	-0.00025	0.17828	0.00425	0.00020
5c	0.02057	0.00000	-0.00097	0.02072	0.00082
6h	0.02942	0.02905	0.00037		
8h	0.02891	0.02854	0.00037		
9c	0.44485	-0.00001	0.43207	0.01229	0.00050
10c	0.05620	0.00005	0.02718	0.02810	0.00086
11c	0.06152	-0.00012	0.05680	0.00467	0.00017
12c	0.24996	0.00008	0.24760	0.00222	0.00007
14c	0.04510	-0.00019	0.02608	0.01860	0.00060
16n	0.05753	0.00174	0.05191	0.00378	0.00009
18c	0.07127	0.00026	0.06233	0.00824	0.00043
20o	0.13828	-0.00000	0.13789	0.00038	0.00001
21o	0.06046	-0.00000	0.06025	0.00020	0.00001

Table S13: Mulliken population analysis of the HOMO of complex **1**. Labels of atoms refer to numbering in the provided coordinate file optimized-structures.txt.

161a energy/a.u.: -0.1772816

atom	total	s	p	d	f
1au	0.09316	0.00000	0.03617	0.05605	0.00094
6o	0.07100	0.00000	0.07111	-0.00010	-0.00000
9n	0.57435	0.00000	0.57310	0.00130	-0.00005

11c	0.22161	-0.00000	0.22033	0.00104	0.00023
15c	0.02622	0.00000	0.00910	0.01640	0.00073
16c	0.02816	0.00000	0.00836	0.01912	0.00068
18c	0.22571	0.00000	0.22434	0.00112	0.00025
25c	0.31128	-0.00000	0.31115	0.00001	0.00013
27c	0.02027	-0.00000	0.00020	0.01940	0.00067
45c	0.10663	0.00000	0.07095	0.03418	0.00150
47c	0.02436	-0.00000	0.01378	0.01018	0.00040
48c	0.24950	-0.00000	0.24912	0.00028	0.00011

Table S14: Mulliken population analysis of the LUMO of complex **1**. Labels of atoms refer to numbering in the provided coordinate file optimized-structures.txt.

162a energy/a.u.: -0.0343293

atom	total	s	p	d	f
1au	0.20140	0.00000	0.19344	0.00728	0.00068
2o	0.03294	0.00000	0.03277	0.00017	0.00000
3n	0.05083	-0.00000	0.04571	0.00510	0.00002
4n	0.06057	0.00000	0.05661	0.00393	0.00003
5c	0.28511	-0.00000	0.27847	0.00647	0.00016
6o	0.06898	-0.00000	0.06872	0.00025	0.00001
7c	0.02021	-0.00000	0.01446	0.00557	0.00018
9n	0.02344	0.00000	0.01812	0.00524	0.00008
11c	0.03694	-0.00000	0.03253	0.00413	0.00028
13c	0.03683	-0.00000	0.03216	0.00448	0.00019
15c	0.03513	0.00000	0.03266	0.00239	0.00008
16c	0.13330	-0.00000	0.13220	0.00107	0.00003
18c	0.02425	0.00000	0.01343	0.01049	0.00033

25c	0.02547	0.00000	0.01035	0.01467	0.00045
26c	0.01749	0.00415	0.01233	0.00100	0.00001
27c	0.23456	0.00000	0.22878	0.00556	0.00023
30c	0.01350	0.00000	0.00373	0.00950	0.00027
31c	0.05208	0.00047	0.04982	0.00177	0.00002
37c	0.01103	0.00000	-0.00057	0.01116	0.00044
38h	0.01565	0.01544	0.00021		
40h	0.01564	0.01543	0.00021		
43c	0.05229	0.00040	0.05011	0.00176	0.00002
45c	0.10657	-0.00000	0.10390	0.00260	0.00007
46c	0.05474	0.00287	0.04908	0.00277	0.00002
47c	0.09700	-0.00000	0.08420	0.01241	0.00039
48c	0.17422	-0.00000	0.15892	0.01483	0.00047
50c	0.01730	0.00373	0.01257	0.00100	0.00001
53c	0.05434	0.00287	0.04866	0.00279	0.00002
56c	-0.01386	-0.01534	0.00142	0.00007	0.00000
60c	-0.01891	-0.01910	0.00002	0.00016	0.00001
72c	-0.01374	-0.01524	0.00143	0.00007	0.00000
76c	-0.01913	-0.01925	-0.00005	0.00016	0.00001

Table S15: Mulliken population analysis of the HOMO of complex **1** with perpendicularly oriented ligand planes. Labels of atoms refer to numbering in the provided coordinate file optimized-structures.txt.

atom	total	s	p	d	f
1c	0.02427	-0.00000	0.01327	0.01059	0.00042
2c	0.25778	0.00000	0.25742	0.00025	0.00011

4c	0.10439	-0.00000	0.06865	0.03424	0.00150
9c	0.02086	-0.00000	0.00022	0.01995	0.00068
10c	0.31971	-0.00000	0.31957	0.00000	0.00014
11c	0.02749	0.00000	0.01028	0.01647	0.00073
12c	0.02887	-0.00000	0.00847	0.01971	0.00069
14c	0.23377	0.00000	0.23238	0.00114	0.00025
16n	0.58158	-0.00000	0.58043	0.00120	-0.00005
18c	0.22047	-0.00000	0.21913	0.00110	0.00023
20o	0.07432	0.00000	0.07444	-0.00011	-0.00000
22au	0.08096	-0.00000	0.02428	0.05581	0.00087
25c	0.01155	0.00000	0.00702	0.00447	0.00005

Table S16: Mulliken population analysis of the LUMO of complex **1** with perpendicularly oriented ligand planes. Labels of atoms refer to numbering in the provided coordinate file optimized-structures.txt.

atom	total	s	p	d	f
4c	-0.03394	-0.01682	-0.01665	-0.00050	0.00004
16n	0.01969	-0.00218	0.02012	0.00173	0.00002
18c	0.01441	-0.01806	0.03180	0.00066	0.00000
19h	0.03093	0.03006	0.00088		
22au	0.29393	0.00430	0.25599	0.03286	0.00078
23n	0.10923	0.00213	0.09490	0.01210	0.00010
24n	0.11267	0.00152	0.09917	0.01187	0.00011
25c	0.52903	-0.00367	0.52202	0.01038	0.00031
26c	0.04561	0.00075	0.03401	0.01048	0.00036
28c	0.05535	0.00209	0.04293	0.00996	0.00036
30c	0.06341	0.00119	0.04954	0.01226	0.00043

31c	0.08503	0.00240	0.07956	0.00304	0.00002
34h	0.02025	0.01987	0.00038		
35c	0.02184	0.00449	0.00804	0.00918	0.00013
36c	0.06384	-0.00018	0.06041	0.00355	0.00006
38c	0.06176	0.00067	0.04731	0.01336	0.00041
39c	0.09515	0.00214	0.09004	0.00295	0.00002
45c	0.06054	-0.00020	0.05645	0.00421	0.00008
47c	0.01513	0.00020	0.00736	0.00739	0.00018
49c	0.02142	0.00375	0.00796	0.00958	0.00013
50c	0.11289	0.00676	0.10328	0.00281	0.00003
53c	0.12038	0.00505	0.11268	0.00263	0.00002
54c	0.01085	0.00009	0.00339	0.00718	0.00018
56c	-0.05560	-0.06340	0.00691	0.00088	0.00001
57h	0.01257	0.01243	0.00014		
58h	0.01245	0.01152	0.00093		
59h	0.02709	0.02672	0.00037		
60c	-0.01208	-0.00940	-0.00309	0.00040	0.00001
68c	0.01303	-0.01184	0.02067	0.00400	0.00020
69h	0.01902	0.01866	0.00036		
72c	-0.01008	-0.00765	-0.00281	0.00037	0.00001
76c	-0.05509	-0.06275	0.00679	0.00086	0.00001
77h	0.01157	0.01145	0.00012		
78h	0.02672	0.02635	0.00037		
79h	0.01220	0.01129	0.00090		

Table S17: Mulliken population analysis of the LUMO+1 of complex **1** with perpendicularly oriented ligand planes. Labels of atoms refer to numbering in the provided coordinate file optimized-structures.txt.

atom	total	s	p	d	f
1c	0.18423	0.00000	0.16169	0.02186	0.00067
2c	0.28977	-0.00000	0.26096	0.02794	0.00087
4c	0.14399	-0.00000	0.13875	0.00497	0.00028
5c	0.02108	-0.00000	-0.00135	0.02162	0.00081
6h	0.03110	0.03068	0.00042		
8h	0.03088	0.03046	0.00042		
9c	0.42797	-0.00000	0.41932	0.00831	0.00034
10c	0.03204	0.00000	0.00590	0.02530	0.00084
11c	0.05543	-0.00000	0.05253	0.00279	0.00011
12c	0.21918	-0.00000	0.21719	0.00189	0.00010
14c	0.06267	0.00000	0.04695	0.01521	0.00050
16n	0.07293	-0.00000	0.06912	0.00375	0.00006
18c	0.03410	0.00000	0.02763	0.00608	0.00039
20o	0.12968	0.00000	0.12922	0.00044	0.00001
21o	0.05682	0.00000	0.05643	0.00038	0.00001
22au	0.06294	0.00000	0.05990	0.00287	0.00017
25c	0.03600	0.00001	0.03381	0.00217	0.00001
30c	0.02236	0.01524	0.00579	0.00131	0.00002
31c	0.02237	0.00435	0.01763	0.00039	-0.00000
35c	-0.01341	-0.01421	-0.00054	0.00132	0.00002
38c	0.01976	0.01662	0.00212	0.00100	0.00002
39c	0.01741	0.00417	0.01300	0.00024	-0.00000
47c	0.01071	0.00463	0.00531	0.00075	0.00002

49c	-0.01419	-0.01434	-0.00076	0.00089	0.00002
50c	0.01172	-0.00441	0.01581	0.00032	-0.00000
54c	0.01032	0.00425	0.00486	0.00118	0.00003

Table S18: Mulliken population analysis of the HOMO of complex **2**. Labels of atoms refer to numbering in the provided coordinate file optimized-structures.txt.

276a energy/a.u.: -0.3027792

atom	total	s	p	d	f
1au	0.14871	0.07140	0.01019	0.06655	0.00057
6o	0.12050	-0.00000	0.12062	-0.00012	-0.00000
9n	0.24754	0.00006	0.24512	0.00230	0.00006
10c	0.08631	0.00001	0.08178	0.00402	0.00050
14c	0.05570	0.00001	0.04564	0.00948	0.00058
15c	0.06374	-0.00002	0.04829	0.01482	0.00065
17c	0.17721	-0.00017	0.17106	0.00583	0.00048
24c	0.29152	-0.00002	0.28960	0.00166	0.00028
26c	0.04143	-0.00000	0.02233	0.01846	0.00064
44c	0.20397	0.00024	0.19082	0.01216	0.00076
46c	0.02325	0.00000	0.00849	0.01423	0.00053
47c	0.28654	-0.00001	0.28628	0.00010	0.00016
88au	0.16809	0.08653	0.01147	0.06946	0.00063

Table S19: Mulliken population analysis of the LUMO of complex **2**. Labels of atoms refer to numbering in the provided coordinate file optimized-structures.txt.

277a energy/a.u.: -0.1320152

atom	total	s	p	d	f
1au	0.02120	0.01835	0.00249	0.00019	0.00017

2o	0.05827	0.00000	0.05802	0.00024	0.00001
6o	0.12773	0.00000	0.12725	0.00046	0.00001
9n	0.01974	-0.00000	0.01386	0.00574	0.00014
10c	0.09867	0.00013	0.09021	0.00791	0.00042
14c	0.06511	-0.00035	0.05784	0.00738	0.00024
15c	0.25387	0.00004	0.25131	0.00248	0.00004
17c	0.02895	-0.00024	0.00864	0.01995	0.00061
24c	0.07472	0.00001	0.04710	0.02683	0.00078
26c	0.40775	-0.00006	0.39457	0.01271	0.00053
36c	0.01818	0.00003	-0.00016	0.01758	0.00074
37h	0.02332	0.02307	0.00025		
39h	0.02503	0.02476	0.00027		
44c	0.20189	0.00007	0.19730	0.00445	0.00006
46c	0.17192	0.00001	0.14632	0.02480	0.00078
47c	0.34355	-0.00008	0.31676	0.02606	0.00081
88au	0.03065	0.02815	0.00212	0.00016	0.00021
125c	-0.01237	-0.00571	-0.00677	0.00011	0.00000
126c	-0.02245	-0.01523	-0.00730	0.00008	0.00000
128c	0.01297	0.01203	0.00082	0.00011	0.00000
132c	0.01785	0.01023	0.00764	-0.00002	0.00000

Table S20: Mulliken population analysis of the HOMO of complex **3**. Labels of atoms refer to numbering in the provided coordinate file optimized-structures.txt.

199a energy/a.u.: -0.2733712

atom	total	s	p	d	f
1au	0.14777	0.10624	0.00413	0.03644	0.00097
2au	0.05869	-0.00004	0.00470	0.05365	0.00038

3au	0.14197	0.09903	0.00456	0.03747	0.00092
4n	0.28040	0.00001	0.27818	0.00219	0.00002
5o	0.01214	0.00000	0.01171	0.00039	0.00003
6o	0.12096	0.00000	0.12109	-0.00013	-0.00000
12c	0.07465	0.00000	0.06414	0.00992	0.00059
13c	0.16461	0.00005	0.14745	0.01628	0.00084
16c	0.29249	-0.00001	0.29107	0.00116	0.00028
17c	0.08299	-0.00001	0.07724	0.00532	0.00045
20c	0.21673	-0.00001	0.21373	0.00262	0.00039
22c	0.26346	-0.00000	0.26328	0.00004	0.00014
24c	0.01358	-0.00001	0.01097	0.00259	0.00004
27c	0.03080	-0.00000	0.01368	0.01645	0.00068
31c	0.03341	0.00000	0.01481	0.01796	0.00065
55c	0.02335	0.00000	0.00922	0.01358	0.00055

Table S21: Mulliken population analysis of the HOMO of complex **4**. Labels of atoms refer to numbering in the provided coordinate file optimized-structures.txt.

280a energy/a.u.: -0.2778116

atom	total	s	p	d	f
1au	0.12362	0.10304	0.00240	0.01725	0.00093
2au	0.05690	0.01826	0.00655	0.03160	0.00048
3au	0.07174	0.03751	0.00429	0.02935	0.00060
4p	0.01781	0.00028	0.01419	0.00314	0.00020
5p	0.01486	0.00001	0.01138	0.00325	0.00022
6p	0.01097	0.00171	0.00922	0.00001	0.00003
7n	0.25234	0.00012	0.24921	0.00299	0.00003
8o	0.01574	0.00000	0.01528	0.00043	0.00003

9o	0.14032	0.00000	0.14046	-0.00014	-0.00000
10c	0.21958	-0.00027	0.21546	0.00397	0.00042
12c	0.18969	0.00134	0.17299	0.01453	0.00084
25c	0.04138	0.00008	0.02373	0.01686	0.00071
27c	0.08317	0.00002	0.07287	0.00967	0.00061
29c	0.29529	-0.00000	0.29502	0.00011	0.00016
32c	0.30905	-0.00008	0.30704	0.00177	0.00032
45c	0.07218	-0.00017	0.06607	0.00580	0.00048
63c	0.04324	0.00000	0.02316	0.01938	0.00070
68c	0.02496	0.00000	0.00875	0.01559	0.00062

Table S22: Mulliken population analysis of the HOMO of complex **5**. Labels of atoms refer to numbering in the provided coordinate file optimized-structures.txt.

280a energy/a.u.: -0.2777415

atom	total	s	p	d	f
1au	0.04892	0.00954	0.00630	0.03257	0.00050
2au	0.13123	0.11238	0.00367	0.01426	0.00091
3au	0.07735	0.04607	0.00411	0.02660	0.00057
6p	0.01752	0.00029	0.01237	0.00464	0.00022
7n	0.24657	0.00014	0.24337	0.00303	0.00003
82c	0.08366	0.00002	0.07339	0.00964	0.00061
83c	0.31033	-0.00007	0.30823	0.00185	0.00032
84c	0.04283	0.00003	0.02530	0.01679	0.00071
86c	0.21991	-0.00004	0.21552	0.00402	0.00042
88c	0.19145	0.00126	0.17502	0.01433	0.00083
89c	0.06988	-0.00031	0.06392	0.00579	0.00048
91c	0.04459	0.00000	0.02438	0.01951	0.00070

92c	0.29838	-0.00000	0.29809	0.00012	0.00016
94c	0.02495	0.00000	0.00853	0.01580	0.00062
95o	0.01625	0.00000	0.01579	0.00043	0.00003
100o	0.14254	0.00000	0.14268	-0.00013	-0.00000

Table S23: Mulliken population analysis of the HOMO of complex **6**. Labels of atoms refer to numbering in the provided coordinate file optimized-structures.txt.

166a energy/a.u.: -0.1725018

atom	total	s	p	d	f
1cu	0.09560	0.00000	0.02054	0.07504	0.00001
3n	0.58076	-0.00000	0.57974	0.00105	-0.00004
7o	0.06836	-0.00000	0.06846	-0.00010	-0.00000
8c	0.02401	-0.00000	0.00663	0.01667	0.00071
11c	0.09303	-0.00000	0.05695	0.03463	0.00146
12c	0.30862	-0.00000	0.30846	0.00004	0.00012
13c	0.02709	0.00000	0.00730	0.01913	0.00066
16c	0.02039	0.00000	0.00039	0.01935	0.00066
19c	0.24890	0.00000	0.24846	0.00033	0.00011
25c	0.23116	0.00000	0.23011	0.00084	0.00021
27c	0.22941	-0.00000	0.22808	0.00110	0.00023
29c	0.02465	-0.00000	0.01416	0.01010	0.00040

Table S24: Mulliken population analysis of the LUMO of complex **6**. Labels of atoms refer to numbering in the provided coordinate file optimized-structures.txt.

167a energy/a.u.: -0.0322946

atom	total	s	p	d	f
1cu	0.08240	0.00000	0.07788	0.00451	0.00001

2o	0.01961	0.00000	0.01949	0.00011	0.00000
3n	0.01430	0.00000	0.01128	0.00298	0.00004
5n	0.07299	0.00000	0.06941	0.00355	0.00003
6n	0.04879	-0.00000	0.04240	0.00638	0.00002
7o	0.04179	-0.00000	0.04163	0.00016	0.00000
8c	0.02074	-0.00000	0.01920	0.00149	0.00005
9c	0.01211	-0.00000	0.00384	0.00807	0.00020
11c	0.06616	-0.00000	0.06448	0.00165	0.00004
12c	0.01498	0.00000	0.00596	0.00875	0.00027
13c	0.08268	-0.00000	0.08202	0.00064	0.00002
15c	0.29709	0.00000	0.29034	0.00660	0.00016
16c	0.14120	-0.00000	0.13775	0.00331	0.00014
17c	0.06239	0.00000	0.05741	0.00478	0.00021
19c	0.10375	0.00000	0.09450	0.00897	0.00028
25c	0.02252	-0.00000	0.01975	0.00261	0.00017
27c	0.01575	0.00000	0.00878	0.00676	0.00020
29c	0.05891	-0.00000	0.05124	0.00744	0.00023
31c	0.01558	0.00636	0.00811	0.00109	0.00001
34c	-0.02436	-0.02512	0.00046	0.00029	0.00000
37h	0.02450	0.02404	0.00046		
42c	0.02149	-0.00000	-0.00151	0.02264	0.00035
43c	0.01205	-0.00000	-0.00070	0.01245	0.00029
45c	0.14508	0.00009	0.13968	0.00524	0.00007
47c	0.03052	-0.00507	0.02920	0.00617	0.00023
53c	-0.03832	-0.03744	-0.00149	0.00060	0.00001
54h	0.03488	0.03423	0.00066		

56h	0.01112	0.01091	0.00021		
57c	0.13625	0.00328	0.12647	0.00645	0.00005
58c	0.14216	0.00012	0.13654	0.00542	0.00008
60c	0.02987	-0.00548	0.02883	0.00628	0.00023
66c	-0.03862	-0.03811	-0.00115	0.00063	0.00001
67h	0.03513	0.03446	0.00067		
68h	0.01169	0.01147	0.00022		
70c	0.13254	0.00323	0.12258	0.00667	0.00006
75c	0.01561	0.00609	0.00841	0.00109	0.00001
84c	-0.02411	-0.02494	0.00053	0.00029	0.00000
85h	0.02472	0.02426	0.00046		

VIII. References

1. S. Gaillard, A. M. Z. Slawin, S. P. Nolan, *Chem. Commun.* **2010**, *46,* 2742-2744.
2. G. A. Price, A. K. Brisdon, K. R. Flower, R. G. Pritchard, P. Quayle, *Tetrahedron Lett.* **2014**, *55,* 151-154.
3. R. S. Ramón, S. Gaillard, A. Poater, L. Cavallo, A. M. Slawin, S. P. Nolan, *Chem. - Eur. J.* **2011**, *17,* 1238-1246.
4. H. Ube, Q. Zhang, M. Shionoya, *Organometallics* **2018**, *37,* 2007-2009.
5. A. N. Nesmeyanov, E. G. Perevalova, Y. T. Struchkov, M. Y. Antipin, K. I. Grandberg, V. P. Dyadhenko, *J. Organomet. Chem.* **1980**, *201,* 343-349.
6. O. Crespo, M. C. Gimeno, A. Laguna, C. Larraz, M. D. Villacampa, *Chem. - Eur. J.* **2007**, *13,* 235-246.
7. G. Sheldrick, *Acta Cryst. A* **2008**, *64*, 112-122.
8. G. Sheldrick, *Acta Cryst. C* **2015**, *71*, 3-8.
9. O. V. Dolomanov, L.J. Bourhis, R. J. Gildea, J. A. K. Howard, H. Puschmann, *J. Appl. Crystallogr*. **2009**, *42*, 339-341.
10. TURBOMOLE V. 7.8 University of Karlsruhe and Forschungszentrum Karlsruhe GmbH 1989-2007. TURBOMOLE GmbH since 2007. Available from https://turbomole.org.
11. Y. J. Franzke, C. Holzer, J. H. Andersen, T. Begušić, F. Bruder, S. Coriani, F. Della Sala, E. Fabiano, D. A. Fedotov, S. Fürst, S. Gillhuber, R. Grotjahn, M. Kaupp, M. Kehry, M. Krstić, F. Mack, S. Majumdar, B. D. Nguyen, S. M. Parker, F. Pauly, A. Pausch, E. Perlt, G. S. Phun, A. Rajabi, D. Rappoport, B. Samal, T. Schrader, M. Sharma, E. Tapavicza, R. S. Treß, V. Voora, A. Wodyński, J. M. Yu, B. Zerulla, F. Furche, C. Hättig, M. Sierka, D. P. Tew, F. Weigend, *J. Chem. Theory Comput.* **2023**, *19*, 6859-6890.
12. J. P. Perdew, K. Burke, M. Ernzerhof, *Phys. Rev. Lett.* **1996**, *77*, 3865.
13. J. P. Perdew, M. Ernzerhof, K. Burke, *Chem. Phys.* **1996**, *105*, 9982-9985.
14. C. Adamo, V. Barone, *Chem. Phys.* **1999**, *110*, 6158-6170.
15. F. Weigend, R. Ahlrichs, *Phys. Chem. Chem. Phys.* **2005**, *7*, 3297-3305.
16. D. Andrae, U. Haeussermann, M. Dolg, H. Stoll, H. Preuss, *Theor. Chim. Acta* **1990**, *77*, 123-141.
17. F. Weigend, *Phys. Chem. Chem. Phys.* **2006**, *8*, 1057-1065.
18. M. Sierka, A. Hogekamp, R. Ahlrichs, *Chem. Phys.* **2003**, *118*, 9136-9148.
19. E. Caldeweyher, C. Bannwarth, S. Grimme, *Chem. Phys.* **2017**, *147*, 034112.
20. O. Treutler, R. Ahlrichs, *Chem. Phys.* **1995**, *102*, 346-354.
21. X.-X. Yang, I. Issac, S. Lebedkin, M. Kühn, F. Weigend, D. Fenske, O. Fuhr, A. Eichhöfer, *Chem. Commun.* **2014**, *50*, 11043-11045.
22. J. C. de Mello, H. F. Wittmann, R. H. Friend, Adv. Mater. **1997**, *9,* 230-232.
